# Supplementary material for: Impact of the COVID-19 pandemic on young people from black and mixed ethnic groups’ mental health in West London: a qualitative study
Source: BMJ Open. 2023 May 5;13(5):e071903. doi: 10.1136/bmjopen-2023-071903 (PMC10163329; doi:10.1136/bmjopen-2023-071903)
Supplement: Supplementary data [file bmjopen-2023-071903supp001.pdf]

Appendix A

Initial Interview Schedule

Introduce the research team and build rapport. Present the consent form and information sheet, go over ethics and take questions. Finally, explain rationale of the study, why we are here, aim to build trust.

Part 1- *Broad questions about impact of Covid-19*

1. How has your experience of the pandemic been?
    1.1. If mention education, how did it feel not going to school anymore?
    1.2. If mention home environment, how did it feel spending more time at home?
2. How were your relationships with your friends and family?
3. What were some of your favourite or least favourite things about covid?
4. How did covid impact your physical health?
    4.1. If mention close experience with covid, how did that made them feel?
5. How did Covid-19 impact your mental health?
    5.1. If mention of worry or such feelings, how did that feel like? what made it worse or better for you? If you opened up, who did you feel comfortable talking to?
6. Has your perception of the media, governments and institutions during the pandemic affected your trust in them?

Part 2- *Changes throughout the pandemic*

7. What about now, how have things changed since then?
    7.1. If no direction, what are the biggest challenges now? What were the biggest changes in your life?
8. How have your physical and mental health changed since lockdown?
    8.1. If need guidance, are they better or worse now?

9. How is your perception of the media, governments and institutions now, after the peak of the pandemic has passed and how has your trust in them changed?

Part 3- *Support needed to deal with these issues*

10. Did you receive any mental support during the pandemic?

    10.1. If so, how did you access it? What form did the support take and how did it make you feel?

    10.2. If not, did you feel like you would have needed some? what kind?

11. What aspects of your life do you feel you would need more support in?

12. What kind of support would you need now and how would you like to access it?

Debrief, thank you and share next steps of the research project.

Appendix B

Final Interview Schedule

Introductions - Researchers will introduce themselves, along with JED CEO and social worker and any student researchers present. Thank young people for taking part and joining the focus group. Build rapport. Walk through participant information sheet with young people, read verbatim each item on the consent form to ensure participants understand. Take questions.

Lay groundwork for why we're conducting this project, project aims, what we hope to learn and project outputs.

Express ethical considerations (right to withdraw at any time, don't have to share information they don't want to, don't have to answer all questions, confidentiality, trust, safe space to share, no right or wrong answers). Again, emphasize that researchers are here to learn from young people, in order to help other young people in the future as we navigate out of the COVID pandemic. Remind young people the session will be split into 2 parts and last between 30-40 minutes.

QUESTIONS

Part 1 – barriers to health access, impact of covid on health and changes

1. Thinking over this past year, how has it been for you?
    a. Prod: What are some things you are grateful for, or challenges that you faced?
2. Did you learn anything about yourself or others around you? How were you close relationships?
3. Thinking about this past year, has the pandemic affected the way you trust in others? Institutions, governments, community, people?
4. What were some highlights?
5. How has covid impacted your health – mentally and physically?

6. Thinking about pre-pandemic and now, what were some things that has changed for the better? Or for the worse?
7. Thinking about pre-pandemic and now, how has your health changed for the better? Or for the worse?
8. Thinking about some of your challenges, what are some examples of your struggles/challenges?
9. What did you enjoy or least enjoy during the pandemic?

Part 2 – Support that young people would like or need

1. What support did you receive during the pandemic, if any?
2. How was the support that you received, if any?
    a. How did you come across this support?
    b. What were the barriers to the support? If any?
    c. If no barriers, what made it accessible?
3. If you could get more support during the pandemic – and now – what might you want/need?
4. Thinking specifically about your health (physical/mental), what do you wish you had known? What support would you want?
5. What do you think are the key areas that you need most support on? Where might you go to get support?

Debrief and thank young people for their time. Inform next steps in project on co-creating workshops together

Appendix C

Patterns clusters

… : aim 1: mental health

Patterns of negative mental health
ISOLATION FROM OTHERS: the most
Isolation IIIIIIIIIIIII **Loneliness** IIIIIIII **Broken community** II **Lack of social interactions** IIIIIII **Loosing friendships** IIII
ILLNESS-RELATED DISTRESS: the 2nd most
COVID anxiety IIIIII **Death** IIII **Media overload** II **Overwhelming focus on physical** II
FAMILY ISSUES: sometimes
Home environment III
EDUCATION: sometimes
Online learning IIII
BAD MENTAL HEALTH: often
Overall hard MENTAL HEALTH IIIII
TRUST: often
Trust issues (friends/institutions) IIII
SADNESS: frequent
Sadness II **Depression** IIIII
STRESS: often
Stress and anxiety III **Overthinking** I **Fear** I
CLINICAL: frequent
Suicide/suicidal ideations III **Eating disorders/body image** IIIII **ADHD** I
LACK OF CONTROL: sometimes
Lack of usual coping/escape mechanisms II **Helpless** I
SELF-ESTEEM: frequent
Self-esteem IIIIII
APATHY/NUMB: frequent
Laziness III **Loss of structure** I **Lack of motivation** IIIII **Nonchalance** I **Boring** I **Tired** III
ANGER: often
Anger II **Frustration** IIII **Triggering** I

Young people III **Collective denial** I

Patterns of positive mental health
CLOSER INTERACTIONS: sometimes
Closer bonds (family, friends) III **Meeting people online** I
GRATEFULNESS: often
Grateful IIII
NEW HABITS: the most
New mindful practices IIIIIIII
SOLITUTDE: often
Alone time II **Resilience** II **Self-actualisation** IIII
EDUCATION: rarely
Time to study II

…: aim 2: changes with time

NOSTALGIA: rarely
Nostalgia I
SOCIAL INTERACTION HELPS: the most
After lockdown peak with seeing people again IIIIIIIIIIII
SELF-RELIANCE SO IMPROVEMENT WITH TIME: the 2nd most
Mental health improving because of self-reliance after bad period/lockdown IIIIIII
GRATEFUL: rarely
Grateful for themselves, for being safe I

…: aim 3: support

During COVID
LACK OF SUPPORT: the most
Lack of IIIIII
SCHOOL: the 2nd most
School lacks/ don't want IIIIII **School as a point of access** II
CLOSE PEOPLE: often
Friends II **Self-help** I **Family** II **Lack of family** I
LACK OF LEGITIMITY: often
illness not seen as legitimate (esp awareness ass BAME YP+gender) III **Reluctant to reach out** II
FINANCIAL/TECHNICAL: sometimes
Financial I **Technical** I

Now Want
ACTIVITIES: frequent
Activities IIIII
MOTIVATION: sometimes
Healthy lifestyle I **Motivational support** I
MENTAL HEALTH: most often
Mental health IIIIII **Intense relief when getting support** I
EDUCATION: often
Educational I **School support** II
YOUTH CLUBS: often
Youth club III
ARTS: frequent
Arts IIII
TO BE HEARD: 2nd most
Listened to IIIII
TO LEARN: sometimes
Learning about new issues II
SPORTS: often
Social sports IIII

WORK EXPERIENCE: rarely
    Work experience I

Reaching out I
LACK OF: sometimes
    Lack of adequate support (safe, accessible) II
SCHOOL AS A BARRIER: sometimes
    School as a barrier II

Appendix D

Initial Thematic Tables

Superordinate theme 1: Impact on mental health

A. Loneliness

Lack of social interactions

P103: 'lockdown obviously had a like really big impact on my mental health like it made me feel way more alone that like I actually was cause I couldn't go outside obviously.'

P103: 'I enjoyed the alone time the most but then it's also what like triggered me the most. I just kept I felt so trapped (…) And isolated but I also felt isolated and also felt like alone and unable to be myself in my own space.'

P104: 'But in that moment you're just like…I just want to be alone and it doesn't help it's like you're scurrying into a little hole (…) So I feel like also not having…people to talk to cause you can have like a million people around you and still feel alone. So I felt like in that moment I felt so alone and...tired and mentally exhausted and then school, and then.. not seeing friends and family members and the death it all just- (…) I feel like a lot of people different ages went through that same experience with like it just starts affecting you.'

P106: 'I felt actually quite sad (…) and angry because I'm at home. And like I just feel too much like energy in the house and you're too hot and you can't do this you can't do that and like you can't go out and that's like what frustrates me'

Self-esteem

P103: 'I had my brothers and my mum obviously but like they don't understand obviously, and um like I just really need someone to tell me like you're good enough (…) and like that's all like that's all anyone needs to hear, they just want validation (…) I just want to feel appreciated and like-like I just want to feel stuff like that.'

P103: 'There was a lot of challenges with like my mental health and like my self-esteem and like I- I still don't but like, I never believed in myself like at all, like at all/ (…) before I had no faith in myself like I used to believe that I couldn't do anything, I just like feel like I was never going to be good enough'

B. Mental disorders

Anxiety, Depression and Disordered eating

P104: 'but as well as mentally it really deteriorated like all youth, people my age like I realised that everybody was finding out they had depression, anxiety, it was really like draining on staying put, some people had toxic family members they had to stay home it was really like..it was hard.'

P104: 'I realised a lot of things which kind of drove me into a.. I would say quite a depression in the sense of like I wouldn't.. I would just staying in my room the whole time, I wouldn't leave it, stay on my phone but then log off every social media app possible to kind of just block out everything'

P103: 'I lost a lot of weight after the pandemic not in the best way. I didn't really eat, um but like (…)I never really used to- used to give myself like the attention I deserve, um like um and do you know with COVID it made it very very bad like my mental health was very bad, I was down there it was so bad. (…) and it was so such a heavy weight on my heart, it was like it was really hard for me and COVID made it all worse like trapped in my room seeing the same four walls everyday (…) so I was losing it, I was losing it.'

P100: 'Mentally, it affected me both like oh am I going to get COVID next, like got some sort of anxiety maybe with me.'

P102: 'Umm I've been sad, depressed…It was hard but I had to do it'

Trauma

P014: 'Like it just caused a lot of like thinking and..kind of just a lot of like reevaluating life and everything especially being in lockdown in your house and people passing away drastically like every day the numbers were increasing like I felt (…) there was times you really have to stop and think like this type of like disease this thing is wiping everybody out (…) anybody could have died from it.'

P104: 'This is really happening and nobody was grasping with the fact that people are really dying, (…) like it was sad that so many people

P104: ‘I realised that I had a lot of mental health problems within myself, and I wasn't not really happy, um not very confident, quite insecure’

Lack of trust in others/institutions
P104: ‘But I feel like everybody was more out for themselves and not really trying to really help each other (…) so it may have cause some type of division.. which kind of broke- I feel like the community was kind of broken (…) Like at some point, we weren't together, collective and stuff.’

Lack of motivation
P107: ‘And also um I feel like because I haven't been doing a lot I've lost the motivation to actually do things.’
P107: ‘Um mentally, I think yeah as I said before my motivation has taken like a big decline and also obviously I feel like bad habits during COVID there's not really much that you can do.’
P110: ‘A lot, uh yeah like laziness, um can't get out of bed as much, um yeah just a lot of that.’

couldn't handle COVID they took their lives like it was..it was absolutely devastating really, so yeah it was just so sad.’

C. Positive impact on mental health

New connections
P101: ‘I met lots of new people and got to interact with some people during lockdown so that was good,’
P104: ‘(People could) talk to each other, sit down and really think like what is going on in the world.’
P105: ‘but like we have gotten closer and we like text my friends during in the middle of class when I was in lockdown and we’d sometimes have video calls and stuff.’
P107: ‘Yeah um I've got a lot closer with a lot of my friends’.

New mindful activities
P100: ‘Um, I was able to try things that I wouldn’t try on a day to day basis (…) like going for walks and stuff and like doing drawings.’
P101:‘I started to find new hobbies like poetry, um and we started going to this poetry night which was like good’
P104: ‘during summer like obviously summer it gets dark really late um I used to go for like walks around my neighbourhood and obviously I had like no phone nothing so like I would just like I just used to reflect and it was such a like a peaceful time like after I came back home I thought like..I just felt like free. I felt like there was no **** nothing.’

Solitude
P100: ‘(I enjoyed) having me time. Like being able to do like stuff on my own.’
P107: ‘Um, things I enjoyed the most I’d probably say I got a lot of time to myself.’

Superordinate theme 2: Changes in mental health since the start of lockdown

A. Post-lockdown high

Freedom
P103: ‘But like at the end of lockdown when everything like um the restrictions started lifting I felt like I was finally getting life back.’

B. Gradual mental health coping

Isolation and overthinking
P102: ‘I had to do it because if I didn’t then how am I supposed to go in life so I literally just looked at the present and just focusing on what I need to do to get through my life.’

P106: 'going out in groups without having to get COVID tested themselves before they left the house.'<br>P107: 'Cause now that I've finally got the freedom to be out of my house (…) I was out of my house all the time.'<br><br>*Social closeness*<br>P104: 'Seeing my friends for the first time. Like I can't fake that that was like the best feeling cause it's like all these months I haven't seen you and it's like oh my god.'<br>P104: 'And I would see no one, it was so.. everyone was scared for their lives, like nobody was outside..it was like (sigh) so I guess when I started seeing the community, everybody kind of coming, trying to make this new normal, I guess that was really nice'<br>P107: 'when the lockdown when the restrictions first eased like in the summer of 2020. (…) Yeah I feel like then I got a lot closer with a lot of people.'<br>P109:'Back then I wasn't as social (…) Cause I found a really good series of books and I just kept reading it.(…) It's not that I couldn't be social it's just that I wasn't bothered to, but now I'm like talking a bit more, still reading though.'<br>P110: 'Good, (friends and family relationships) got even stronger since we could interact again.'	P103: 'Cause it was a very long time just self-reflecting and like, I liked it- it was very therapeutic but at the same time I hated it I felt like I was being cooped up and like trapped/'<br>P103: 'started overthinking like I started to like really, evaluate my personality and like who I am as a person (…) and like my mental health as well cause I've never really thought about mental health at all, ok I'm not ok but there's nothing I can do'<br>P103: 'before the pandemic I never really like cared about myself (…)I think that was mainly because there was all this stuff like all this stuff that was just built up and I never had a chance to release it cause I was always (…) so I never had to think about it.'<br><br>*Better coping*<br>P102: 'I have ADHD (…) So I know what time I'll be coming home and (my mum) will have checked on me cause I'll be doing silly stuff so, like stuff like that has changed me and normally it would ne- I would never do that so I'm happy that I've done that. (…)I'm organised better. (…) So that's like COVID, I think COVID actually helped me do all of this stuff, so yeah'<br>P103: 'those little things that I did like I used to go on walks or I just used to like I used to write down my thoughts a lot like it was just like random bits of what I'm feeling throughout the day and like those kind of help you feel better.'<br>P103: 'when I realised it's like there so many different things that I can do to help myself and I just want to find all the right um outlets to project any um anger or aggression any uh sad feelings that I had without causing violence.'<br><br>*Better mental health*<br>P102: 'Uh, in my opinion I've known I've been depressed I know I've been all down and whatever, but I think if I didn't have that stage in my life I would not be what who I am right now'<br>P102: 'I think I've changed for the better cause I used to be like a naughty kid. (…) So now I've like realised like I need to learn, revise,.. no friends like everything changed and I kind of like it because I've never been like this before so'<br>P102: 'So like, a few years ago I was crazy, I was hanging around with the wrong crowd, and now I've realised like, I've realised I've grown and I have to show my little sister that I can't be

	like who I am right now, so I had to change my personality just for her (…) cause if it wasn't for her then I would still be behaving bad and yeah'<br>P103: 'And um like I think it strengthened my relationships but it also strengthened the relationship that I had with myself which is like one of the most important ones, so yeah'<br>P103: 'I think I'm good now, I think if you'd asked- I think if you'd done this interview like a couple months ago (…) I was so stuck, I would have been like I need someone to just talk to.'

Superordinate theme 3: Support obtained during pandemic and needs now	
**A. Support**<br><br>*Lack of support*<br>P100: 'Honestly little to none, support yeah'<br>P103:'I wish I would've know that there's people that I can talk to, or like there's things that I can do to like lose weight healthier rather than just starving myself or I wish there was someone to tell you that like you're enough'<br>P104: 'people I know have developed eating disorders, people that maybe had trauma or shock from a lost one like .. it all stems mentally and I feel like if they would've had mental help, when you neglect your mental health you neglect your physical health, it all connects.'<br>P110: 'I wouldn't say there was support, it was just really independent.'<br><br>*Neglect*<br>P102: 'I would actually listen to people because clearly the government doesn't listen to no one he just he does everything what he thinks and I don't I don't trust that, and I don't believe in that so I will, if I was the government, of course I would change everything, I would listen to everybody, even the poor, change a lot of things. (…) I didn't care about that (before COVID) but now I do.'<br>P102: 'I know I have issues (…) It's just like, I need more support with that um. (…) No one is listening. So I have to shout it out loud so everyone can listen, so yeah.'<br>P104: 'like in this generation and age there are so many misconceptions about teenagers, especially me as a black female teenager, there's many misconceptions about…being…you know who you are or what you look like'<br><br>*Structural*	**B. Needs**<br><br>*Psychotherapy*<br>P103: 'if that person is trained to deal with like mental health issues it's way easier to like open up to them and like when I got a counsellor and a mentor like I finally felt like I could talk to someone else about it.'<br>P104: 'I feel like sometimes psychologically, just like someone to understand and hear me (…) it's hard to kind of like, to speak to somebody without your mind being like oh maybe this person thinks I'm crazy, it would be nice to be finally heard by someone and to talk about your feelings and interests without someone, or your feelings that you felt before, without someone judging you. Because sometimes you just, you just need an ear. You just need someone to listen. I feel like that would really help actually. And it would've helped I think back then if I'd had that I think it would have been way easier to talk about.'<br>P104: 'and (the therapist) just said what you described does sound like it, and I was like, in that moment like a tear fell on my face because I was like really like it's been confirmed, like maybe he didn't say yeah you have depression but he basically said you're on the way to it,'<br>P106: ' (I would need) Like people speaking to me.'<br><br>*Relational*<br>P103: 'I still have support but it's like from my friends.'<br>P105: 'I would have like hugs from my friends. (…)I felt like it's a way of comforting myself and comforting other people when it's like hardships and stuff. (…) It just like helps a lot. Because even the simplest thing, like love can do a lot.'

P103: 'it's like sometimes we don't have the best relationship with teachers and like because of the past I've been at that school for what like 4 years. (…) there's like background there's a past with some of the teachers and it's not really the best to confide in them'.<br>P100: 'just like in school, it's very much like,…once in a blue moon ask us if we're alright and that's it.<br>P106: 'they don't help they only sit in my class and do nothing when I ask for help then sometimes they listen.'	P104: 'Um to be honest I actually didn't (reach out) (…) I feel like I made that little depressive state my normal. (…) So I thought- I also didn't reach out I didn't think there was anything wrong with it (…) and it's kind of like, asking for help I'm not really a person that does that. (…)I'm not really a person that goes out for support so that denial was kind of like a comfort place like no you don't need anybody you're fine like you don't need to talk to anybody you're ok, like people who talk to people you don't you don't need that you're not on that level you're ok so I kind of talked myself into not getting help, not speaking to anybody, which caused me to feel really alone.'<br><br>*Need for daily life*<br>P107: 'I would say maybe more opportunities available. So stuff like sports. Even something like work experience. Sort of that yeah I feel like that would help a lot cause it would help like ease back into things.'<br>P107: 'I've got very used to the connections I have now cause that's who I rely on for a while, so making new friendships, relationships, has become a lot harder.'<br>P100: 'About how to maintain a healthy lifestyle even though…circumstances can change like this (fingers snapping).'<br>P105: 'But I like to talking about things like that and painting and drawing..it's really nice.It makes me happy. (…) I like doing photography without being restricted.'<br>P106: 'I go to (a youthclub) and do some art and yeah.

Appendix E
Final Thematic Tables

Superordinate theme 1: Impact of the Covid-19 pandemic on mental health

A. Individual

Self-esteem

P103: 'I had my brothers and my mum obviously but like they don't understand obviously, and um like I just really need someone to tell me like you're good enough (…) and like that's all like that's all anyone needs to hear, they just want validation (…) I just want to feel appreciated and like-like I just want to feel stuff like that.'

P103: 'There was a lot of challenges with like my mental health and like my self-esteem and like I- I still don't but like, I never believed in myself like at all, like at all/ (…) I just like feel like I was never going to be good enough'

P104: 'I realised that I had a lot of mental health problems within myself, and I wasn't not really happy, um not very confident, quite insecure'

Lack of motivation

P107: 'I feel like because I haven't been doing a lot I've lost the motivation to actually do things.'

P107: 'Um mentally, I think yeah as I said before my motivation has taken like a big decline and also obviously I feel like bad habits during COVID there's not really much that you can do.'

P110: 'A lot, uh yeah like laziness, um can't get out of bed as much.'

B. Symptoms of mental illness

Anxiety, Depression and Disordered Eating

P100: 'Mentally, it affected me both like oh am I going to get COVID next, like got some sort of anxiety maybe with me.'

P102: 'Umm I've been sad, depressed…It was hard but I had to do it'

P103: 'I lost a lot of weight after the pandemic not in the best way. I didn't really eat (…)I never really used to give myself like the attention I deserve and you know with COVID it made it very very bad like my mental health was very bad, I was down there it was so bad. (…) **and it was so such a heavy weight on my heart, it was like it was really hard for me and**

C. Relational

Isolation and individualisation

P103: '(lockdown had a) big impact on my mental health like it made me feel way more alone that like I actually was cause I couldn't go outside obviously. (…) I also felt isolated and also felt like alone and unable to be myself in my own space.'

P104: 'cause you can have like a million people around you and still feel alone. So I felt like in that moment I felt so alone and...tired and mentally exhausted (…) But I feel like everybody was more out for themselves and not really trying to really help each other (…) so it may have cause some type of division.. **I feel like the community was kind of broken'**

P106: 'I felt actually quite sad (…) and angry because I'm at home. And like I just feel too much like energy in the house and you're too hot and you can't do this you can't do that and like you can't go out and that's like what frustrates me'

Positive solitude

P100: '(I enjoyed) having me time. Like being able to do like stuff on my own.'

P107: 'Um, things I enjoyed the most I'd probably say I got a lot of time to myself.'

P104: 'during summer like obviously summer it gets dark really late um **I used to go for like walks around my neighbourhood** and obviously I had like no phone nothing so like I would just **like I just used to reflect and it was such a like a peaceful time like after I came back home I thought like..I just felt like free. I felt like there was no **** nothing.'**

New connections

P101: 'I met lots of new people and got to interact with some people during lockdown'

P104: '(People could) talk to each other, sit down and really think like what is going on in the world.'

P104: 'I definitely lost friendships and gained friendships.Like I found true friends and I also lost people that I thought were good friends to me, but it just showed that they weren't good friends at all.'

**COVID made it all worse (…) so I was losing it, I was losing it.'**<br>**P104**: '**I realised a lot of things which kind of drove me into a.. I would say quite a depression in the sense of like I would just staying in my room the whole time, I wouldn't leave it,** stay on my phone but then log off every social media app possible to kind of just block out everything'<br><br>*Trauma*<br>P014: 'people passing away drastically like every day the numbers were increasing like I felt (…) there was times you really have to stop and think like this type of like disease this thing is wiping everybody out (…) anybody could have died from it.'<br>**P104: '**This is really happening and **nobody was grasping with the fact that people are really dying, (…) like it was sad that so many people couldn't handle COVID they took their lives like it was..it was absolutely devastating.'**	P105: 'we have gotten closer and we like text my friends during in the middle of class when I was in lockdown and we'd sometimes have video calls.'

Superordinate theme 2: Changes in mental health since the end of lockdown	
A. **Autonomous adaptive coping skills**<br><br>*Overthinking*<br>**P102: 'I had to do it because if I didn't then how am I supposed to go in life so I literally just looked at the present and just focusing on what I need to do to get through my life.'**<br>**P103: '**started **overthinking like I started to like really, evaluate my personality and like who I am as a person** (…) and like my mental health as well cause I've never really thought about mental health at all, ok I'm not ok but there's nothing I can do'<br>P103: 'before the pandemic I never really like cared about myself (…)I think that was mainly because there was all this stuff like that was just built up and I never had a chance to release it cause I was always out so I never had to think about it, and COVID made it all worse'<br><br>*Better coping*<br>P102: 'I have ADHD (…) so I've set timers to go out for 5 minutes, take my walks. I tell my mum, she has a timer on her phone and I know what time I'll be coming home and (my mum)	B. **Lifted restrictions**<br><br>*Freedom*<br>**P103: 'At the end of lockdown when everything like um the restrictions started lifting I felt like I was finally getting life back.'**<br>**P106: 'going out in groups without having to get COVID tested themselves before they left the house'[1].**<br>P107: 'Cause now that I've finally got the freedom to be out of my house (…) I was out of my house all the time.'<br>**P107: 'I took things for granted because obviously something as simple as being outside and going to the shops it's all changed.'**<br><br>*Social bonds*<br>**P104: 'Seeing my friends for the first time. Like I can't fake that that was like the best feeling cause it's like all these months I haven't seen you and it's like oh my god.'**<br>**P107: 'when the restrictions first eased like in the summer of 2020. (…) Yeah I feel like then I got a lot closer with a lot of people.'**

1

will have checked on me cause I'll be doing silly stuff so, like stuff like that has changed me and normally it would ne- I would never do that so I'm happy that I've done that. (…)I'm organised better. (…) So that's like COVID, I think COVID actually helped me do all of this stuff, so yeah'<br>**P103: 'those little things that I did like I used to go on walks or I just used to like I used to write down my thoughts a lot like it was just like random bits of what I'm feeling throughout the day and like those kind of help you feel better.'**<br>**P103: 'when I realised it's like there so many different things that I can do to help myself and I just want to find all the right um outlets to project any um anger or aggression any uh sad feelings that I had without causing violence.'**<br><br>*Personal growth*<br>**P102**: 'I've been depressed I know I've been all down and whatever, **but I think if I didn't have that stage in my life I would not be what who I am right now** (…) I think I've changed for the better cause I used to be like a naughty kid. (…) So now I've like realised like I need to learn, revise,.. like everything changed and I kind of like it because I've never been like this before.'<br>P102: 'So like, a few years ago I was crazy, I was hanging around with the wrong crowd, and now I've realised like, I've realised I've grown and I have to show my little sister that I can't be like who I am right now, so I had to change my personality just for her (…) cause if it wasn't for her then I would still be behaving bad'<br>**P103**: 'And um like I think it strengthened my relationships but **it also strengthened the relationship that I had with myself which is like one of the most important one'**<br>P103: 'I think I'm good now, I think if you'd asked- I think if you'd done this interview like a couple months ago (…) I was so stuck, I would have been like I need someone to just talk to.'<br>**P109:'Back then I wasn't as social (…) Cause I found a really good series of books and I just kept reading it.(…) It's not that I couldn't be social it's just that I wasn't bothered to, but now I'm like talking a bit more, still reading though.'**	P110: 'Good, (friends and family relationships) got even stronger since we could interact again.'

Superordinate theme 3: Limited support obtained during the Covid-19 pandemic and current needs

A. Support

Lack of support

P100: 'Honestly little to none, support yeah'

P103:'I wish I would've know that there's people that I can talk to, or like there's things that I can do to like lose weight healthier rather than just starving myself or I wish there was someone to tell you that like you're enough'

P104: 'people I know have developed eating disorders, people that maybe had trauma or shock from a lost one like .. it all stems mentally and I feel like if they would've had mental help, when you neglect your mental health you neglect your physical health, it all connects.'

P110: 'I wouldn't say there was support, it was just really independent.'

Neglect

P102: 'I would actually listen to people because **clearly the government doesn't listen to no one he just he does everything what he thinks and I don't I don't trust that**, and I don't believe in that so I will, if I was the government, of course I would change everything, I would listen to everybody, even the poor, change a lot of things. (…) I didn't care about that (before COVID) but now I do.'

P102: 'I know I have issues (…) It's just like**, I need more support with that. (…) No one is listening. So I have to shout it out loud so everyone can listen,** so yeah.'

P104: '**like in this generation and age there are so many misconceptions about teenagers, especially me as a black female teenager, there's many misconceptions about…being…you know who you are or what you look like'**

Structural

P103: 'it's like sometimes we don't have the best relationship with teachers and like because of the past I've been at that school for what like 4 years. (…) there's like background there's a past with some of the teachers and it's not really the best to confide in them'.

P100: 'in school, it's very much like,…once in a blue moon ask us if we're alright and that's it.'

B. Needs

Psychological

P103: 'if that person is trained to deal with like mental health issues it's way easier to like open up to them and like when I got a counsellor and a mentor like I finally felt like I could talk to someone else about it.'

P104: 'I feel like sometimes psychologically, just like someone to understand and hear me (…) it's hard to kind of like, to speak to somebody without your mind being like oh maybe this person thinks I'm crazy, it would be nice to be finally heard by someone and to talk about your feelings and interests without someone, or your feelings that you felt before, without someone judging you. Because **sometimes you just, you just need an ear. You just need someone to listen. I feel like that would really help actually. And it would've helped I think back then if I'd had that I think it would have been way easier to talk about.'**

P104: 'and (the therapist) just said what you described does sound like (depression), and I was like, in that moment like a tear fell on my face because I was like really like it's been confirmed, like maybe he didn't say yeah you have depression but he basically said you're on the way to it,'

P106: ' (I would need) Like people speaking to me.'

Relational

P103: 'I still have support but it's like from my friends.'

P105: 'I would have like **hugs from my friends. I feel like it's a way of comforting myself and comforting other people when it's like hardships and stuff, it just like helps a lot because even the simplest thing, like love can do a lot.'**

P104: 'Um to be honest I actually didn't (reach out) (…) I feel like I made that little depressive state my normal. (…) So I thought- I also didn't reach out I didn't think there was anything wrong with it (…) I'm not really a person that goes out for support so that denial was kind of like a comfort place like no you don't need anybody you're fine like you don't need to talk to anybody you're ok, like people who talk to people you don't you don't need that you're not on that level

**P106: 'they don't help they only sit in my class and do nothing when I ask for help then sometimes they listen.'**	you're ok so I kind of talked myself into not getting help, not speaking to anybody, which caused me to feel really alone.'<br><br>*Practical*<br>**P107:** 'I would say maybe **more opportunities available. So stuff like sports. Even something like work experience. Sort of that yeah I feel like that would help a lot cause it would help like ease back into things (…) making new friendships, relationships, has become a lot harder.'**<br>**P100**: 'About **how to maintain a healthy lifestyle even though…circumstances can change like this (snaps fingers).'**<br>P105: 'But I like to talking about things like that and painting and drawing..it's really nice.It makes me happy. (…) I like doing photography without being restricted.'<br>P106: 'I go to (a youthclub) and do some art and yeah.'
