# Supplementary material for: Impact of the COVID-19 pandemic on young people from black and mixed ethnic groups’ mental health in West London: a qualitative study
Source: BMJ Open. 2023 May 5;13(5):e071903. doi: 10.1136/bmjopen-2023-071903 (PMC10163329; doi:10.1136/bmjopen-2023-071903)
Supplement: Author's manuscript [file bmjopen-2023-071903.draft_revisions.pdf]

BMJ Open

BMJ Open is committed to open peer review. As part of this commitment we make the peer review history of every article we publish publicly available.

When an article is published we post the peer reviewers' comments and the authors' responses online. We also post the versions of the paper that were used during peer review. These are the versions that the peer review comments apply to.

The versions of the paper that follow are the versions that were submitted during the peer review process. They are not the versions of record or the final published versions. They should not be cited or distributed as the published version of this manuscript.

BMJ Open is an open access journal and the full, final, typeset and author-corrected version of record of the manuscript is available on our site with no access controls, subscription charges or pay-per-view fees (http://bmjopen.bmj.com).

If you have any questions on BMJ Open's open peer review process please email info.bmjopen@bmj.com

The impact of the Covid-19 pandemic on young people from black and mixed-ethnic groups' mental health: A qualitative study.

Journal:	*BMJ Open*
Manuscript ID	bmjopen-2023-071903
Article Type:	Original research
Date Submitted by the Author:	27-Jan-2023
Complete List of Authors:	Lenoir, Romane; University College London, Psychology & Human Development<br>Wong, Keri Ka-Yee ; University College London, Psychology & Human Development
Keywords:	COVID-19, MENTAL HEALTH, QUALITATIVE RESEARCH

SCHOLARONE™
Manuscripts

I, the Submitting Author has the right to grant and does grant on behalf of all authors of the Work (as defined in the below author licence), an exclusive licence and/or a non-exclusive licence for contributions from authors who are: i) UK Crown employees; ii) where BMJ has agreed a CC-BY licence shall apply, and/or iii) in accordance with the terms applicable for US Federal Government officers or employees acting as part of their official duties; on a worldwide, perpetual, irrevocable, royalty-free basis to BMJ Publishing Group Ltd ("BMJ") its licensees and where the relevant Journal is co-owned by BMJ to the co-owners of the Journal, to publish the Work in this journal and any other BMJ products and to exploit all rights, as set out in our licence.

The Submitting Author accepts and understands that any supply made under these terms is made by BMJ to the Submitting Author unless you are acting as an employee on behalf of your employer or a postgraduate student of an affiliated institution which is paying any applicable article publishing charge ("APC") for Open Access articles. Where the Submitting Author wishes to make the Work available on an Open Access basis (and intends to pay the relevant APC), the terms of reuse of such Open Access shall be governed by a Creative Commons licence – details of these licences and which Creative Commons licence will apply to this Work are set out in our licence referred to above.

Other than as permitted in any relevant BMJ Author's Self Archiving Policies, I confirm this Work has not been accepted for publication elsewhere, is not being considered for publication elsewhere and does not duplicate material already published. I confirm all authors consent to publication of this Work and authorise the granting of this licence.

For peer review only

The impact of the Covid-19 pandemic on young people from black and mixed-ethnic groups' mental health: A qualitative study.

Author: Romane Lenoir, BSc Psychology with Education Alumni, Department of Psychology and Human Development, University College London, London, United-Kingdom

Co-author: Dr. Keri Ka-Yee Wong, Assistant Professor in Psychology, Department of Psychology and Human Development, University College London, London, United-Kingdom

Corresponding author: Dr. Keri Ka-Yee Wong, Department of Psychology and Human Development, 25 Woburn Square, London WC1H 0AA, keri.wong@ucl.ac.uk, Tel: (+44) 78997-98350

Key words: Covid-19; Mental health; Qualitative Research

Word count: 4045 words

For peer review only

Abstract

Objectives

The Covid-19 pandemic has disproportionately impacted vulnerable groups' physical and mental health, especially young people and minority ethnic groups, yet little is known about how this is taking place and what support they would like. To address this gap, this qualitative study aims to uncover the effect of the Covid-19 outbreak on young people with ethnic minority backgrounds' mental health, how this changed since the end of lockdown and what support they need to cope with these issues.

Setting and Participants

Ten 20-minute in-person semi-structured interviews were conducted with young people aged 12 to 17 years old from black and mixed-ethnic groups who regularly attend a community centre in West London.

Results

Through Interpretative Phenomenological Analysis, results indicated that the participants' mental health was negatively impacted by the Covid-19 pandemic, with feelings of loneliness being the most common experience. However, positive effects were concurrently observed including improved well-being and better coping strategies post-lockdown, which is a testament to the young people's resilience. That said, it is clear that young people from minority ethnic backgrounds lacked support during the Covid-19 pandemic and would now need psychological, practical and relational assistance to cope with these challenges.

Conclusions

Whilst future studies would benefit from a larger ethnically-diverse sample, this is a start. Study findings have the potential to inform future government policies around mental health support

For peer review only - http://bmjopen.bmj.com/site/about/guidelines.xhtml

and access for young people from ethnic minorities, notably prioritising support for grassroots initiatives during times of crisis.

Strengths and limitations

- This qualitative interview study during Covid-19 gives voice to the experiences of young people from black and mixed-ethnic backgrounds in the UK
- The in-person quality of the interviews helped build rapport between the researcher and the young people and sharing of sensitive issues around mental health access and support, increasing the results' validity
- This is a convenient sample, with girls and those aged 15 years and above being disproportionately represented in our data as they provided most of the answers.
- The small sample size and lack of ethnic diversity limits the generalisability of the study to individuals from other ethnic minority groups.

For peer review only

The severe acute respiratory syndrome SARS-CoV-2 (Covid-19) coronavirus, infecting over 600 million people and claiming more than six million lives and counting, has triggered unprecedented economic and social disruptions worldwide [1, 2]. Whilst everyone has been impacted, certain groups have been disproportionately affected [3], including individuals from ethnic minority groups [4]. Mortality rates in the United Kingdom (UK) between March and April 2020 for instance, were two and three times higher for individuals from minority ethnic groups than for ethnically white groups [5]. In April 2021, the UK Intensive Care National Audit and Research Centre reported that ethnic minorities represented 34% of the patients in intensive care units despite only constituting 11% of the UK population [6]. These alarming trends of physical vulnerability for individuals from ethnic minorities during Covid-19 begs the question of why this is the case, and what support might look like for this group.

Various explanations for the disproportionate impact of Covid-19 on ethnic minority groups have been raised. A biological explanation would suggest that ethnic minority groups have a higher risk of developing stress-related physiological responses and comorbidities [7, 8]. An ecological explanation of the dynamic interplay between an individual and their environment [9, 10] alternatively suggests that situational risk factors such as multi-generational living conditions and environmental adversity - most notably associated with

For peer review only - http://bmjopen.bmj.com/site/about/guidelines.xhtml

individuals from minority ethnic groups' living conditions - may increase ethnic minority groups' vulnerability to Covid-19 [11]. Ethnic minority groups are overrepresented in socially disadvantaged neighbourhoods, with white individuals only representing 8,7% of the population in the 10% most income-deprived neighbourhoods in the UK in 2016 [12]. These are characterised by a limited capacity to socially distance, resulting in higher Covid-19 infection and death rates [13]. Ethnic minority individuals also occupy more key-worker positions, as they constituted over 20% of the NHS workforce whilst representing under 15% of the working-age population in the UK in 2019 [14]. This overall disadvantage in the labour market exposes them to greater risk during the Covid-19 pandemic [15]. Finally, it may also be the case that social networks being racially differentiated increases intra-community transmission for ethnic minority groups [16]. The Covid-19 pandemic thus not only exacerbated pre-existing health inequalities for ethnic minority groups in the UK, but deepened socio-economic ones, the impact of which must be investigated [17].

Studies of the effect of the Covid-19 pandemic on ethnic minority groups' day-to-day life stressors have been particularly informative. The Mind charity surveyed UK adults' experiences during the pandemic and found that individuals from minority ethnic groups experienced higher levels of everyday distress [18]. 30% of adults from ethnic minorities reported that their mental health was affected by housing issues, compared to 23% of whites.

For peer review only - http://bmjopen.bmj.com/site/about/guidelines.xhtml

Similar rates were reported for troubles with employment (61% against 51% for white individuals), finances (52% against 45%) and physical health support (39% versus 29%) [18]. However, ethnic minorities notably represented less than 5% of the study's sample. Additionally, minority ethnic individuals in the UK were more exposed to food insecurity during Covid, and had a higher reliance on food banks than their white counterparts [19]. Finally, long-term inequalities in socioeconomic opportunities and a history of mistrust towards governing entities have cultivated feelings of alienation amongst minority ethnic groups [20, 21]. The Covid-19 pandemic deepened this rift, reinforcing feelings of social isolation [16, 22].

Young people from ethnic minorities have also suffered during Covid-19, being deprived of structures essential to their socio-emotional development, which caused increased psychological distress [23-25]. Yet, young people have also demonstrated considerable resilience [26, 27]. Documenting these experiences is crucial as young peoples' voices are too often missing from academic research on Covid-19 and mental health due to difficulties in recruitment and access. Their scarcity makes developing support mechanisms for young people rather difficult. Also, as a majority of studies are quantitative in nature [28, 29] which, albeit informative, does not create a space for young people to voice their needs and allow them to

For peer review only

feel heard. Not least, young people from minority ethnic groups are at heightened risk of experiencing distress during the Covid-19 pandemic and therefore deserving to be actively involved in and listened to in this research.

Existing quantitative studies on the mental health of individuals from minority ethnic backgrounds are limited in that they very rarely examine the underlying nature of the relationships. To date, only one qualitative study has focused on the impact of Covid-19 on minority ethnic individuals to elucidate 'why' certain relationships exist. Mahmood et al. [30] interviewed 19 ethnic minority community leaders between October and November 2020 about the Covid-19 pandemic's impact on their communities. Participants spoke about the financial, social and physical consequences of the pandemic, the latter being subjectively justified by historical and structural disparities. Results indicated that mental health was a fundamental concern, as the participants invoked their group's usual tight-knit nature to illustrate the extent of lockdown's impact on their well-being [30]. Other qualitative studies on young people's mental health during the pandemic, although not specifically focusing on ethnic minorities, nonetheless also note worrying patterns [31, 32]. The scarcity of literature concentrating on first-hand experiences of minority ethnic groups throughout the Covid-19 pandemic highlights

For peer review only

the need for more qualitative studies to truly understand how this pandemic has impacted this group.

With the pandemic still largely unfolding, we must identify what the barriers to mental health support might be and how can we best support young people now rather than later. First, recognising that young people's struggles are legitimate and encouraging them to seek support is essential [18]. Second, the structural and interpersonal obstacles young people from ethnic minorities face when seeking mental health support are a key factor driving low incidences of access to support [33]. Within the Mind survey, half of the young people revealed that difficulties in accessing support made their mental health worse [18]. Lower English proficiency has also been associated with reduced use of mental health resources, with a significantly steeper effect within minority ethnic groups [34]. The low intelligibility of online information surrounding Covid-19 in the UK revealed a striking lack of appropriate education materials available to these groups [35]. Further isolating young people, these obstacles exacerbate their distrust, and create a reluctance and stigma to seeking mental health support [30]. The reality is that ethnic minority young people's mental health has not only been worsening since the start of the Covid-19 pandemic but is also poorly assisted [36, 37].

The current qualitative interview study aims to identify the impact of the Covid-19 pandemic on young people from minority ethnic groups and the types of support they might need. The Covid-19 pandemic is hypothesised to have negatively impacted the mental health of young people from black and mixed-ethnic groups, which has continued to decline after

For peer review only

lockdown measures eased and finally, that support is needed, the nature of which will be uncovered through the study. By giving voice to communities less heard from, we hope to inspire larger-scale research and develop tailored mental health support for black and mixed ethnic minority communities.

Methods

Participants and Ethics

The ten participants were recruited via convenience sampling from a community centre in West London. The current pre-registered CopeWell Study (https://osf.io/jcak7/), funded by UKRI Research England , aimed to understand the impact of the Covid-19 pandemic on young people's mental and physical health and to co-create appropriate life-skill workshops to enable young people from black and mixed-ethnic backgrounds to better cope with the pandemic [38]. The present study focuses on the initial interviews on the impact and challenges of Covid-19 on young people's lives. Ethical approval was granted by the UCL Institute of Education Ethics Committee (REC 1558) on 29 October 2021. A pilot study was conducted for the interview schedule, confirming that the questions' content and order were cohesive (Appendix A). The prods' usefulness in allowing the participants to elaborate was assessed, familiarising the researcher with their navigation. Parental informed consent was received before the study

For peer review only

commenced and participant consent was obtained at the time of the interview. Prior to this, participants were reminded of their right to withdraw or omit to answer at any point. Confidentiality was ensured and potential questions were answered. Next, participants took part in a 15-minute one-on-one semi-structured interview (*M* = 14:06 min) with 14 open-ended questions. They were thanked and debriefed post-interview and received a £30 gift voucher as an honorarium. We built rapport with the youth workers and young people before conducting the interviews at the community centre (a familiar and safe environment). Covid-19 safety measures (e.g. masking, distancing, well-ventilated space) were applied where necessary. The participants' answers were recorded via audio software to maximise accuracy and were subsequently transcribed by R.L..

Materials

The initial pilot interview schedule contained 14 open-ended questions, with prods guiding the discussion according to the participants' reactivity and answers, and was designed by K.W.. It was subsequently amended in consultation with R.L. to form the final version (Appendix B). Questions progressed from items about the Covid-19 pandemic and its impact on the participants' mental health, to potential changes in these experiences. This was followed by the kind of support they received and needed to cope with these struggles.

For peer review only - http://bmjopen.bmj.com/site/about/guidelines.xhtml

Data analysis

The analysis was conducted following Smith's Interpretative Phenomenological Analysis (IPA) [39]. IPA is relevant for this study as it draws focus on the young people's points of view and the meanings they ascribe to their situation. The explicitly idiographic nature of this analytical method allowed for the participants' lived experiences to be thoroughly probed into [40]. Smith and Shinebourne's six steps analysis for IPA was followed [41], resulting in the delineation of three *superordinate themes*, each containing *themes* and *subthemes*. Through an iterative process of reading and re-reading the transcripts, the researcher (R.L.) made initial annotations and colour-coded quotes according to the study aims they pertained to. The most frequent and significant patterns were then clustered together (Appendix C) and agreed with K.W. through discussions. The thematic relationship was summarised in an initial table with subthemes and sample quotes (Appendix D). IPA, as an iterative process, allowed the researcher to continuously revise themes and quotes, concomitantly ensuring the integrity of the participants' accounts was preserved [41]. Applying this not only preserved the interpretative role of the researcher in IPA, but buffered researcher bias risks [42, 43]. The final thematic relationship is presented in a table (Appendix E).

Patient and public involvement

For peer review only

For peer review only - http://bmjopen.bmj.com/site/about/guidelines.xhtml

Patients or the public were not involved in the design, or conduct, or reporting or dissemination plans of our research.

Results

A total of ten young people aged 12 to 17 participated in the study (M = 14.3, SD = 1.3 years, female = 70%). They self-identified as black or black African (n = 4), black or black Caribbean (n = 1), any other black background (n = 1), white and black African (n = 2) and white and black Caribbean (n = 1). One participant preferred not to say. Three superordinate themes emerged, namely overall declining patterns of mental health during the Covid-19 pandemic, improvements post-lockdown, and needs moving forward.

Superordinate theme one: Impact of the Covid-19 pandemic on mental health (table 1)

Individual

As a result of being required to stay indoors, 30% of the young people described suffering from decreased self-esteem during the Covid-19 pandemic. Underlying insecurities manifested themselves through self-doubt and low confidence. Additionally, 20% of the young people spoke about the monotony of life during lockdown causing them to lose their motivation and ambition.

For peer review only

Symptoms of mental illness

Participants' previous apprehensions about life and patterns of low mood were intensified by the Covid-19 pandemic, as 60% of them recounted symptoms of anxiety, depression and disordered eating. Lockdown freed up time for overthinking and the development of unhealthy habits of thoughts and behaviour. These were heightened by anxieties surrounding the illness itself for 20% of the participants, through exposure to a number of traumatic events such as suicides and climbing death rates.

Relational

The isolation provoked by lockdown shifted the participants' rapport to others, negatively impacting 90% of the young people's mental health. Loneliness and fears arising from the speed and risk of virus transmission fuelled tensions and individualisation trends amongst ethnic minority groups. However, solitude was also perceived as restorative, as 30% of the young people utilised this time to reflect and enjoy time on their own as a break from an otherwise hectic life. Interestingly, 50% of the participants also reported that the Covid-19 pandemic not only allowed them to reinforce current connections and friendships but to develop new ones.

Table 1: Impact of the Covid-19 pandemic on mental health

For peer review only

Theme	Subtheme	Illustrative quotes
Individual impact	Self-esteem	P103: "There was a lot of challenges with like my mental health and like my self-esteem and like I- I still don't but like, I never believed in myself like at all, like at all/ (…) I just like feel like I was never going to be good enough" (age 15)<br>P104: "I realised that I had a lot of mental health problems within myself, and I wasn't not really happy, um not very confident, quite insecure" (age 16)
	Lack of motivation	P107: "I feel like because I haven't been doing a lot I've lost the motivation to actually do things." (age 15)<br>P107: "Um mentally, I think yeah as I said before my motivation has taken like a big decline and also obviously I feel like bad habits during COVID there's not really much that you can do." (age 15)<br>P110: "A lot, uh yeah like laziness, um can't get out of bed as much." (age 15)
Symptoms of mental illness	Anxiety, Depression and Disordered Eating	P100: "Mentally, it affected me both like oh am I going to get COVID next, like got some sort of anxiety" (age 15)<br>P103: "I lost a lot of weight after the pandemic not in the best way. I didn't really eat (…)I never really used to give myself like the attention I deserve and you know with COVID it made it very very bad like my mental health was very bad, **(…)** and it was so such a heavy weight on my heart, it was like it was really hard for me and COVID made it all worse (…) so I was losing it, I was losing it." (age 15)<br>P104: **"**I realised a lot of things which kind of drove me into a.. I would say quite a depression in the sense of like I would just staying in my room the whole time, I wouldn't leave it" (age 16)
	Trauma	P104: "nobody was grasping with the fact that people are really dying, (…) like it was sad that so many people couldn't handle COVID they took their lives like it was..it was absolutely devastating." (age 16)
Relational	Isolation and individualisation	P103: "(lockdown had a) big impact on my mental health like it made me feel way more alone that like I actually was cause I couldn't go outside obviously. (…) I also felt isolated and also felt like alone and unable to be myself in my own space." (age 15)

For peer review only - http://bmjopen.bmj.com/site/about/guidelines.xhtml

		P104: "cause you can have like a million people around you and still feel alone. So I felt like in that moment I felt so alone and...tired and mentally exhausted (…) But I feel like everybody was more out for themselves and not really trying to really help each other (…).. I feel like the community was kind of broken." (age 16)
	Positive solitude	P100: "(I enjoyed) having me time. Like being able to do like stuff on my own." (age 15)<br>P107: "Um, things I enjoyed the most I'd probably say I got a lot of time to myself." (age 15)<br>P104: "I used to go for like walks around my neighbourhood **(…)** like I just used to reflect and it was such a like a peaceful time like after I came back home I thought like..I just felt like free. I felt like there was no **** nothing." (age 16)
	New connections	P101: "I met lots of new people and got to interact with some people during lockdown" (age 12)<br>P104: "(People could) talk to each other, sit down and really think like what is going on in the world." (age 16)<br>P104: "I definitely lost friendships and gained friendships.Like I found true friends and I also lost people that I thought were good friends to me" (age 16)

Superordinate theme two: Changes in mental health since the end of lockdown (table 2)

Autonomous adaptive coping skills

Being left alone with their thoughts led the young people to demonstrate great levels of resilience in the face of adversity. Indeed, 30% of them reported introspective self-evaluations and the development of adaptive self-regulating mechanisms. 50% of the participants observed growth in themselves, transpiring into their relationships with others. This demonstrates great resilience and resourcefulness amongst the young people.

For peer review only - http://bmjopen.bmj.com/site/about/guidelines.xhtml

Lifted restrictions

Lockdown ending meant newfound freedom, which 50% of the participants experienced as positively impacting their mental health. Restrictions being lifted allowed them to enjoy normal life again by being outside and regaining individual freedoms. Additionally, social bonds were reinstated, further benefitting their mental health.

Table 2: Changes in mental health experienced since the end of lockdown

Theme	Subtheme	Quotes and illustrations
Self-driven coping	Overthinking	P102: "I had to do it because if I didn't then how am I supposed to go in life so I literally just looked at the present and just focusing on what I need to do to get through my life." (age 15)<br>P103: "overthinking like I started to like really, evaluate my personality and like who I am as a person" (age 15)
	Better coping	P103: "those little things that I did like I used to go on walks or I just used to like I used to write down my thoughts a lot like it was just like random bits of what I'm feeling throughout the day and like those kind of help you feel better." (age 15)<br>P103: "when I realised it's like there so many different things that I can do to help myself and I just want to find all the right um outlets to project any um anger or aggression any uh sad feelings that I had without causing violence." (age 15)
	Personal growth	P102: **"**I've been depressed I know I've been all down and whatever, but I think if I didn't have that stage in my life I would not be what who I am right now" (age 15)<br>P103: **"**And um like I think it strengthened my relationships but it also strengthened the relationship that I had with myself which is like one of the most important one" (age 15)<br>P109:'Back then I wasn't as social (…) Cause I found a really good series of books and I just kept reading it.(…) It's not that I couldn't be social it's just that I wasn't bothered to, but now I'm like talking a bit more, still reading though." (age 14)

For peer review only - http://bmjopen.bmj.com/site/about/guidelines.xhtml

Lifted restrictions	Freedom	P103: "At the end of lockdown when everything like um the restrictions started lifting I felt like I was finally getting life back." (age 15)<br>P106: "going out in groups without having to get COVID tested themselves before they left the house" (age 13)<br>P107: "I took things for granted because obviously something as simple as being outside and going to the shops it's all changed." (age 15)
	Social bonds	P104: "Seeing my friends for the first time. Like I can't fake that that was like the best feeling cause it's like all these months I haven't seen you and it's like oh my god." (age 16)<br>P107: "when the restrictions first eased like in the summer of 2020. (…) Yeah I feel like then I got a lot closer with a lot of people." (age 15)
	New connections	P101: "I met lots of new people and got to interact with some people during lockdown" (age 12)<br>P104: "(People could) talk to each other, sit down and really think like what is going on in the world." (age 16)<br>P104: "I definitely lost friendships and gained friendships. Like I found true friends and I also lost people that I thought were good friends to me" (age 16)

Superordinate theme three: Support obtained during the Covid-19 pandemic and current needs (table 3)

Support

70% of the young people deplored a lack of support during the Covid-19 pandemic. In terms of structural support, 50% of the participants were critical of the academic help they were provided with. Additionally, 30% of them shared feeling neglected by society and governing bodies, evidenced by a deep mistrust in national institutions and strong feelings about being

For peer review only

overlooked, ignored and misunderstood as young people from black and mixed-ethnic minority backgrounds.

Needs

The young people requested various forms of support moving forward, the largest being psychological support (50%). In response to social bonds being challenged during the Covid-19 pandemic, 40% of the participants also requested relational support. With socialising being key to their wellbeing, support in this area would benefit them greatly. Finally, practical support with professional opportunities and maintaining a healthy routine, habits which were abruptly perturbed by the Covid-19 pandemic, was considered useful by 50% of the young people.

Table 3: Support obtained during the Covid-19 pandemic and current needs

**Theme**	**Subtheme**	**Quotes and illustrations**
Support	Lack of support	P100: "Honestly little to none, support yeah" (age 15)<br>P110: "I wouldn't say there was support, it was just really independent." (age 15)
	Neglect	P102: **"**I would actually listen to people because clearly the government doesn't listen to no one he just he does everything what he thinks and I don't I don't trust that" (age 15)<br>P102: "I know I have issues (…) It's just like, I need more support with that. (…) No one is listening. So I have to shout it out loud so everyone can listen" (age 15)<br>P104: "in this generation and age there are so many misconceptions about teenagers, especially me as a black female

For peer review only - http://bmjopen.bmj.com/site/about/guidelines.xhtml

		teenager, there's many misconceptions about…being…you know who you are or what you look like" (age 16)
	Structural	P100: "in school, it's very much like,…once in a blue moon ask us if we're alright and that's it." (age 15)<br>P106: "they don't help they only sit in my class and do nothing when I ask for help then sometimes they listen." (age 13)
Needs	Psychological	P104: "sometimes you just, you just need an ear. You just need someone to listen. I feel like that would really help actually. And it would've helped I think back then if I'd had that I think it would have been way easier to talk about." (age 16)<br>P104: "and (the therapist) just said what you described does sound like (depression), and I was like, in that moment like a tear fell on my face" (age 16)<br>P106: " (I would need) Like people speaking to me." (age 13)
	Relational	P105: "I would have like hugs from my friends. I feel like it's a way of comforting myself and comforting other people when it's like hardships and stuff, it just like helps a lot because even the simplest thing, like love can do a lot." (age 13)
	Practical	P107: "I would say maybe more opportunities available. So stuff like sports. Even something like work experience. Sort of that yeah I feel like that would help a lot cause it would help like ease back into things (…) making new friendships, relationships, has become a lot harder." (age 15)<br>P100: "About how to maintain a healthy lifestyle even though…circumstances can change like this (snaps fingers)." (age 15)

Discussion

This study investigated the impact of the Covid-19 pandemic on the mental health of young people from black and mixed-ethnic groups, how this has changed since the end of lockdown and the support that they may need to cope with these issues. Results indicated that

For peer review only - http://bmjopen.bmj.com/site/about/guidelines.xhtml

young people's mental health was both negatively and positively impacted by the Covid-19 pandemic. However, their mental wellbeing seemingly improved after the peak of the Covid-19 pandemic, due to adaptive coping mechanisms being developed and lockdown ending. Finally, the young people deplored a lack of support during the Covid-19 outbreak and were critical of the structural support they did receive. They asked for psychological, relational and practical support moving forward.

Firstly, participants reported experiencing the negative consequences of the Covid-19 pandemic on a personal level, in their relationships and on their mental health. This reinforces past research evidencing deteriorating mental health in young people from minority ethnic groups, both quantitatively [25, 28, 44] and qualitatively [26]. The present study nonetheless also demonstrated an improvement in the participants' mental health post-pandemic (Nov-Dec 2021), echoing wider studies reflecting this [26, 27]. McKinlay et al.'s results were not ethnic-specific [26], increasing the present study's relevance in focusing on the experiences of young people from black and mixed-ethnic minority groups. Additionally, the measures utilised in Penner et al.'s research were taken before the studied area's Covid-19 peak [27], limiting their validity. The present study however interviewed young people who have experienced multiple lockdowns, strengthening the results' validity. However, reports of past experiences are subject

For peer review only - http://bmjopen.bmj.com/site/about/guidelines.xhtml

to recall bias and individuals may overestimate positive affect retrospectively as a coping strategy, which potentially reduces the internal validity of the present study's results [45, 46].

The improvements in mental health post-lockdown found in our study, however, contrast with literature suggesting that, although observing a general amelioration in mental health symptoms after lockdown ended, scores remained higher than pre-pandemic [47]. This implies a long-lasting detrimental impact which was not observed in the present group of young people. This is potentially due to measurement differences as most quantitative studies measure changes in scores whilst qualitative ones focus on the most salient experience for the individual. Additionally, research suggests that although improvements in mental health after lockdown were observed amongst most young people, they did not manifest in at-risk youth [48]. The latter study's selection criteria for at-risk youth was household income, which although partially overlapping with ethnic minority, remains a distinctive non-interchangeable demographic [49]. This is not a criteria we used for our young people.

Finally, the young people deplored a lack of support during the Covid-19 outbreak and were critical of the structural support they received, echoing previous studies deploring poorly adapted mental health resources for individuals from ethnic minorities [18, 35]. Additionally, participants expressed feeling neglected by society and governing entities, mirroring previous

For peer review only

reports of alienation amongst individuals from minority ethnic groups [16, 22]. The present study's qualitative methodology thus effectively contributed to solidifying the existing body of literature around this theme. Study participants did not mention English proficiency as a barrier to them accessing mental health support, as was suggested by Sentell et al.'s research [34]. However, the participants are native English speakers, suggesting this obstacle may not apply to individuals from all ethnic minority groups. Similarly, stigma around mental health was not explicitly identified as an obstacle to mental health access contrary to Mahmood et al.'s findings [30]. In fact, study participants advocated for mental health awareness and its importance for public health discussion. One reason for this discrepancy in findings could be because participants in Mahmood et al.'s study were adult community leaders [30], a significantly older generation compared to the young people in our study, potentially reflecting a generational difference in perceptions of mental health, stigma, and willingness to discuss health issues. Fortunately, this might indicate that young people from black and mixed ethnic groups simply need more exposure to mental health knowledge overall and opportunities for open, non-judgmental discussions.

Strengths and limitations

For peer review only

For peer review only - http://bmjopen.bmj.com/site/about/guidelines.xhtml

A key strength this study possesses is its qualitative nature and in-person data collection method. By allowing young people to elaborate on their personal experiences, this study captured subtleties that wide-ranging mental health scales may have overlooked [50]. Additionally, as Covid-19 studies were mostly run mid-pandemic, sanitary restrictions compelled them to be conducted remotely. Although online and anonymised methods for interviewing have been associated with lower social desirability, they increase the risk of measurement error and misclassification [28, 51]. Virtual environments curb the effectiveness of non-verbal cues, affecting rapport which is vital when conducting sensitive research with vulnerable populations [52, 53]. The present study's in-person collection method of qualitative data enabled the researcher to invest time and effort in building rapport with the participants, increasing the results' validity and providing the participants with a valuable space to feel heard.

However, the wide range and variation in the study participants' age represents a key limitation. Indeed, participants ranged from 12-to-17 years-old. The convenience sampling method utilised gave the researcher no control over the specific ages of the young people included in the study. This is relevant as young people's experiences from late childhood to late adolescence vary widely [54]. During this period, they go through a time of significant personal development, within which age is a highly influential factor [23]. Their age, and

For peer review only - http://bmjopen.bmj.com/site/about/guidelines.xhtml

associated experiences, thereby inevitably introduce bias in the stories they told and the needs they voiced. That said, we recognise that our results do not reflect answers from the full age sample, containing mostly quotes from participants aged 15 and above, and encourage future research to further this endeavour with a larger representative sample. The results' generalisability to young people from minority ethnic groups overall is low, nonetheless capturing relevant perspectives from young people of ethnic minority groups.

Implications for practice and research

Young people's needs - psychological, relational and practical- encapsulated in this study resonate with current initiatives and policies being put into place by organisations, but should be sustained post-pandemic. Initiatives such as telehealth for young people from ethnic minority backgrounds [55] and the youth Covid-19 Support Fund [56] allowed for the continued provision of support and safe spaces for young people during the Covid-19 pandemic. Unfortunately, schemes supporting ethnic minority groups were highly hindered by lockdown and social distancing, limiting their scope for action [57]. Importantly, young people's involvement in the creation of frameworks that will impact them was identified by the United Nations Convention on the Rights of the Child as a fundamental human right [58]. Young people's voices and needs for support identified in the present study already had wide ranging

For peer review only - http://bmjopen.bmj.com/site/about/guidelines.xhtml

albeit short-term impact across stakeholders involved. Basing the workshops on young people's voices allowed previously unnoticed needs to be addressed, such as relational support, mental health, and practical help to enter the professional world. The charity – who engages with the young people on a weekly basis – gained a deeper understanding of the young peoples' needs and expanded the scope of possible initiatives beyond psychological support [18]. Student researchers involved also gained first-hand experience with a full timeline of the research process and insight into the widely differing experiences of individuals throughout the Covid-19 pandemic, which adds to the existing literature [3]. By bringing previously unnoticed voices into focus, in cooperation with the CopeWell project [38], this study exemplifies how community-based research can be a means to advocate for greater policy developments promoting racial justice in response to the Covid-19 outbreak [59]. Crucially, this study highlights the importance of developing grassroots support that is sustained through policy and practice for young people from black and mixed-ethnic groups in the aftermath of the Covid-19 pandemic and beyond.

Future research should proactively identify those unanswered needs and provide support, particularly for black and mixed ethnic young people through local co-produced solutions. Specifically, prior research solely focuses on assessing attitudes towards available

For peer review only - http://bmjopen.bmj.com/site/about/guidelines.xhtml

support or lack thereof, which may be less adaptable to future changes and needs. Conversely, our qualitative interviews provided the young people with a platform to share the assistance they would want. Through the process of giving voice to young people, they reported feeling heard and seen [60], giving strength to qualitative methods of inquiry considering long-term perspectives. However, this study is not without limitation. Participants' ethnicities were limited to black and mixed-ethnic groups and a wide age range, offering an incomplete picture of young people from ethnic minority groups' mental health in the Covid-19 context [61]. This highlights the need for future research to include varied samples of young people from minority ethnic groups in their qualitative investigations, yielding results with higher validity. Alternatively, age groups may be considered individually to tailor results and subsequent interventions to potential corresponding variations in needs. Studies may also extend to wider geographical areas, investigating cultural variations in the impact of the Covid-19 pandemic on young people from minority ethnic backgrounds, raising awareness towards this hitherto neglected issue on a larger scale. Finally, this study evidenced that the Covid-19 outbreak was cruelly reflective of social disparities in the UK, warranting the need to probe into other vulnerable groups' mental health in this context, namely individuals with disabilities or lower socio-economic statuses.

For peer review only

Conclusion

This qualitative interview study of young people from black and mixed-ethnic backgrounds during the Covid-19 pandemic has highlighted important challenges and barriers to mental health access and young people's need for support with mental health during Covid, as assessed in January to February 2021. Unexpectedly, study findings also highlighted young people's resilience and lessons learned when faced with global world challenges, such as coping mechanisms and the establishment of new relational connections. The co-production yielded an in-depth qualitative understanding of black and mixed minority ethnic young people's needs and support together with input from charity workers, student co-researchers, and academics. Future research studies with larger, more representative samples can enhance our understanding of the hardships vulnerable groups face in such unprecedent times, the strength and resilience they have, and can inform the development of support specific to the unjust burden they carry.

Acknowledgments I would like to extend my sincere thanks to my supervisor K.W. for her advice and continuous support, as well as to the young people and social workers who took part in this study for their time and contribution.

For peer review only - http://bmjopen.bmj.com/site/about/guidelines.xhtml

Contributorship statement: R.L. was involved with the data collection, analysing and interpretation of data, drafting, writing, reviewing, and final approval of the article. K.W. was involved with the conception of the research question, data collection, drafting, writing, reviewing, management of the project, supervision of the work, and final approval of the article. R.L. and K.W. agree to be accountable for all aspects of the work in ensuring that questions related to the accuracy or integrity of any part of the work are appropriately investigated and resolved.

Funding This work was supported by UKRI Research England. Awardees will be required to acknowledge support from UCL's HEIF Knowledge Exchange and Innovation Fund in any published outcomes (including media communication) associated with the award.

Competing interests None declared.

Patient consent for publication Not applicable.

Ethics approval Ethical approval was obtained from the UCL Institute of Education Ethics Committee (REC 1558). Informed consent was received from all participant and their parents.

Provenance and peer review Not commissioned; externally peer-reviewed

Data availability statement All data relevant to the study are included either in the article or the supplementary information.

For peer review only

Supplemental material This content has been supplied by the author(s). It has not been vetted by BMJ Publishing Group Limited (BMJ) and may not have been peer-reviewed. Any opinions or recommendations discussed are solely those of the author(s) and are not endorsed by BMJ. BMJ disclaims all liability and responsibility arising from any reliance placed on the content. Where the content includes any translated material, BMJ does not warrant the accuracy and reliability of the translations (including but not limited to local regulations, clinical guidelines, terminology, drug names and drug dosages), and is not responsible for any error and/or omissions arising from translation and adaptation or otherwise.

Open access This is an open access article distributed in accordance with the Creative Commons Attribution 4.0 Unported (CC BY 4.0) license, which permits others to copy, redistribute, remix, transform and build upon this work for any purpose, provided the original work is properly cited, a link to the licence is given, and indication of whether changes were made. See: https://creativecommons.org/ licenses/by/4.0/.

ORCID iD R.L.: 0000-0001-6277-8203. K.W.: 0000-0002-2962-8438

For peer review only

References

1 Tisdell, C. A. (2020). Economic, social and political issues raised by the Covid-19 pandemic. *Economic Analysis and Policy*, *68*, 17-28. https://doi.org/10.1016/j.eap.2020.08.002

2 World Health Organization (2020). Coronavirus (Covid-19) Dashboard. *World Health Organization*. Available online: https://covid19.who.int/

3 Wong, K. K. Y., Loke, K., & Melville, K. M. K. L. (2022). Reflections, Resilience, and Recovery: A qualitative study of COVID-19's impact on an international adult population's mental health and priorities for support. *UCL Open: Environment Preprint*

4 Tai, D. B. G., Shah, A., Doubeni, C. A., Sia, I. G., & Wieland, M. L. (2021). The disproportionate impact of COVID-19 on racial and ethnic minorities in the United States. *Clinical Infectious Diseases*, 72(4), 703-706. https://doi.org/10.1093/cid/ciaa815

5 Aldridge, R. W., Lewer, D., Katikireddi, S. V., Mathur, R., Pathak, N., Burns, R Fragaszy, E.B., Johnson, A.M., Devakumar, D., Abubakar, I. and Hayward, A. (2020). Black, Asian and Minority Ethnic groups in England are at increased risk of

For peer review only - http://bmjopen.bmj.com/site/about/guidelines.xhtml

death from Covid-19: Indirect standardisation of NHS mortality data. *Wellcome Open Research*, *5*. https://dx.doi.org/10.12688%2Fwellcomeopenres.15922.2

6 Intensive Care National Audit and Research Centre (ICNARC). (2022). ICNARC Covid-19 report on critical care. *ICNARC Case Mix Programme Database*. https://www.icnarc.org/Our-Audit/Audits/Cmp/Reports

7 Anderson, K. F. (2013). Diagnosing discrimination: Stress from perceived racism and the mental and physical health effects. *Sociological Inquiry*, *83*(1), 55-81. https://doi.org/10.1111/j.1475-682X.2012.00433.x

8 Malhotra, A., Kamepalli, R. K., & Bamrah, J. S. (2020). Perspective: Poor metabolic health is a major issue for increased Covid-19 mortality in BAME groups. *The Physician International Journal of Health*, *6*(2). https://doi.org/10.38192/1.6.2.4

9 Bronfenbrenner, U. (1979). *The ecology of human development: Experiments by nature and design*. Harvard University Press.

10 Camargo, A., Hossain, E., Aliko, S., Akinola-Odusola, D., Artus, J., & Kelman, I. (2021). Using an ecological and biological framing for an anti-racist Covid-19 approach. *MedRxi*. https://doi.org/10.1101/2021.01.24.21250397

For peer review only - http://bmjopen.bmj.com/site/about/guidelines.xhtml

11 Phiri, P., Delanerolle, G., Al-Sudani, A., & Rathod, S. (2021). Covid-19 and Black, Asian, and minority ethnic communities: A complex relationship without just cause. *Journal of Medical Internet Research Public Health and Surveillance*, *7*(2), e22581. https://doi.org/10.2196/22581

12 Ministry of Housing, Communities and Local Government (2020) *People living in deprived neighbourhoods.* UK Government. https://www.ethnicity-facts-figures.service.gov.uk/uk-population-by-ethnicity/demographics/people-living-in-deprived-neighbourhoods/latest#main-facts-and-figures

13 Carrión, D., Colicino, E., Pedretti, N. F., Arfer, K. B., Rush, J., DeFelice, N., & Just, A. C. (2020). Assessing capacity to social distance and neighborhood-level health disparities during the Covid-19 pandemic. *MedRxiv*. https://doi.org/10.1101/2020.06.02.20120790

14 NHS Digital (2020). *NHS workforce ethnicity facts and figures.* UK Government. https://www.ethnicity-facts-figures.service.gov.uk/workforce-and-business/workforce-diversity/nhs-workforce/latest#full-page-history

15 Catney, G., & Sabater, A. (2015). *Ethnic minority disadvantage in the labour market:*

For peer review only

Participation, skills and geographical inequalities. Joseph Rowntree Foundation, pp. 1-96. https://www.jrf.org.uk/file/46593/download?token=-98tkg_Q&filetype=full-report

16 Van Bavel, J. J., Baicker, K., Boggio, P. S., Capraro, V., Cichocka, A., Cikara, M., Crocket, M.J., Crum A.J., Douglas, K.M., Druckman, J.N., Drury, J., Dube, O., Ellemers, N., Finkel, E.J., Fowler, J.H., Gelfand, M., Han, S., Alexander Haslam, S., Jetten, J., Kitayama, S., Mobbs, D., Napper, L.E., Packer, D.J., Pennycook, G., Peters, E., Petty, R.E., Rand, D.G., Reicher, S.D., Schnall, S., Shariff, A., Skitka, L.J., Smith, S.S., Sunstein, C.R., Tabri, N., Tucker, J.A., van der Linden, S., van Lange, P., Weeden, K.A., Wohl, M.J.A., Zaki, J., Zion, S.R., & Willer, R. (2020). Using social and behavioural science to support Covid-19 pandemic response. *Nature Human Behaviour*, *4*(5), 460-471. https://doi.org/10.1038/s41562-020-0884-z

17 Public Health England. (2020) *Beyond the data: Understanding the impact of Covid-19 on BAME communities.* Department of Health and Social Care. https://assets.publishing.service.gov.uk/government/uploads/system/uploads/attachment_data/file/892376/COVID_stakeholder_engagement_synthesis_beyond_the_data.pdf

18 Mind (2020) The mental health emergency: How has the coronavirus pandemic impacted our mental health? Mind. Available at: mind.org.uk

19 Geyser, L. (2021). The impact of Covid-19 on food security. A qualitative analysis of the rise in London's existing food insecurity due to the financial impact of Covid-19. *World Nutrition*, *12*(1), 40-50. https://doi.org/10.26596/wn.202112140-50

20 Alesina, A., & La Ferrara, E. (2002). Who trusts others?. *Journal of Public Economics*, *85*(2), 207-234. https://doi.org/10.1016/S0047-2727(01)00084-6

21 Demaris, A., & Yang, R. (1994). Race, alienation, and interpersonal mistrust. *Sociological Spectrum*, *14*(4), 327-349. https://doi.org/10.1080/02732173.1994.9982075

22 Farquharson, W. H., & Thornton, C. J. (2020). Debate: Exposing the most serious infirmity–racism's impact on health in the era of COVID-19. *Child and Adolescent Mental Health*, *25*(3), 182-183. https://doi.org/10.1111/camh.12407

23 Egan, S. M., Pope, J., Moloney, M., Hoyne, C., & Beatty, C. (2021). Missing early education and care during the pandemic: The socio-emotional impact of the Covid-19 crisis on young children. *Early Childhood Education Journal*, 1-10. https://doi.org/10.1007/s10643-021-01193-2

For peer review only - http://bmjopen.bmj.com/site/about/guidelines.xhtml

24 Dewa, L. H., Crandell, C., Choong, E., Jaques, J., Bottle, A., Kilkenny, C., Lawrence-Jones, A., Di Simplicio, M., Nicholls, D., & Aylin, P. (2021). CCopeY: A mixed-methods coproduced study on the mental health status and coping strategies of young people during Covid-19 UK lockdown. *Journal of Adolescent Health*, *68*(4), 666-675. https://doi.org/10.1016/j.jadohealth.2021.01.009

25 Stinson, E. A., Sullivan, R. M., Peteet, B. J., Tapert, S. F., Baker, F. C., Breslin, F. J., Dick, A.S., Gonzalez, M.R., Guillaume, M., Marshall, A.T., McCabe, C.J., Pelham III, W.E., Van Rinsveld, A., Sheth, C.S., Sowell, E.R., Wade, N.E., Wallace, A.L. & Lisdahl, K. M. (2021). Longitudinal impact of childhood adversity on early adolescent mental health during the Covid-19 pandemic in the ABCD Study Cohort: Does race or ethnicity moderate findings?. *Biological Psychiatry Global Open Science*, *1*(4), 324-335. https://doi.org/10.1016/j.bpsgos.2021.08.007

26 McKinlay, A. R., May, T., Dawes, J., Fancourt, D., & Burton, A. (2021). You're just there, alone in your room with your thoughts: A qualitative study about the impact of lockdown among young people during the Covid-19 pandemic. *MedRxiv.* https://doi.org/10.1101/2021.04.11.21254776

27 Penner, F., Ortiz, J. H., & Sharp, C. (2021). Change in youth mental health during the

For peer review only - http://bmjopen.bmj.com/site/about/guidelines.xhtml

Covid-19 pandemic in a majority Hispanic/Latinx US sample. *Journal of the American Academy of Child & Adolescent Psychiatry*, 60(4), 513-523. https://doi.org/10.1016/j.jaac.2020.12.027

28 Knowles, G., Gayer-Anderson, C., Turner, A., Dorn, L., Lam, J., Davis, S., Blakey, R., Lowis, K., Schools Working Group, Young Persons Advisory group, Pinfold, V., Creary, N., Dyer, J., Hatch, S.L., Ploubidis, G., Bhui, K., Harding, S., & Morgan, C. (2022). Covid-19, social restrictions, and mental distress among young people: A UK longitudinal, population-based study. *Journal of Child Psychology and Psychiatry.* https://doi.org/10.1111/jcpp.13586

29 Pascual-Sanchez, A., Nicholls, D., Patalay, P., Crosby, L., McColoud, T., Hudson, L., & Viner, R. (2020). You-COPE: Mental health consequences experienced by young people aged 16-24. *University College London. https://www.ucl.ac.uk/child-health/sites/child_health/files/youcope_briefing_mental_health_impact_final_version.pdf*

30 Mahmood, F., Acharya, D., Kumar, K., & Paudyal, V. (2021). Impact of Covid-19

For peer review only - http://bmjopen.bmj.com/site/about/guidelines.xhtml

pandemic on Black, Asian and Minority Ethnic (BAME) communities: A qualitative study on the perspectives of BAME community leaders. *MedRxiv.* https://doi.org/10.1101/2021.03.03.21252286

31 Gillard S., Dare C., Hardy J., Nyikavaranda P., Rowan Olive R., Shah P., Birken M., Foye U., Ocloo J., Pearce E., Stefanidou T., Pitman A., Simpson A., Johnson, S., Lloyd-Evans, B., & NIHR Mental Health Policy Research Unit Covid coproduction research group. Experiences of living with mental health problems during the COVID-19 pandemic in the UK: A coproduced, participatory qualitative interview study. *Social Psychiatry and Psychiatry Epidemiology 56*, 1447–1457 (2021). https://doi.org/10.1007/s00127-021-02051-7

32 Dewa, L. H., Roberts, L. F., Choong, E., Crandell, C., Demkowicz, O., Ashworth, E., Branquinho, C., & Scott, S. (2022). The impact of COVID-19 on young people's mental health and wellbeing from a European perspective: A co-produced qualitative systematic review. *Social Science Research Network.* 4266185

33 London Assembly Health Committee (2015). *Healthy minds, healthy Londoners improving access to mental health services for London's young and Black, Asian and minority ethnic population.* Available from:

For peer review only - http://bmjopen.bmj.com/site/about/guidelines.xhtml

https://www.london.gov.uk/sites/default/files/gla_migrate_files_destination/HealthyMindsHealthyLondoners_0.pdf.

34 Sentell, T., Shumway, M., & Snowden, L. (2007). Access to mental health treatment by English language proficiency and race/ethnicity. *Journal of General Internal Medicine*, *22*(2), 289-293. https://doi.org/10.1007/s11606-007-0345-7

35 Khan, S., Asif, A., & Jaffery, A. E. (2021). Language in a time of Covid-19: literacy bias ethnic minorities face during Covid-19 from online information in the UK. *Journal of Racial and Ethnic Health Disparities*, *8*(5), 1242-1248. https://doi.org/10.1007/s40615-020-00883-8

36 Pro Bono Economics (2020), November 15-20 Covid Charity Tracker Survey results. *PBE Covid Charity Tracker.* Available at: https://www.probonoeconomics.com/november-15-20-covid-charity-tracker-survey-results

37 Youth Employment UK (2019). Youth Voice Census Report 2019. *Youth Employment UK.* Available at: https://www.youthemployment.org.uk/dev/wp-content/uploads/2019/05/2019-Youth-Employment-UK-Report_FINAL-1.pdf

For peer review only - http://bmjopen.bmj.com/site/about/guidelines.xhtml

38 Wong, K. K.-Y. (2021, December 4). COVID19: Co-creating psychological wellbeing and life-skill workshops with BAME youths in West London (COPE-WELL). https://doi.org/10.17605/OSF.IO/JCAK7

39 Smith, J. A. (1996). Beyond the divide between cognition and discourse: Using interpretative phenomenological analysis in health psychology. *Psychology and Health*, *11*(2), 261-271. https://doi-org.libproxy.ucl.ac.uk/10.1080/08870449608400256

40 Smith, J. A., & Osborn, M. (2015). Interpretative phenomenological analysis as a useful methodology for research on the lived experience of pain. *British Journal of Pain*, *9*(1), 41-42. https://dx.doi.org/10.1177%2F2049463714541642

41 Smith, J. A., & Shinebourne, P. (2012). Interpretative phenomenological analysis. In H. Cooper, P. M. Camic, D. L. Long, A. T. Panter, D. Rindskopf, & K. J. Sher (Eds.), *APA handbook of research methods in psychology, Vol. 2. Research designs: Quantitative, qualitative, neuropsychological, and biological* (pp. 73–82). American Psychological Association. https://doi.org/10.1037/13620-005

For peer review only

42 Alase, A. (2017). The interpretative phenomenological analysis (IPA): A guide to a good qualitative research approach. *International Journal of Education and Literacy Studies*, *5*(2), 9-19. http://dx.doi.org/10.7575/aiac.ijels.v.5n.2p.9

43 Goldspink, S., & Engward, H. (2019). Booming clangs and whispering ghosts: Attending to the reflexive echoes in IPA research. *Qualitative Research in Psychology*, *16*(2), 291-304. https://doi.org/10.1080/14780887.2018.1543111

44 Wade, M., Prime, H., & Browne, D. T. (2020). Why we need longitudinal mental health research with children and youth during (and after) the Covid-19 pandemic. *Psychiatry Research*, *290*, 113143. https://dx.doi.org/10.1016%2Fj.psychres.2020.113143

45 Colombo, D., Suso-Ribera, C., Fernández-Álvarez, J., Cipresso, P., Garcia-Palacios, A., Riva, G., & Botella, C. (2020). Affect recall bias: Being resilient by distorting reality. *Cognitive Therapy and Research*, *44*(5), 906-918. https://doi.org/10.1007/s10608-020-10122-3

46 Raphael, K. (1987). Recall bias: A proposal for assessment and control. *International Journal of Epidemiology*, *16*(2), 167-170. https://doi.org/10.1093/ije/16.2.167

For peer review only - http://bmjopen.bmj.com/site/about/guidelines.xhtml

47 Richter, D., Riedel-Heller, S., & Zuercher, S. (2021). Mental health problems in the general population during and after the first lockdown phase due to the SARS-Cov-2 pandemic: Rapid review of multi-wave studies. *Epidemiology and Psychiatric Sciences*, 1-17. https://doi.org/10.1017/S2045796021000160

48 Skripkauskaite, S., Shum, A., Pearcey, S., McCall, A., Waite, P., & Creswell, C. (2021). Changes in children's mental health symptoms from March 2020 to June 2021 (Report 11). *Co-SPACE Study*. http://cospaceoxford.org/findings/changes-in-childrens-mental-health-symptoms-from-march-2020-to-june- 2021/

49 Department for Work and Pensions (2021) *Family resources survey (FRS) for the financial year 2019 to 2020, providing information on income and circumstances of UK households.* UK Government. https://www.ethnicity-facts-figures.service.gov.uk/work-pay-and-benefits/pay-and-income/household-income/latest#download-the-data

50 Peters, S. (2010). Qualitative research methods in mental health. *Evidence-based Mental Health*, *13*(2), 35-40. http://dx.doi.org/10.1136/ebmh.13.2.35

51 Dayan, Y., Paine, C. S., & Johnson, A. J. (2007). Responding to sensitive questions in

For peer review only

surveys: A comparison of results from online panels, face to face, and self-completion interviews. *World Association for Public Opinion Research.*

52 Dempsey, L., Dowling, M., Larkin, P., & Murphy, K. (2016). Sensitive interviewing in qualitative research. *Research in Nursing & Health*, *39*(6), 480-490. https://doi.org/10.1002/nur.21743

53 Shapka, J. D., Domene, J. F., Khan, S., & Yang, L. M. (2016). Online versus in-person interviews with adolescents: An exploration of data equivalence. *Computers in Human Behavior*, *58*, 361-367. https://doi.org/10.1016/j.chb.2016.01.016

54 Solmi, M., Estradé, A., Thompson, T., Agorastos, A., Radua, J., Cortese, S., … & Ifteni, P. (2022). Physical and mental health impact of COVID-19 on children, adolescents, and their families: The Collaborative Outcomes study on Health and Functioning during Infection Times-Children and Adolescents (COH-FIT-C&A). *Journal of Affective Disorders,* 299, 367-376. https://doi.org/10.1016/j.jad.2021.09.090

55 Rajgopal, A., Li, C. R., Shah, S., & Sundar Budhathoki, S. (2021). The use of telehealth to overcome barriers to mental health services faced by young people from

For peer review only - http://bmjopen.bmj.com/site/about/guidelines.xhtml

Afro-Caribbean backgrounds in England during the Covid-19 pandemic. *Journal of Global Health*, 11, 03040. https://doi.org/10.7189/jogh.11.03040

56 Hammelsbeck, R., Mitchell, M., Thornton, I., Caroline, W., Taylor, B., & Bristow, T. (2021). Evaluation of the Youth Covid-19 Support Fund. *National Centre for Social Research.* *https://dera.ioe.ac.uk//38969/1/Youth_Covid-19_Support_Fund_-_Evaluation_Report_final__WEB_.pdf*

57 Murray, K. (2020). Impact of Covid-19 on the BAME community and voluntary sector. *The Ubele Initiative.* https://www.ubele.org/assets/documents/REPORT-Impact-of-Covid-19-on-the-BAME-Community-and-voluntary-sector-30-April-2020.pdf

58 UN General Assembly (1989), *Convention on the Rights of the Child.* United Nations, Treaty Series, vol. 1577. https://www.unicef.org/child-rights-convention/convention-text

59 Hu, Y. (2020). Intersecting ethnic and native–migrant inequalities in the economic impact of the Covid-19 pandemic in the UK. *Research in Social Stratification and Mobility*, *68*, 100528. https://doi.org/10.1016/j.rssm.2020.100528

For peer review only - http://bmjopen.bmj.com/site/about/guidelines.xhtml

60 Hamilton, S., Pinfold, V., Cotney, J., Couperthwaite, L., Matthews, J., Barret, K., Warren, S., Corker, E., Rose, D., Thornicroft, G., & Henderson, C. (2016). Qualitative analysis of mental health service users' reported experiences of discrimination. *Acta Psychiatrica Scandinavica*, 134, 14-22. https://doi.org/10.1111/acps.12611

61 Proto, E., & Quintana-Domeque, C. (2020). Covid-19 and mental health deterioration among BAME groups in the UK. *Social Science Research Network.* https://dx.doi.org/10.2139/ssrn.3662057

For peer review only

Appendix A

Initial Interview Schedule

Introduce the research team and build rapport. Present the consent form and information sheet, go over ethics and take questions. Finally, explain rationale of the study, why we are here, aim to build trust.

Part 1- *Broad questions about impact of Covid-19*

1. How has your experience of the pandemic been?

   1.1. If mention education, how did it feel not going to school anymore?

   1.2. If mention home environment, how did it feel spending more time at home?

2. How were your relationships with your friends and family?

3. What were some of your favourite or least favourite things about covid?

4. How did covid impact your physical health?

   4.1. If mention close experience with covid, how did that made them feel?

5. How did Covid-19 impact your mental health?

   5.1. If mention of worry or such feelings, how did that feel like? what made it worse or better for you? If you opened up, who did you feel comfortable talking to?

6. Has your perception of the media, governments and institutions during the pandemic affected your trust in them?

Part 2- *Changes throughout the pandemic*

7. What about now, how have things changed since then?

   7.1. If no direction, what are the biggest challenges now? What were the biggest changes in your life?

8. How have your physical and mental health changed since lockdown?

   8.1. If need guidance, are they better or worse now?

For peer review only

9. How is your perception of the media, governments and institutions now, after the peak of the pandemic has passed and how has your trust in them changed?

Part 3- *Support needed to deal with these issues*

10. Did you receive any mental support during the pandemic?

    10.1. If so, how did you access it? What form did the support take and how did it make you feel?

    10.2.If not, did you feel like you would have needed some? what kind?

11. What aspects of your life do you feel you would need more support in?

12. What kind of support would you need now and how would you like to access it?

Debrief, thank you and share next steps of the research project.

For peer review only

Appendix B

Final Interview Schedule

Introductions - Researchers will introduce themselves, along with JED CEO and social worker and any student researchers present. Thank young people for taking part and joining the focus group. Build rapport. Walk through participant information sheet with young people, read verbatim each item on the consent form to ensure participants understand. Take questions.

Lay groundwork for why we're conducting this project, project aims, what we hope to learn and project outputs.

Express ethical considerations (right to withdraw at any time, don't have to share information they don't want to, don't have to answer all questions, confidentiality, trust, safe space to share, no right or wrong answers). Again, emphasize that researchers are here to learn from young people, in order to help other young people in the future as we navigate out of the COVID pandemic. Remind young people the session will be split into 2 parts and last between 30-40 minutes.

QUESTIONS

Part 1 – barriers to health access, impact of covid on health and changes

1. Thinking over this past year, how has it been for you?
    a. Prod: What are some things you are grateful for, or challenges that you faced?
2. Did you learn anything about yourself or others around you? How were you close relationships?
3. Thinking about this past year, has the pandemic affected the way you trust in others? Institutions, governments, community, people?
4. What were some highlights?
5. How has covid impacted your health – mentally and physically?

For peer review only

6. Thinking about pre-pandemic and now, what were some things that has changed for the better? Or for the worse?
7. Thinking about pre-pandemic and now, how has your health changed for the better? Or for the worse?
8. Thinking about some of your challenges, what are some examples of your struggles/challenges?
9. What did you enjoy or least enjoy during the pandemic?

Part 2 – Support that young people would like or need

1. What support did you receive during the pandemic, if any?
2. How was the support that you received, if any?
    a. How did you come across this support?
    b. What were the barriers to the support? If any?
    c. If no barriers, what made it accessible?
3. If you could get more support during the pandemic – and now – what might you want/need?
4. Thinking specifically about your health (physical/mental), what do you wish you had known? What support would you want?
5. What do you think are the key areas that you need most support on? Where might you go to get support?

Debrief and thank young people for their time. Inform next steps in project on co-creating workshops together

For peer review only

Appendix C

Patterns clusters

… : aim 1: mental health

Patterns of negative mental health
ISOLATION FROM OTHERS: the most
Isolation IIIIIIIIIIIII **Loneliness** IIIIIIIII **Broken community** II **Lack of social interactions** IIIIIIII **Loosing friendships** IIII
ILLNESS-RELATED DISTRESS: the 2nd most
COVID anxiety IIIIII **Death** IIII **Media overload** II **Overwhelming focus on physical** II
FAMILY ISSUES: sometimes
Home environment III
EDUCATION: sometimes
Online learning IIII
BAD MENTAL HEALTH: often
Overall hard MENTAL HEALTH IIIII
TRUST: often
Trust issues (friends/institutions) IIII
SADNESS: frequent
Sadness II **Depression** IIIII
STRESS: often
Stress and anxiety III **Overthinking** I **Fear** I
CLINICAL: frequent
Suicide/suicidal ideations III **Eating disorders/body image** IIIIII **ADHD** I
LACK OF CONTROL: sometimes
Lack of usual coping/escape mechanisms II **Helpless** I
SELF-ESTEEM: frequent
Self-esteem IIIIIII
APATHY/NUMB: frequent
Laziness III **Loss of structure** I **Lack of motivation** IIIII **Nonchalance** I **Boring** I **Tired** III
ANGER: often
Anger II **Frustration** IIII **Triggering** I

Young people III **Collective denial** I

Patterns of positive mental health
CLOSER INTERACTIONS: sometimes
Closer bonds (family, friends) III **Meeting people online** I
GRATEFULNESS: often
Grateful IIII
NEW HABITS: the most
New mindful practices IIIIIIIII
SOLITUTDE: often
Alone time II **Resilience** II **Self-actualisation** IIII
EDUCATION: rarely
Time to study II

For peer review only

…: aim 2: changes with time

NOSTALGIA: rarely
Nostalgia I
SOCIAL INTERACTION HELPS: the most
After lockdown peak with seeing people again IIIIIIIIIIII
SELF-RELIANCE SO IMPROVEMENT WITH TIME: the 2nd most
Mental health improving because of self-reliance after bad period/lockdown IIIIIII
GRATEFUL: rarely
Grateful for themselves, for being safe I

…: aim 3: support

During COVID
LACK OF SUPPORT: the most
Lack of IIIIII
SCHOOL: the 2nd most
School lacks/ don't want IIIIII **School as a point of access** II
CLOSE PEOPLE: often
Friends II **Self-help** I **Family** II **Lack of family** I
LACK OF LEGITIMITY: often
illness not seen as legitimate (esp awareness ass BAME YP+gender) III **Reluctant to reach out** II
FINANCIAL/TECHNICAL: sometimes
Financial I **Technical** I

Now Want
ACTIVITIES: frequent
Activities IIIII
MOTIVATION: sometimes
Healthy lifestyle I **Motivational support** I
MENTAL HEALTH: most often
Mental health IIIIII **Intense relief when getting support** I
EDUCATION: often
Educational I **School support** II
YOUTH CLUBS: often
Youth club III
ARTS: frequent
Arts IIII
TO BE HEARD: 2nd most
Listened to IIIII
TO LEARN: sometimes
Learning about new issues II
SPORTS: often
Social sports IIII

For peer review only

WORK EXPERIENCE: rarely
Work experience I

Reaching out I
LACK OF: sometimes
Lack of adequate support (safe, accessible) II
SCHOOL AS A BARRIER: sometimes
School as a barrier II

For peer review only

Appendix D

Initial Thematic Tables

Superordinate theme 1: Impact on mental health	
**A. Loneliness**<br><br>*Lack of social interactions*<br>P103: "lockdown obviously had a like really big impact on my mental health like it made me feel way more alone that like I actually was cause I couldn't go outside obviously."<br>P103: "I enjoyed the alone time the most but then it's also what like triggered me the most. I just kept I felt so trapped (…) And isolated but I also felt isolated and also felt like alone and unable to be myself in my own space."<br>P104: "But in that moment you're just like…I just want to be alone and it doesn't help it's like you're scurrying into a little hole (…) So I feel like also not having…people to talk to cause you can have like a million people around you and still feel alone. So I felt like in that moment I felt so alone and...tired and mentally exhausted and then school, and then.. not seeing friends and family members and the death it all just- (…) I feel like a lot of people different ages went through that same experience with like it just starts affecting you."<br>P106: "I felt actually quite sad (…) and angry because I'm at home. And like I just feel too much like energy in the house and you're too hot and you can't do this you can't do that and like you can't go out and that's like what frustrates me"<br><br>*Self-esteem*<br>P103: "I had my brothers and my mum obviously but like they don't understand obviously, and um like I just really need someone to tell me like you're good enough (…) and like that's all like that's all anyone needs to hear, they just want validation (…) I just want to feel appreciated and like-like I just want to feel stuff like that."<br>P103: "There was a lot of challenges with like my mental health and like my self-esteem and like I- I still don't but like, I never believed in myself like at all, like at all/ (…) before I had no faith in myself like I used to believe that I couldn't do anything, I just like feel like I was never going to be good enough"	**B. Mental disorders**<br><br>*Anxiety, Depression and Disordered eating*<br>P104: "but as well as mentally it really deteriorated like all youth, people my age like I realised that everybody was finding out they had depression, anxiety, it was really like draining on staying put, some people had toxic family members they had to stay home it was really like..it was hard."<br>P104: "I realised a lot of things which kind of drove me into a.. I would say quite a depression in the sense of like I wouldn't.. I would just staying in my room the whole time, I wouldn't leave it, stay on my phone but then log off every social media app possible to kind of just block out everything"<br>P103: "I lost a lot of weight after the pandemic not in the best way. I didn't really eat, um but like (…)I never really used to- used to give myself like the attention I deserve, um like um and do you know with COVID it made it very very bad like my mental health was very bad, I was down there it was so bad. (…) and it was so such a heavy weight on my heart, it was like it was really hard for me and COVID made it all worse like trapped in my room seeing the same four walls everyday (…) so I was losing it, I was losing it."<br>P100: "Mentally, it affected me both like oh am I going to get COVID next, like got some sort of anxiety maybe with me."<br>P102: "Umm I've been sad, depressed…It was hard but I had to do it"<br><br>*Trauma*<br>P104: "Like it just caused a lot of like thinking and..kind of just a lot of like reevaluating life and everything especially being in lockdown in your house and people passing away drastically like every day the numbers were increasing like I felt (…) there was times you really have to stop and think like this type of like disease this thing is wiping everybody out (…) anybody could have died from it."<br>P104: "This is really happening and nobody was grasping with the fact that people are really dying, (…) like it was sad that so many people

For peer review only

P104: "I realised that I had a lot of mental health problems within myself, and I wasn't not really happy, um not very confident, quite insecure"<br><br>*Lack of trust in others/institutions*<br>P104: "But I feel like everybody was more out for themselves and not really trying to really help each other (…) so it may have cause some type of division.. which kind of broke- I feel like the community was kind of broken (…) Like at some point, we weren't together, collective and stuff."<br><br>*Lack of motivation*<br>P107: "And also um I feel like because I haven't been doing a lot I've lost the motivation to actually do things."<br>P107: "Um mentally, I think yeah as I said before my motivation has taken like a big decline and also obviously I feel like bad habits during COVID there's not really much that you can do."<br>P110: "A lot, uh yeah like laziness, um can't get out of bed as much, um yeah just a lot of that."	couldn't handle COVID they took their lives like it was..it was absolutely devastating really, so yeah it was just so sad."<br><br>**C. Positive impact on mental health**<br><br>*New connections*<br>P101: "I met lots of new people and got to interact with some people during lockdown so that was good,"<br>P104: "(People could) talk to each other, sit down and really think like what is going on in the world."<br>P105: "but like we have gotten closer and we like text my friends during in the middle of class when I was in lockdown and we'd sometimes have video calls and stuff"<br>P107: "Yeah um I've got a lot closer with a lot of my friends"<br><br>*New mindful activities*<br>P100: "Um, I was able to try things that I wouldn't try on a day to day basis (…) like going for walks and stuff and like doing drawings."<br>P101:'I started to find new hobbies like poetry, um and we started going to this poetry night which was like good"<br>P104: "during summer like obviously summer it gets dark really late um I used to go for like walks around my neighbourhood and obviously I had like no phone nothing so like I would just like I just used to reflect and it was such a like a peaceful time like after I came back home I thought like..I just felt like free. I felt like there was no **** nothing."<br><br>*Solitude*<br>P100: "(I enjoyed) having me time. Like being able to do like stuff on my own."<br>P107: "Um, things I enjoyed the most I'd probably say I got a lot of time to myself."

Superordinate theme 2: Changes in mental health since the start of lockdown	
**A. Post-lockdown high**<br><br>*Freedom*<br>P103: "But like at the end of lockdown when everything like um the restrictions started lifting I felt like I was finally getting life back."	**B. Gradual mental health coping**<br><br>*Isolation and overthinking*<br>P102: "I had to do it because if I didn't then how am I supposed to go in life so I literally just looked at the present and just focusing on what I need to do to get through my life."

For peer review only

P106: "going out in groups without having to get COVID tested themselves before they left the house."<br>P107: "Cause now that I've finally got the freedom to be out of my house (…) I was out of my house all the time."<br><br>*Social closeness*<br>P104: "Seeing my friends for the first time. Like I can't fake that that was like the best feeling cause it's like all these months I haven't seen you and it's like oh my god."<br>P104: "And I would see no one, it was so.. everyone was scared for their lives, like nobody was outside..it was like (sigh) so I guess when I started seeing the community, everybody kind of coming, trying to make this new normal, I guess that was really nice"<br>P107: "when the lockdown when the restrictions first eased like in the summer of 2020. (…) Yeah I feel like then I got a lot closer with a lot of people."<br>P109: "Back then I wasn't as social (…) Cause I found a really good series of books and I just kept reading it.(…) It's not that I couldn't be social it's just that I wasn't bothered to, but now I'm like talking a bit more, still reading though."<br>P110: "Good, (friends and family relationships) got even stronger since we could interact again."	P103: "Cause it was a very long time just self-reflecting and like, I liked it- it was very therapeutic but at the same time I hated it I felt like I was being cooped up and like trapped/"<br>P103: "started overthinking like I started to like really, evaluate my personality and like who I am as a person (…) and like my mental health as well cause I've never really thought about mental health at all, ok I'm not ok but there's nothing I can do"<br>P103: "before the pandemic I never really like cared about myself (…)I think that was mainly because there was all this stuff like all this stuff that was just built up and I never had a chance to release it cause I was always (…) so I never had to think about it."<br><br>*Better coping*<br>P102: "I have ADHD (…) So I know what time I'll be coming home and (my mum) will have checked on me cause I'll be doing silly stuff so, like stuff like that has changed me and normally it would ne- I would never do that so I'm happy that I've done that. (…)I'm organised better. (…) So that's like COVID, I think COVID actually helped me do all of this stuff, so yeah"<br>P103: "those little things that I did like I used to go on walks or I just used to like I used to write down my thoughts a lot like it was just like random bits of what I'm feeling throughout the day and like those kind of help you feel better."<br>P103: "when I realised it's like there so many different things that I can do to help myself and I just want to find all the right um outlets to project any um anger or aggression any uh sad feelings that I had without causing violence."<br><br>*Better mental health*<br>P102: "Uh, in my opinion I've known I've been depressed I know I've been all down and whatever, but I think if I didn't have that stage in my life I would not be what who I am right now"<br>P102: "I think I've changed for the better cause I used to be like a naughty kid. (…) So now I've like realised like I need to learn, revise,.. no friends like everything changed and I kind of like it because I've never been like this before so"<br>P102: "So like, a few years ago I was crazy, I was hanging around with the wrong crowd, and now I've realised like, I've realised I've grown and I have to show my little sister that I can't be like who I am right now, so I had to change my

For peer review only

	personality just for her (…) cause if it wasn't for her then I would still be behaving bad and yeah'<br>P103: "And um like I think it strengthened my relationships but it also strengthened the relationship that I had with myself which is like one of the most important ones, so yeah'<br>P103: "I think I'm good now, I think if you'd asked- I think if you'd done this interview like a couple months ago (…) I was so stuck, I would have been like I need someone to just talk to."

Superordinate theme 3: Support obtained during pandemic and needs now	
**A. Support**<br><br>*Lack of support*<br>P100: "Honestly little to none, support yeah"<br>P103: "I wish I would've know that there's people that I can talk to, or like there's things that I can do to like lose weight healthier rather than just starving myself or I wish there was someone to tell you that like you're enough"<br>P104: "people I know have developed eating disorders, people that maybe had trauma or shock from a lost one like .. it all stems mentally and I feel like if they would've had mental help, when you neglect your mental health you neglect your physical health, it all connects."<br>P110: "I wouldn't say there was support, it was just really independent."<br><br>*Neglect*<br>P102: "I would actually listen to people because clearly the government doesn't listen to no one he just he does everything what he thinks and I don't I don't trust that, and I don't believe in that so I will, if I was the government, of course I would change everything, I would listen to everybody, even the poor, change a lot of things. (…) I didn't care about that (before COVID) but now I do."<br>P102: "I know I have issues (…) It's just like, I need more support with that um. (…) No one is listening. So I have to shout it out loud so everyone can listen, so yeah."<br>P104: "like in this generation and age there are so many misconceptions about teenagers, especially me as a black female teenager, there's many misconceptions about…being…you know who you are or what you look like"<br><br>*Structural*<br>P103: "it's like sometimes we don't have the best relationship with teachers and like because of the	**B. Needs**<br><br>*Psychotherapy*<br>P103: "if that person is trained to deal with like mental health issues it's way easier to like open up to them and like when I got a counsellor and a mentor like I finally felt like I could talk to someone else about it."<br>P104: "I feel like sometimes psychologically, just like someone to understand and hear me (…) it's hard to kind of like, to speak to somebody without your mind being like oh maybe this person thinks I'm crazy, it would be nice to be finally heard by someone and to talk about your feelings and interests without someone, or your feelings that you felt before, without someone judging you. Because sometimes you just, you just need an ear. You just need someone to listen. I feel like that would really help actually. And it would've helped I think back then if I'd had that I think it would have been way easier to talk about."<br>P104: "and (the therapist) just said what you described does sound like it, and I was like, in that moment like a tear fell on my face because I was like really like it's been confirmed, like maybe he didn't say yeah you have depression but he basically said you're on the way to it,'<br>P106: " (I would need) Like people speaking to me."<br><br>*Relational*<br>P103: "I still have support but it's like from my friends."<br>P105: "I would have like hugs from my friends. (…)I felt like it's a way of comforting myself and comforting other people when it's like hardships and stuff. (…) It just like helps a lot. Because even the simplest thing, like love can do a lot."

For peer review only

past I've been at that school for what like 4 years. (…) there's like background there's a past with some of the teachers and it's not really the best to confide in them"<br>P100: "just like in school, it's very much like,…once in a blue moon ask us if we're alright and that's it."<br>P106: "they don't help they only sit in my class and do nothing when I ask for help then sometimes they listen."	P104: "Um to be honest I actually didn't (reach out) (…) I feel like I made that little depressive state my normal. (…) So I thought- I also didn't reach out I didn't think there was anything wrong with it (…) and it's kind of like, asking for help I'm not really a person that does that. (…)I'm not really a person that goes out for support so that denial was kind of like a comfort place like no you don't need anybody you're fine like you don't need to talk to anybody you're ok, like people who talk to people you don't you don't need that you're not on that level you're ok so I kind of talked myself into not getting help, not speaking to anybody, which caused me to feel really alone."<br><br>*Need for daily life*<br>P107: "I would say maybe more opportunities available. So stuff like sports. Even something like work experience. Sort of that yeah I feel like that would help a lot cause it would help like ease back into things."<br>P107: "I've got very used to the connections I have now cause that's who I rely on for a while, so making new friendships, relationships, has become a lot harder."<br>P100: "About how to maintain a healthy lifestyle even though…circumstances can change like this (fingers snapping)."<br>P105: "But I like to talking about things like that and painting and drawing..it's really nice.It makes me happy. (…) I like doing photography without being restricted."<br>P106: "I go to (a youthclub) and do some art and yeah."

For peer review only

Appendix E
Final Thematic Tables

Superordinate theme 1: Impact of the Covid-19 pandemic on mental health

A. Individual

Self-esteem

P103: "I had my brothers and my mum obviously but like they don't understand obviously, and um like I just really need someone to tell me like you're good enough (…) and like that's all like that's all anyone needs to hear, they just want validation (…) I just want to feel appreciated and like-like I just want to feel stuff like that."

P103: "There was a lot of challenges with like my mental health and like my self-esteem and like I- I still don't but like, I never believed in myself like at all, like at all/ (…) I just like feel like I was never going to be good enough"

P104: "I realised that I had a lot of mental health problems within myself, and I wasn't not really happy, um not very confident, quite insecure"

Lack of motivation

P107: "I feel like because I haven't been doing a lot I've lost the motivation to actually do things."

P107: "Um mentally, I think yeah as I said before my motivation has taken like a big decline and also obviously I feel like bad habits during COVID there's not really much that you can do."

P110: "A lot, uh yeah like laziness, um can't get out of bed as much."

B. Symptoms of mental illness

Anxiety, Depression and Disordered Eating

P100: "Mentally, it affected me both like oh am I going to get COVID next, like got some sort of anxiety maybe with me."

P102: "Umm I've been sad, depressed…It was hard but I had to do it"

P103: "I lost a lot of weight after the pandemic not in the best way. I didn't really eat (…)I never really used to give myself like the attention I deserve and you know with COVID it made it very very bad like my mental health was very bad, I was down there it was so bad. (…) **and it was so such a heavy weight on my heart, it was like it was really hard for me and**

C. Relational

Isolation and individualisation

P103: "(lockdown had a) big impact on my mental health like it made me feel way more alone that like I actually was cause I couldn't go outside obviously. (…) I also felt isolated and also felt like alone and unable to be myself in my own space."

P104: "cause you can have like a million people around you and still feel alone. So I felt like in that moment I felt so alone and...tired and mentally exhausted (…) But I feel like everybody was more out for themselves and not really trying to really help each other (…) so it may have cause some type of division.. **I feel like the community was kind of broken"**

P106: "I felt actually quite sad (…) and angry because I'm at home. And like I just feel too much like energy in the house and you're too hot and you can't do this you can't do that and like you can't go out and that's like what frustrates me"

Positive solitude

P100: "(I enjoyed) having me time. Like being able to do like stuff on my own."

P107: "Um, things I enjoyed the most I'd probably say I got a lot of time to myself."

P104: "during summer like obviously summer it gets dark really late um **I used to go for like walks around my neighbourhood** and obviously I had like no phone nothing so like I would just **like I just used to reflect and it was such a like a peaceful time like after I came back home I thought like..I just felt like free. I felt like there was no **** nothing."**

New connections

P101: "I met lots of new people and got to interact with some people during lockdown"

P104: "(People could) talk to each other, sit down and really think like what is going on in the world."

P104: "I definitely lost friendships and gained friendships.Like I found true friends and I also lost people that I thought were good friends to me, but it just showed that they weren't good friends at all."

For peer review only

**COVID made it all worse (…) so I was losing it, I was losing it."**<br>**P104**: "**I realised a lot of things which kind of drove me into a.. I would say quite a depression in the sense of like I would just staying in my room the whole time, I wouldn't leave it,** stay on my phone but then log off every social media app possible to kind of just block out everything"<br><br>*Trauma*<br>P014: "people passing away drastically like every day the numbers were increasing like I felt (…) there was times you really have to stop and think like this type of like disease this thing is wiping everybody out (…) anybody could have died from it."<br>**P104: "**This is really happening and **nobody was grasping with the fact that people are really dying, (…) like it was sad that so many people couldn't handle COVID they took their lives like it was..it was absolutely devastating."**	P105: "we have gotten closer and we like text my friends during in the middle of class when I was in lockdown and we'd sometimes have video calls."

Superordinate theme 2: Changes in mental health experienced since the end of lockdown	
**A. Self-driven coping**<br><br>*Overthinking*<br>**P102: "I had to do it because if I didn't then how am I supposed to go in life so I literally just looked at the present and just focusing on what I need to do to get through my life."**<br>**P103: "**started **overthinking like I started to like really, evaluate my personality and like who I am as a person** (…) and like my mental health as well cause I've never really thought about mental health at all, ok I'm not ok but there's nothing I can do"<br>P103: "before the pandemic I never really like cared about myself (…)I think that was mainly because there was all this stuff like that was just built up and I never had a chance to release it cause I was always out so I never had to think about it, and COVID made it all worse"<br><br>*Better coping*<br>P102: "I have ADHD (…) so I've set timers to go out for 5 minutes, take my walks. I tell my mum, she has a timer on her phone and I know what time I'll be coming home and (my mum) will have checked on me cause I'll be doing silly stuff so, like stuff like that has changed me	**B. Lifted restrictions**<br><br>*Freedom*<br>**P103: "At the end of lockdown when everything like um the restrictions started lifting I felt like I was finally getting life back."**<br>**P106: "going out in groups without having to get COVID tested themselves before they left the house"**<br>P107: "Cause now that I've finally got the freedom to be out of my house (…) I was out of my house all the time.'<br>**P107: "I took things for granted because obviously something as simple as being outside and going to the shops it's all changed."**<br><br>*Social bonds*<br>**P104: "Seeing my friends for the first time. Like I can't fake that that was like the best feeling cause it's like all these months I haven't seen you and it's like oh my god."**<br>**P107: "when the restrictions first eased like in the summer of 2020. (…) Yeah I feel like then I got a lot closer with a lot of people."**<br>P110: "Good, (friends and family relationships) got even stronger since we could interact again."

For peer review only

and normally it would ne- I would never do that so I'm happy that I've done that. (…)I'm organised better. (…) So that's like COVID, I think COVID actually helped me do all of this stuff, so yeah"<br>**P103: "those little things that I did like I used to go on walks or I just used to like I used to write down my thoughts a lot like it was just like random bits of what I'm feeling throughout the day and like those kind of help you feel better."**<br>**P103: "when I realised it's like there so many different things that I can do to help myself and I just want to find all the right um outlets to project any um anger or aggression any uh sad feelings that I had without causing violence."**<br><br>*Personal growth*<br>**P102**: "I've been depressed I know I've been all down and whatever, **but I think if I didn't have that stage in my life I would not be what who I am right now** (…) I think I've changed for the better cause I used to be like a naughty kid. (…) So now I've like realised like I need to learn, revise,.. like everything changed and I kind of like it because I've never been like this before."<br>P102: "So like, a few years ago I was crazy, I was hanging around with the wrong crowd, and now I've realised like, I've realised I've grown and I have to show my little sister that I can't be like who I am right now, so I had to change my personality just for her (…) cause if it wasn't for her then I would still be behaving bad"<br>**P103**: "And um like I think it strengthened my relationships but **it also strengthened the relationship that I had with myself which is like one of the most important one"**<br>P103: "I think I'm good now, I think if you'd asked- I think if you'd done this interview like a couple months ago (…) I was so stuck, I would have been like I need someone to just talk to."<br>**P109: "Back then I wasn't as social (…) Cause I found a really good series of books and I just kept reading it.(…) It's not that I couldn't be social it's just that I wasn't bothered to, but now I'm like talking a bit more, still reading though"**	

Superordinate theme 3: Support obtained during the Covid-19 pandemic and current needs

For peer review only

A. Support	B. Needs
Lack of support<br>**P100: "Honestly little to none, support yeah"**<br>P103:'I wish I would've know that there's people that I can talk to, or like there's things that I can do to like lose weight healthier rather than just starving myself or I wish there was someone to tell you that like you're enough"<br>P104: "people I know have developed eating disorders, people that maybe had trauma or shock from a lost one like .. it all stems mentally and I feel like if they would've had mental help, when you neglect your mental health you neglect your physical health, it all connects."<br>**P110: "I wouldn't say there was support, it was just really independent."**<br><br>*Neglect*<br>**P102**: "I would actually listen to people because **clearly the government doesn't listen to no one he just he does everything what he thinks and I don't I don't trust that**, and I don't believe in that so I will, if I was the government, of course I would change everything, I would listen to everybody, even the poor, change a lot of things. (…) I didn't care about that (before COVID) but now I do."<br>**P102: "I know I have issues (…)** It's just like**, I need more support with that. (…) No one is listening. So I have to shout it out loud so everyone can listen,** so yeah."<br>**P104: "**like **in this generation and age there are so many misconceptions about teenagers, especially me as a black female teenager, there's many misconceptions about…being…you know who you are or what you look like"**<br><br>*Structural*<br>P103: "it's like sometimes we don't have the best relationship with teachers and like because of the past I've been at that school for what like 4 years. (…) there's like background there's a past with some of the teachers and it's not really the best to confide in them"<br>**P100: "in school, it's very much like,…once in a blue moon ask us if we're alright and that's it."**<br>**P106: "they don't help they only sit in my class and do nothing when I ask for help then sometimes they listen."**	*Psychological*<br>P103: "if that person is trained to deal with like mental health issues it's way easier to like open up to them and like when I got a counsellor and a mentor like I finally felt like I could talk to someone else about it."<br>**P104**: "I feel like sometimes psychologically, just like someone to understand and hear me (…) it's hard to kind of like, to speak to somebody without your mind being like oh maybe this person thinks I'm crazy, it would be nice to be finally heard by someone and to talk about your feelings and interests without someone, or your feelings that you felt before, without someone judging you. Because **sometimes you just, you just need an ear. You just need someone to listen. I feel like that would really help actually. And it would've helped I think back then if I'd had that I think it would have been way easier to talk about."**<br>**P104: "and (the therapist) just said what you described does sound like (depression), and I was like, in that moment like a tear fell on my face** because I was like really like it's been confirmed, like maybe he didn't say yeah you have depression but he basically said you're on the way to it,"<br>**P106: " (I would need) Like people speaking to me."**<br><br>*Relational*<br>P103: "I still have support but it's like from my friends."<br>**P105**: "I would have like **hugs from my friends. I feel like it's a way of comforting myself and comforting other people when it's like hardships and stuff, it just like helps a lot because even the simplest thing, like love can do a lot."**<br>P104: "Um to be honest I actually didn't (reach out) (…) I feel like I made that little depressive state my normal. (…) So I thought- I also didn't reach out I didn't think there was anything wrong with it (…) I'm not really a person that goes out for support so that denial was kind of like a comfort place like no you don't need anybody you're fine like you don't need to talk to anybody you're ok, like people who talk to people you don't you don't need that you're not on that level you're ok so I kind of talked myself into not getting help, not speaking to anybody, which caused me to feel really alone."

	Practical<br>**P107: "**I would say maybe **more opportunities available. So stuff like sports. Even something like work experience. Sort of that yeah I feel like that would help a lot cause it would help like ease back into things (…) making new friendships, relationships, has become a lot harder."**<br>**P100**: "About **how to maintain a healthy lifestyle even though…circumstances can change like this (snaps fingers)."**<br>P105: "But I like to talking about things like that and painting and drawing..it's really nice.It makes me happy. (…) I like doing photography without being restricted."<br>P106: "I go to (a youthclub) and do some art and yeah."

For peer review only

COREQ (COnsolidated criteria for REporting Qualitative research) Checklist

Topic	Item No.	Guide questions/Description	Reported on page No
**Domain 1: Research team and reflexivity**			
Personal characteristics			
Interviewer/facilitator	1	Which author/s conducted the interview or focus group?	Page 8 - Methods
Credentials	2	What were the researcher's credentials? E.g. PhD, MD	Submission system
Occupation	3	What was their occupation at the time of the study?	Submission system
Gender	4	Was the researcher male or female?	N/A
Experience and training	5	What experience or training did the researcher have?	Submission system
Relationship with participants			
Relationship established	6	Was a relationship established prior to study commencement?	Page 8 - Methods
Participant knowledge of the interviewer	7	What did the participants know about the researcher? e.g. personal goals, reasons for doing the research	Page 8 - Methods
Interviewer characteristics	8	What characteristics were reported about the interviewer/facilitator? e.g. Bias, assumptions, reasons and interests in the research topic	N/A
**Domain 2: study design**			
Theoretical framework			
Methodological orientation and theory	9	What methodological orientation was stated to underpin the study? e.g. grounded theory, discourse analysis, ethnography, phenomenology, content analysis	Page 7- Methods
Participant selection			
Sampling	10	How were participants selected? e.g. purposive, convenience, consecutive, snowball	Page 8- Methods
Method of approach	11	How were participants approached? e.g. face-to-face, telephone, mail, email	Page 8- Methods
Sample size	12	How many participants were in the study?	Page 10- Results
Non-participation	13	How many people refused to participate or dropped out? Reasons?	N/A
Setting			
Setting of data collection	14	Where was the data collected? e.g. home, clinic, workplace	Page 8- Methods
Presence of non-participants	15	Was anyone else present besides the participants and researchers?	Page 8- Methods
Description of sample	16	What are the important characteristics of the sample? e.g. demographic data, date	Page 10- Results
Data collection			

For peer review only

Interview guide	17	Were questions, prompts, guides provided by the authors? Was it pilot tested?	Page 9- Methods
Repeat interviews	18	Were repeat interviews carried out? If yes, how many?	N/A
Audio/visual recording	19	Did the research use audio or visual recording to collect the data?	Page 9- Methods
Field notes	20	Were field notes made during and/or after the interview or focus group?	N/A
Duration	21	What was the duration of the interviews or focus group?	Page 8- Methods
Data saturation	22	Was data saturation discussed?	N/A
Transcripts returned	23	Were transcripts returned to participants for comment and/or correction?	N/A
**Domain 3: analysis and findings**			
Data analysis			
Number of data coders	24	How many data coders coded the data?	Page 9- Methods
Description of the coding tree	25	Did authors provide a description of the coding tree?	Appendix C
Derivation of themes	26	Were themes identified in advance or derived from the data?	Page 9- Methods
Software	27	What software, if applicable, was used to manage the data?	N/A
Participant checking	28	Did participants provide feedback on the findings?	N/A
Reporting			
Quotations presented	29	Were participant quotations presented to illustrate the themes / findings? Was each quotation identified? e.g. participant number	Pages 11-15- Results
Data and findings consistent	30	Was there consistency between the data presented and the findings?	Pages 11-15- Results
Clarity of major themes	31	Were major themes clearly presented in the findings?	Pages 11-15- Results
Clarity of minor themes	32	Is there a description of diverse cases or discussion of minor themes?	Pages 11-15- Results

Developed from: Tong A, Sainsbury P, Craig J. Consolidated criteria for reporting qualitative research (COREQ): a 32-item checklist for interviews and focus groups. International Journal for Quality in Health Care. 2007. Volume 19, Number 6: pp. 349 – 357

For peer review only

BMJ Open

The impact of the Covid-19 pandemic on young people from black and mixed-ethnic groups' mental health in West London: A qualitative study.

Journal:	*BMJ Open*
Manuscript ID	bmjopen-2023-071903.R1
Article Type:	Original research
Date Submitted by the Author:	12-Apr-2023
Complete List of Authors:	Lenoir, Romane; University College London, Psychology & Human Development<br>Wong, Keri Ka-Yee ; University College London, Psychology & Human Development
**Primary Subject Heading**:	Public health
Secondary Subject Heading:	Sociology, Public health, Qualitative research
Keywords:	COVID-19, MENTAL HEALTH, QUALITATIVE RESEARCH

SCHOLARONE™
Manuscripts

I, the Submitting Author has the right to grant and does grant on behalf of all authors of the Work (as defined in the below author licence), an exclusive licence and/or a non-exclusive licence for contributions from authors who are: i) UK Crown employees; ii) where BMJ has agreed a CC-BY licence shall apply, and/or iii) in accordance with the terms applicable for US Federal Government officers or employees acting as part of their official duties; on a worldwide, perpetual, irrevocable, royalty-free basis to BMJ Publishing Group Ltd ("BMJ") its licensees and where the relevant Journal is co-owned by BMJ to the co-owners of the Journal, to publish the Work in this journal and any other BMJ products and to exploit all rights, as set out in our licence.

The Submitting Author accepts and understands that any supply made under these terms is made by BMJ to the Submitting Author unless you are acting as an employee on behalf of your employer or a postgraduate student of an affiliated institution which is paying any applicable article publishing charge ("APC") for Open Access articles. Where the Submitting Author wishes to make the Work available on an Open Access basis (and intends to pay the relevant APC), the terms of reuse of such Open Access shall be governed by a Creative Commons licence – details of these licences and which Creative Commons licence will apply to this Work are set out in our licence referred to above.

Other than as permitted in any relevant BMJ Author's Self Archiving Policies, I confirm this Work has not been accepted for publication elsewhere, is not being considered for publication elsewhere and does not duplicate material already published. I confirm all authors consent to publication of this Work and authorise the granting of this licence.

For peer review only

The impact of the Covid-19 pandemic on young people from black and mixed-ethnic groups' mental health in West London: A qualitative study.

Author: Romane Lenoir, BSc Psychology with Education Alumni, Department of Psychology and Human Development, University College London, London, United-Kingdom

Co-author: Dr. Keri Ka-Yee Wong, Associate Professor of Psychology, Department of Psychology and Human Development, University College London, London, United-Kingdom

Corresponding author: Dr. Keri Ka-Yee Wong, Department of Psychology and Human Development, 25 Woburn Square, London WC1H 0AA, keri.wong@ucl.ac.uk, Tel: (+44) 78997-98350

Key words: Covid-19; Mental health; Qualitative Research

Word count: 4362 words

For peer review only

Abstract

Objectives

The Covid-19 pandemic has disproportionately impacted vulnerable groups' physical and mental health, especially young people and minority ethnic groups, yet little is known about the crux of their experiences and what support they would like. To address this gap, this qualitative study aims to uncover the effect of the Covid-19 outbreak on young people with ethnic minority backgrounds' mental health, how this changed since the end of lockdown and what support they need to cope with these issues.

Setting and Participants

Ten 15-minute in-person semi-structured interviews were conducted with young people aged 12 to 17 years old from black and mixed-ethnic groups who regularly attend a community centre in West London.

Results

Through Interpretative Phenomenological Analysis, results indicated that the participants' mental health was negatively impacted by the Covid-19 pandemic, with feelings of loneliness being the most common experience. However, positive effects were concurrently observed including improved wellbeing and better coping strategies post-lockdown, which is a testament to the young people's resilience. That said, it is clear that young people from minority ethnic backgrounds lacked support during the Covid-19 pandemic and would now need psychological, practical and relational assistance to cope with these challenges.

Conclusions

Whilst future studies would benefit from a larger ethnically-diverse sample, this is a start. Study findings have the potential to inform future government policies around mental health support

For peer review only

and access for young people from ethnic minority groups, notably prioritising support for grassroots initiatives during times of crisis.

Strengths and limitations

- This qualitative interview study during Covid-19 gives voice to the experiences of young people from black and mixed-ethnic backgrounds in the UK
- The in-person quality of the interviews helped build rapport between the researcher and the young people and sharing of sensitive issues around mental health access and support, increasing the results' validity
- This is a convenient sample, with girls and those aged 15 years and above being disproportionately represented in our data as they provided most of the answers.
- The small sample size and lack of ethnic diversity limits the generalisability of the study to individuals from other ethnic minority groups.

For peer review only

The severe acute respiratory syndrome SARS-CoV-2 (Covid-19) coronavirus, infecting over 600 million people and claiming more than six million lives and counting, has triggered unprecedented economic and social disruptions worldwide [1, 2]. Whilst everyone has been impacted, certain groups have been disproportionately affected [3], including individuals from ethnic minority groups [4]. Mortality rates in the United Kingdom (UK) between March and April 2020 for instance, were two and three times higher for individuals from minority ethnic groups than for ethnically white groups [5]. In April 2021, the UK Intensive Care National Audit and Research Centre reported that ethnic minorities represented 34% of the patients in intensive care units despite only constituting 11% of the UK population [6]. These alarming trends of physical vulnerability for individuals from ethnic minorities during Covid-19 begs the question of why this is the case, and what support might look like for this group.

An ecological explanation can be offered to elucidate these patterns. Indeed, a holistic consideration the dynamic interplay between an individual and their environment [7, 8] suggests that situational risk factors such as multi-generational living conditions and environmental adversity - most notably associated with individuals from minority ethnic groups' living conditions - may increase ethnic minority groups' vulnerability to Covid-19 [9]. Additionally, they are overrepresented in socially disadvantaged neighbourhoods, with white

For peer review only

individuals only representing 8,7% of the population in the 10% most income-deprived neighbourhoods in the UK in 2016 [10]. These are characterised by a limited capacity to socially distance, resulting in higher Covid-19 infection and death rates [11]. Ethnic minority individuals also occupy more key-worker positions, as they constituted over 20% of the NHS workforce whilst representing under 15% of the working-age population in the UK in 2019 [12]. This overall disadvantage in the labour market exposes them to greater risk during the Covid-19 pandemic [13]. Finally, it may also be the case that social networks being racially differentiated increases intra-community transmission for ethnic minority groups [14]. The Covid-19 pandemic thus not only exacerbated pre-existing health inequalities for ethnic minority groups in the UK, but deepened socio-economic ones, the impact of which must be investigated [15].

Studies of the effect of the Covid-19 pandemic on ethnic minority groups' day-to-day life stressors have been particularly informative. Mind, a UK-based mental health charity, surveyed adults' experiences during the pandemic and found that individuals from minority ethnic groups experienced higher levels of everyday distress [16]. 30% of adults from ethnic minorities reported that their mental health was affected by housing issues, compared to 23% of whites. Similar rates were reported for troubles with employment (61% against 51% for white individuals), finances (52% against 45%) and physical health support (39% versus 29%)

For peer review only

[16]. However, ethnic minorities notably represented less than 5% of the study's sample. Significantly, within individuals from ethnic minority groups, the pandemic seemingly impacted the mental health of individuals from black and mixed ethnicities the most [17]. Additionally, individuals from ethnic minorities in the UK were more exposed to food insecurity during Covid, and had a higher reliance on food banks than their white counterparts [18]. Finally, long-term inequalities in socioeconomic opportunities and a history of mistrust towards governing entities have cultivated feelings of alienation amongst minority ethnic groups [19, 20]. The Covid-19 pandemic deepened this rift, reinforcing feelings of social isolation [14, 21].

Young people from ethnic minority groups have also suffered during Covid-19, being deprived of structures essential to their socio-emotional development, which caused increased psychological distress [22-24]. Yet, young people have also demonstrated considerable resilience, most importantly through protective family environments and increased self-awareness [25, 26]. Documenting these experiences is crucial as young peoples' voices are too often missing from academic research on Covid-19 and mental health due to difficulties in recruitment and access. Their scarcity makes developing support mechanisms for young people rather difficult. Also, as a majority of studies are quantitative in nature [27, 28] which, albeit

For peer review only

informative, does not create a space for young people to voice their needs and allow them to feel heard. Not least, young people from minority ethnic groups are at heightened risk of experiencing distress during the Covid-19 pandemic and therefore deserving to be actively involved in and listened to in this research.

Existing quantitative studies on the mental health of individuals from minority ethnic backgrounds are limited in that they very rarely examine the experiences underlying the patterns they observe. To date, very few qualitative studies have focused on the impact of Covid-19 on minority ethnic individuals, and even less on young people. The first is Mahmood et al.'s paper [29], which interviewed 19 ethnic minority community leaders between October and November 2020 about the Covid-19 pandemic's impact on their communities. Participants spoke about the financial, social and physical consequences of the pandemic, the latter being subjectively justified by historical and structural disparities. Results indicated that mental health was a fundamental concern, as the participants invoked their group's usual tight-knit nature to illustrate the extent of lockdown's impact on their wellbeing [29]. Secondly, Burgess et al. investigated ethnicity and mental health during Covid-19, with a highly relevant target demographic of young people, with 87% of black participants [30]. Through four focus groups, the study identified deteriorating mental health and experiences of racism, alongside more

For peer review only

encouraging patterns of resilience and growth through self-care. Other qualitative studies on young people's mental health during the pandemic, although not specifically focusing on ethnic minorities, similarly note worrying patterns of worsening wellbeing, and specifically increased anxiety, racism and stigma [31, 32]. The scarcity of literature solely concentrating on first-hand experiences of minority ethnic groups throughout the Covid-19 pandemic highlights the need for more qualitative studies to truly understand how this pandemic has impacted this group.

With the pinnacle of the pandemic now behind us, we must identify what the barriers to mental health support might be and how can we best support young people now rather than later. First, recognising that young people's struggles are legitimate and encouraging them to seek support is essential [16]. Second, the structural and interpersonal obstacles young people from ethnic minority groups face when seeking mental health support are a key factor driving low incidences of access to support [33]. Within the Mind survey, half of the young people revealed that difficulties in accessing support made their mental health worse [16]. Lower English proficiency has also been associated with reduced use of mental health resources, with a significantly steeper effect within minority ethnic groups [34]. The low intelligibility of online information surrounding Covid-19 in the UK revealed a striking lack of appropriate education materials available to these groups [35]. Intra-community stigma, most significant for black individuals in the UK, is also a long-standing impeding change in mental wellbeing [36]. Further isolating young people, these obstacles exacerbate their distrust, and create a reluctance and stigma to seeking mental health support [29]. The reality is that young people's

For peer review only

mental health as a whole, but most significantly those from ethnic minority groups, has not only been worsening since the start of the Covid-19 pandemic but is also poorly assisted [29, 37, 38].

The current qualitative interview study aims to identify the impact of the Covid-19 pandemic on young people from minority ethnic groups and the types of support they might need. By giving voice to communities less heard from, we hope to inspire larger-scale research and develop tailored mental health support for black and mixed ethnic minority communities.

Methods

Participants and Ethics

The ten participants were recruited via convenience sampling from a community centre in West London. The current pre-registered CopeWell Study [39] (https://osf.io/jcak7/), funded by UK Research and Innovation, aimed to understand the impact of the Covid-19 pandemic on young people's mental and physical health and to co-create appropriate life-skill workshops to enable young people from black and mixed-ethnic backgrounds to better cope with the pandemic. The present study focuses on the initial interviews on the impact and challenges of Covid-19 on young people's lives. Ethical approval was granted by the UCL Institute of Education Ethics Committee (REC 1558) on 29 October 2021. A pilot study was conducted for the interview schedule, confirming that the questions' content and order were cohesive

For peer review only

(Appendix A). The prods' usefulness in allowing the participants to elaborate was assessed, familiarising the researcher with their navigation. Parents received and completed an information sheet and consent form before the study commenced. Similarly, participants' written consent was obtained at the time of the interview. They were also reminded of their right to withdraw or omit to answer at any point. Confidentiality was ensured and potential questions were answered. The study was then conducted from the 17th of October 2021 to the 8th of December 2021, where participants took part in 15-minute one-on-one semi-structured interviews (*M* = 14:06 min) with 14 open-ended questions. They were thanked and debriefed post-interview and received a £30 gift voucher as an honorarium. We built rapport with the youth workers and young people before conducting the interviews at the community centre (a familiar and safe environment). Covid-19 safety measures (e.g. masking, distancing, well-ventilated space) were applied where necessary. The participants' answers were recorded via audio software to maximise accuracy and were subsequently transcribed by R.L. [40].

Materials

The initial pilot interview schedule contained 14 open-ended questions, with prods guiding the discussion according to the participants' reactivity and answers, and was designed by K.W.. It was subsequently amended in consultation with R.L. to form the final version

For peer review only

(Appendix B). Questions progressed from items about the Covid-19 pandemic and its impact on the participants' mental health, to potential changes in these experiences. This was followed by the kind of support they received and needed to cope with these struggles.

Data analysis

The analysis was conducted following Smith's Interpretative Phenomenological Analysis (IPA) [41]. IPA is relevant for this study as it draws focus on the young people's points of view and the meanings they ascribe to their situation. The explicitly idiographic nature of this analytical method allowed for the participants' lived experiences to be thoroughly probed into [42]. Smith and Shinebourne's six steps analysis for IPA was followed [43], resulting in the delineation of three *superordinate themes*, each containing *themes* and *subthemes*. Through an iterative process of reading and re-reading the transcripts, the researcher (R.L.) made initial annotations and colour-coded quotes according to the study aims they pertained to. Coding was continued until saturation was reached (70% of transcripts).

The most frequent and significant patterns were then clustered together (Appendix C) and agreed with K.W. through discussions. The thematic relationship was summarised in an initial table with subthemes and sample quotes (Appendix D). IPA, as an iterative process, allowed the researcher to continuously revise themes and quotes, concomitantly ensuring the

For peer review only

integrity of the participants' accounts was preserved [43]. Applying this not only preserved the interpretative role of the researcher in IPA, but buffered researcher bias risks [44, 45]. The final thematic relationship is presented in a table (Appendix E).

Patient and public involvement

Patients or the public were not involved in the design, or conduct, or reporting or dissemination plans of our research.

Results

A total of ten young people aged 12 to 17 participated in the study ($M = 14.3$, $SD = 1.3$ years, female = 70%). Participants self-identified from a list provided in the questionnaire with the following ethnic groups: black or black African ($n = 4$), black or black Caribbean ($n = 1$), any other black background ($n = 1$), white and black African ($n = 2$) and white and black Caribbean ($n = 1$). One participant preferred not to say. Three superordinate themes emerged, namely overall declining patterns of mental health during the Covid-19 pandemic, improvements post-lockdown, and needs moving forward.

Superordinate theme one: Impact of the Covid-19 pandemic on mental health

Individual

For peer review only

As a result of being required to stay indoors, 30% of the young people described suffering from decreased self-esteem during the Covid-19 pandemic. Underlying insecurities manifested themselves through self-doubt and low confidence. Additionally, 20% of the young people spoke about the monotony of life during lockdown causing them to lose their motivation and ambition (table 1).

Symptoms of mental illness

Participants' previous apprehensions about life and patterns of low mood were intensified by the Covid-19 pandemic, as 60% of them recounted symptoms of anxiety, depression and disordered eating. Lockdown freed up time for overthinking and the development of unhealthy habits of thoughts and behaviour. These were heightened by anxieties surrounding the illness itself for 20% of the participants, through exposure to a number of traumatic events such as suicides and climbing death rates (table 1).

Relational

The isolation provoked by lockdown shifted the participants' rapport to others, negatively impacting 90% of the young people's mental health. Loneliness and fears arising from the speed and risk of virus transmission fuelled tensions and individualisation trends amongst ethnic minority groups. However, solitude was also perceived as restorative, as 30%

For peer review only

of the young people utilised this time to reflect and enjoy time on their own as a break from an otherwise hectic life. Interestingly, 50% of the participants also reported that the Covid-19 pandemic not only allowed them to reinforce current connections and friendships but to develop new ones (table 1).

Table 1: Impact of the Covid-19 pandemic on mental health

**Theme**	**Subtheme**	**Illustrative quotes**
Individual impact	Self-esteem	P103: 'There was a lot of challenges with like my mental health and like my self-esteem and like I- I still don't but like, I never believed in myself like at all, like at all/ (…) I just like feel like I was never going to be good enough' (age 15)<br>P104: 'I realised that I had a lot of mental health problems within myself, and I wasn't not really happy, um not very confident, quite insecure' (age 16)
	Lack of motivation	P107: 'I feel like because I haven't been doing a lot I've lost the motivation to actually do things.' (age 15)<br>P107: 'Um mentally, I think yeah as I said before my motivation has taken like a big decline and also obviously I feel like bad habits during COVID there's not really much that you can do.' (age 15)<br>P110: 'A lot, uh yeah like laziness, um can't get out of bed as much.' (age 15)
Symptoms of mental illness	Anxiety, Depression and Disordered Eating	P100: 'Mentally, it affected me both like oh am I going to get COVID next, like got some sort of anxiety' (age 15)<br>P103: 'I lost a lot of weight after the pandemic not in the best way. I didn't really eat (…)I never really used to give myself like the attention I deserve and you know with COVID it made it very very bad like my mental health was very bad, **(…)** and it was so such a heavy weight on my heart, it was like it was really hard for me and COVID made it all worse (…) so I was losing it, I was losing it.' (age 15)

For peer review only

		P104: 'I realised a lot of things which kind of drove me into a.. I would say quite a depression in the sense of like I would just staying in my room the whole time, I wouldn't leave it' (age 16)
	Trauma	P104: 'nobody was grasping with the fact that people are really dying, (…) like it was sad that so many people couldn't handle COVID they took their lives like it was..it was absolutely devastating.' (age 16)
Relational	Isolation and individualisation	P103: '(lockdown had a) big impact on my mental health like it made me feel way more alone that like I actually was cause I couldn't go outside obviously. (…) I also felt isolated and also felt like alone and unable to be myself in my own space.' (age 15)<br>P104: 'cause you can have like a million people around you and still feel alone. So I felt like in that moment I felt so alone and...tired and mentally exhausted (…) But I feel like everybody was more out for themselves and not really trying to really help each other (…).. I feel like the community was kind of broken.' (age 16)
	Positive solitude	P100: '(I enjoyed) having me time. Like being able to do like stuff on my own.' (age 15)<br>P107: 'Um, things I enjoyed the most I'd probably say I got a lot of time to myself.' (age 15)<br>P104: 'I used to go for like walks around my neighbourhood (…) like I just used to reflect and it was such a like a peaceful time like after I came back home I thought like..I just felt like free. I felt like there was no **** nothing.' (age 16)
	New connections	P101: 'I met lots of new people and got to interact with some people during lockdown' (age 12)<br>P104: '(People could) talk to each other, sit down and really think like what is going on in the world.' (age 16)<br>P104: 'I definitely lost friendships and gained friendships.Like I found true friends and I also lost people that I thought were good friends to me' (age 16)

Superordinate theme two: Changes in mental health since the end of lockdown

Autonomous adaptive coping skills

For peer review only

Being left alone with their thoughts led the young people to demonstrate great levels of resilience in the face of adversity. Indeed, 30% of them reported introspective self-evaluations and the development of adaptive self-regulating mechanisms. 50% of the participants observed growth in themselves, transpiring into their relationships with others. This demonstrates great resilience and resourcefulness amongst the young people (table 2).

Lifted restrictions

Lockdown ending meant newfound freedom, which 50% of the participants experienced as positively impacting their mental health. Restrictions being lifted allowed them to enjoy normal life again by being outside and regaining individual freedoms. Additionally, social bonds were reinstated, further benefitting their mental health (table 2).

Table 2: Changes in mental health since the end of lockdown

**Theme**	**Subtheme**	**Illustrative quotes**
Autonomous adaptive coping skills	Overthinking	P102: 'I had to do it because if I didn't then how am I supposed to go in life so I literally just looked at the present and just focusing on what I need to do to get through my life.' (age 15)<br>P103: 'overthinking like I started to like really, evaluate my personality and like who I am as a person' (age 15)
	Better coping	P103: 'those little things that I did like I used to go on walks or I just used to like I used to write down my thoughts a lot like it was just like random bits of what I'm feeling throughout the day and like those kind of help you feel better.' (age 15)<br>P103: 'when I realised it's like there so many different things that I can do to help myself and I just want to find all the right

For peer review only

		um outlets to project any um anger or aggression any uh sad feelings that I had without causing violence.' (age 15)
	Personal growth	P102: 'I've been depressed I know I've been all down and whatever, but I think if I didn't have that stage in my life I would not be what who I am right now' (age 15)<br>P103: 'And um like I think it strengthened my relationships but it also strengthened the relationship that I had with myself which is like one of the most important one' (age 15)<br>P109:'Back then I wasn't as social (…) Cause I found a really good series of books and I just kept reading it.(…) It's not that I couldn't be social it's just that I wasn't bothered to, but now I'm like talking a bit more, still reading though.' (age 14)
Lifted restrictions	Freedom	P103: 'At the end of lockdown when everything like um the restrictions started lifting I felt like I was finally getting life back.' (age 15)<br>P106: 'going out in groups without having to get COVID tested themselves before they left the house' (age 13)<br>P107: 'I took things for granted because obviously something as simple as being outside and going to the shops it's all changed.' (age 15)
	Social bonds	P104: 'Seeing my friends for the first time. Like I can't fake that that was like the best feeling cause it's like all these months I haven't seen you and it's like oh my god.' (age 16)<br>P107: 'when the restrictions first eased like in the summer of 2020. (…) Yeah I feel like then I got a lot closer with a lot of people.' (age 15)

Superordinate theme three: Limited support obtained during the Covid-19 pandemic and current needs

Support

70% of the young people deplored a lack of support during the Covid-19 pandemic. In terms of structural support, 50% of the participants were critical of the academic help they were provided with. Additionally, 30% of them shared feeling neglected by society and governing bodies, evidenced by a deep mistrust in national institutions and strong feelings about being overlooked, ignored and misunderstood as young people from black and mixed-ethnic minority backgrounds (table 3).

Needs

The young people requested various forms of support moving forward, the largest being psychological support (50%). In response to social bonds being challenged during the Covid-19 pandemic, 40% of the participants also requested relational support. With socialising being key to their wellbeing, support in this area would benefit them greatly. Finally, practical support with professional opportunities and maintaining a healthy routine, habits which were abruptly perturbed by the Covid-19 pandemic, was considered useful by 50% of the young people (table 3).

Table 3: Limited support obtained during the Covid-19 pandemic and current needs

Theme	Subtheme	Quotes and illustrations
Support	Lack of support	P100: 'Honestly little to none, support yeah' (age 15)<br>P110: 'I wouldn't say there was support, it was just really independent.' (age 15)

For peer review only

	Neglect	P102: 'I would actually listen to people because clearly the government doesn't listen to no one he just he does everything what he thinks and I don't I don't trust that' (age 15)<br>P102: 'I know I have issues (…) It's just like, I need more support with that. (…) No one is listening. So I have to shout it out loud so everyone can listen' (age 15)<br>P104: 'in this generation and age there are so many misconceptions about teenagers, especially me as a black female teenager, there's many misconceptions about…being…you know who you are or what you look like' (age 16)
	Structural	P100: 'in school, it's very much like,…once in a blue moon ask us if we're alright and that's it.' (age 15)<br>P106: 'they don't help they only sit in my class and do nothing when I ask for help then sometimes they listen.' (age 13)
Needs	Psychological	P104: 'sometimes you just, you just need an ear. You just need someone to listen. I feel like that would really help actually. And it would've helped I think back then if I'd had that I think it would have been way easier to talk about.' (age 16)<br>P104: 'and (the therapist) just said what you described does sound like (depression), and I was like, in that moment like a tear fell on my face' (age 16)<br>P106: ' (I would need) Like people speaking to me.' (age 13)
	Relational	P105: 'I would have like hugs from my friends. I feel like it's a way of comforting myself and comforting other people when it's like hardships and stuff, it just like helps a lot because even the simplest thing, like love can do a lot.' (age 13)
	Practical	P107: 'I would say maybe more opportunities available. So stuff like sports. Even something like work experience. Sort of that yeah I feel like that would help a lot cause it would help like ease back into things (…) making new friendships, relationships, has become a lot harder.' (age 15)<br>P100: 'About how to maintain a healthy lifestyle even though…circumstances can change like this (snaps fingers).' (age 15)

Discussion

For peer review only

This study investigated the impact of the Covid-19 pandemic on the mental health of young people from black and mixed-ethnic groups, how this has changed since the end of lockdown and the support that they may need to cope with these issues. Results indicated that young people's mental health was both negatively and positively impacted by the Covid-19 pandemic. However, their mental wellbeing seemingly improved after the peak of the Covid-19 pandemic, due to adaptive coping mechanisms being developed and lockdown ending. Finally, the young people deplored a lack of support during the Covid-19 outbreak and were critical of the structural support they did receive. They asked for psychological, relational and practical support moving forward.

Firstly, participants reported experiencing the negative consequences of the Covid-19 pandemic on a personal level, in their relationships and on their mental health. This reinforces past research evidencing deteriorating mental health in young people from minority ethnic groups, both quantitatively [24, 27, 46] and, to a lesser extent, qualitatively [25, 30]. More specifically, such patterns have been observed in the ethnic minority groups prevalent in our study. Mixed ethnic young people seemed to have most challenges with mental health and social connections, whilst black young people struggled with academics [47]. The present study nonetheless also demonstrated an improvement in the participants' mental health post-

For peer review only

pandemic (Nov-Dec 2021), echoing wider studies reflecting this [25, 26, 29]. McKinlay et al.'s results were not ethnic-specific [25], increasing the present study's relevance in focusing on the experiences of young people from black and mixed-ethnic minority groups. Additionally, the measures utilised in Penner et al.'s research were taken before the studied area's Covid-19 peak [26], limiting their validity. The present study however interviewed young people who have experienced multiple lockdowns, strengthening the results' validity. However, reports of past experiences are subject to recall bias and individuals may overestimate positive affect retrospectively as a coping strategy, which potentially reduces the internal validity of the present study's results [48, 49].

The improvements in mental health post-lockdown found in our study, demonstrate great resilience among the young people. This is supported by research evidencing how young black men in particular demonstrate great resilience when faced with hardship by drawing on different sources of social capital [50]. However, our results contrast with other literature suggesting that, although observing a general amelioration in mental health symptoms after lockdown ended, scores remained higher than pre-pandemic [51]. This implies a long-lasting detrimental impact which was not observed in the present group of young people. This is potentially due to measurement differences as most quantitative studies measure changes in

For peer review only

scores whilst qualitative ones focus on the most salient experience for the individual. Additionally, research suggests that although improvements in mental health after lockdown were observed amongst most young people, they did not manifest in at-risk youth [52]. The latter study's selection criteria for at-risk youth was household income, which although partially overlapping with ethnic minority, remains a distinctive non-interchangeable demographic [53]. This is not a criteria we used for our young people.

Finally, the young people deplored a lack of support during the Covid-19 outbreak and were critical of the structural support they received, echoing previous studies deploring poorly adapted mental health resources for individuals from ethnic minorities [16, 35]. Indeed, these highlight issues of stigma and the ways in which non-culturally-sensitive forms of therapy and imbalanced power dynamics affect the therapeutic relationship, as reported amongst black participants [54]. This creates further reluctance to seek help for this particular ethnic group. Additionally, participants expressed feeling neglected by society and governing entities, mirroring previous reports of alienation amongst individuals from minority ethnic groups [14, 21]. The present study's qualitative methodology thus effectively contributed to solidifying the existing body of literature around this theme. Study participants did not mention English proficiency as a barrier to them accessing mental health support, as was suggested by Sentell

For peer review only

et al.'s research [34]. However, the participants are native English speakers, suggesting this obstacle may not apply to individuals from all ethnic minority groups. Similarly, stigma around mental health was not explicitly identified as an obstacle to mental health access contrary to Mahmood et al.'s findings [29]. In fact, study participants advocated for mental health awareness and its importance for public health discussion. One reason for this discrepancy in findings could be because participants in Mahmood et al.'s study were adult community leaders [29], a significantly older generation compared to the young people in our study, potentially reflecting a generational difference in perceptions of mental health, stigma, and willingness to discuss health issues. Fortunately, this might indicate that young people from black and mixed ethnic groups simply need more exposure to mental health knowledge overall and opportunities for open, non-judgmental discussions.

Strengths and limitations

A key strength this study possesses is its qualitative nature and in-person data collection method. By allowing young people to elaborate on their personal experiences, this study captured subtleties that wide-ranging mental health scales may have overlooked [55]. Additionally, as Covid-19 studies were mostly run mid-pandemic, sanitary restrictions compelled them to be conducted remotely [30]. Although online and anonymised methods for

For peer review only

interviewing have been associated with lower social desirability, they increase the risk of measurement error and misclassification [27, 56]. Virtual environments curb the effectiveness of non-verbal cues, affecting rapport which is vital when conducting sensitive research with vulnerable populations [57, 58]. The present study's in-person collection method of qualitative data enabled the researcher to invest time and effort in building rapport with the participants, increasing the results' validity and providing the participants with a valuable space to feel heard.

However, the wide range and variation in the study participants' age represents a key limitation. Indeed, participants ranged from 12-to-17 years-old. The convenience sampling method utilised gave the researcher no control over the specific ages of the young people included in the study. This is relevant as young people's experiences from late childhood to late adolescence vary widely [59]. During this period, they go through a time of significant personal development, within which age is a highly influential factor [22]. Their age, and associated experiences, thereby inevitably introduce bias in the stories they told and the needs they voiced. That said, we recognise that our results do not reflect answers from the full age sample, containing mostly quotes from participants aged 15 and above, and encourage future research to further this endeavour with a larger representative sample. Additionally, we note that we do not elucidate on gendered patterns, as we did not predict that our sample would be

For peer review only

imbalanced. However we acknowledge that there is a large body of existing evidence investigating this particular factor and its interaction with ethnicity and mental health across the pandemic [17, 47, 60]. The results' generalisability to young people from minority ethnic groups overall is therefore low, nonetheless capturing relevant perspectives from young people of ethnic minority groups.

Implications for practice and research

Young people's needs - psychological, relational and practical- encapsulated in this study resonate with current initiatives and policies being put into place by organisations, but should be sustained post-pandemic. Initiatives such as telehealth for young people from ethnic minority backgrounds [61] and the youth Covid-19 Support Fund [62] allowed for the continued provision of support and safe spaces for young people during the Covid-19 pandemic. However, as black young people have reportedly struggled most with remote communications amongst ethnic minority groups, there is a striking need for targeted support [47]. Unfortunately, schemes supporting ethnic minority groups were highly hindered by lockdown and social distancing, limiting their scope for action [63]. Importantly, young people's involvement in the creation of frameworks that will impact them was identified by the United Nations Convention on the Rights of the Child as a fundamental human right [64]. Young

For peer review only

people's voices and needs for support identified in the present study already had wide ranging albeit short-term impact across stakeholders involved. Basing the workshops on young people's voices allowed previously unnoticed needs to be addressed, such as relational support, mental health, and practical help to enter the professional world. The charity – who engages with the young people on a weekly basis – gained a deeper understanding of the young peoples' needs and expanded the scope of possible initiatives beyond psychological support [16]. Student researchers involved also gained first-hand experience with a full timeline of the research process and insight into the widely differing experiences of individuals throughout the Covid-19 pandemic, which adds to the existing literature [3]. By bringing previously unnoticed voices into focus, in cooperation with the CopeWell project [39], this study exemplifies how community-based research can be a means to advocate for greater policy developments promoting racial justice in response to the Covid-19 outbreak [65]. Crucially, this study highlights the importance of developing grassroots support that is sustained through policy and practice for young people from black and mixed-ethnic groups in the aftermath of the Covid-19 pandemic and beyond.

Future research should proactively identify those unanswered needs and provide support, particularly for black and mixed ethnic young people through local co-produced

For peer review only

solutions. Specifically, prior research solely focuses on assessing attitudes towards available support or lack thereof, which may be less adaptable to future changes and needs. Conversely, our qualitative interviews provided the young people with a platform to share the assistance they would want. Through the process of giving voice to young people, they reported feeling heard and seen [66], giving strength to qualitative methods of inquiry considering long-term perspectives. However, this study is not without limitation. Participants' ethnicities were limited to black and mixed-ethnic groups and a wide age range, offering an incomplete picture of young people from ethnic minority groups' mental health in the Covid-19 context [67]. This highlights the need for future research to include varied samples of young people from minority ethnic groups in their qualitative investigations, yielding results with higher validity. Alternatively, age groups may be considered individually to tailor results and subsequent interventions to potential corresponding variations in needs. Studies may also extend to wider geographical areas, investigating cultural variations in the impact of the Covid-19 pandemic on young people from minority ethnic backgrounds, raising awareness towards this hitherto neglected issue on a larger scale. Finally, this study evidenced that the Covid-19 outbreak was cruelly reflective of social disparities in the UK, warranting the need to probe into other

For peer review only

vulnerable groups' mental health in this context, namely individuals with disabilities or lower socio-economic statuses.

Conclusion

This qualitative interview study of young people from black and mixed-ethnic backgrounds during the Covid-19 pandemic has highlighted important challenges and barriers to mental health access and young people's need for support with mental health during Covid, as assessed in October to December 2021. Unexpectedly, study findings also highlighted young people's resilience and lessons learned when faced with global world challenges, such as coping mechanisms and the establishment of new relational connections. The study yielded an in-depth qualitative understanding of black and mixed minority ethnic young people's needs and support together with input from charity workers, student co-researchers, and academics. Future research studies with larger, more representative samples can enhance our understanding of the hardships vulnerable groups face in such unprecedent times, the strength and resilience they have, and can inform the development of support specific to the unjust burden they carry.

For peer review only

Acknowledgments I would like to extend my sincere thanks to my supervisor K.W. for her advice and continuous support, as well as to the young people and social workers who took part in this study for their time and contribution.

Contributors statement: R.L. was involved with the data collection, analysing and interpretation of data, drafting, writing, reviewing, and final approval of the article. K.W. was involved with the conception of the research question, data collection, drafting, writing, reviewing, management of the project, supervision of the work, and final approval of the article. R.L. and K.W. agree to be accountable for all aspects of the work in ensuring that questions related to the accuracy or integrity of any part of the work are appropriately investigated and resolved.

Funding This work was supported by UK Research and Innovation, from UCL's HEIF Knowledge Exchange and Innovation Fund 2021-2022.

Competing interests None declared.

Patient consent for publication Not applicable.

Ethics approval Ethical approval was obtained from the UCL Institute of Education Ethics Committee (REC 1558). Informed consent was received from all participant and their parents.

For peer review only

Provenance and peer review Not commissioned; externally peer-reviewed

Data availability statement Data are available in a public, open access repository, OSF. https://osf.io/jcak7/.

Supplemental material This content has been supplied by the author(s). It has not been vetted by BMJ Publishing Group Limited (BMJ) and may not have been peer-reviewed. Any opinions or recommendations discussed are solely those of the author(s) and are not endorsed by BMJ. BMJ disclaims all liability and responsibility arising from any reliance placed on the content. Where the content includes any translated material, BMJ does not warrant the accuracy and reliability of the translations (including but not limited to local regulations, clinical guidelines, terminology, drug names and drug dosages), and is not responsible for any error and/or omissions arising from translation and adaptation or otherwise.

Open access This is an open access article distributed in accordance with the Creative Commons Attribution Non Commercial (CC BY-NC 4.0) license, which permits others to distribute, remix, adapt, build upon this work non-commercially, and license their derivate works on different terms, provided the original work is properly cited, appropriate credit is given, any changes made indicated and the use is non-commercial. See: http://creativecommons.org/licenses/by-nc/4.0/.

For peer review only

ORCID ID R.L.: 0000-0001-6277-8203. K.W.: 0000-0002-2962-8438

For peer review only

References

1 Tisdell, C. A. (2020). Economic, social and political issues raised by the Covid-19 pandemic. *Economic Analysis and Policy*, *68*, 17-28. https://doi.org/10.1016/j.eap.2020.08.002

2 World Health Organization (2020). Coronavirus (Covid-19) Dashboard. *World Health Organization*. Available online: https://covid19.who.int/

3 Wong, K. K.-Y., Loke, K., & Melville, K. M. K. L. (2022). Reflections, Resilience, and Recovery: A qualitative study of COVID-19's impact on an international adult population's mental health and priorities for support. *UCL Open: Environment Preprint*

4 Tai, D. B. G., Shah, A., Doubeni, C. A., Sia, I. G., & Wieland, M. L. (2021). The disproportionate impact of COVID-19 on racial and ethnic minorities in the United States. *Clinical Infectious Diseases*, 72(4), 703-706. https://doi.org/10.1093/cid/ciaa815

5 Aldridge, R. W., Lewer, D., Katikireddi, S. V., Mathur, R., Pathak, N., Burns, R Fragaszy, E.B., Johnson, A.M., Devakumar, D., Abubakar, I. and Hayward, A. (2020). Black, Asian and Minority Ethnic groups in England are at increased risk of

death from Covid-19: Indirect standardisation of NHS mortality data. *Wellcome Open Research*, *5*. https://dx.doi.org/10.12688%2Fwellcomeopenres.15922.2

6 Intensive Care National Audit and Research Centre (ICNARC). (2022). ICNARC Covid-19 report on critical care. *ICNARC Case Mix Programme Database.* https://www.icnarc.org/Our-Audit/Audits/Cmp/Reports

7 Bronfenbrenner, U. (1979). *The ecology of human development: Experiments by nature and design*. Harvard University Press.

8 Camargo, A., Hossain, E., Aliko, S., Akinola-Odusola, D., Artus, J., & Kelman, I. (2021). Using an ecological and biological framing for an anti-racist Covid-19 approach. *MedRxi.* https://doi.org/10.1101/2021.01.24.21250397

9 Phiri, P., Delanerolle, G., Al-Sudani, A., & Rathod, S. (2021). Covid-19 and Black, Asian, and minority ethnic communities: A complex relationship without just cause. *Journal of Medical Internet Research Public Health and Surveillance*, *7*(2), e22581. https://doi.org/10.2196/22581

10 Ministry of Housing, Communities and Local Government (2020) *People living in deprived neighbourhoods.* UK Government. https://www.ethnicity-facts-

For peer review only

figures.service.gov.uk/uk-population-by-ethnicity/demographics/people-living-in-deprived-neighbourhoods/latest#main-facts-and-figures

11 Carrión, D., Colicino, E., Pedretti, N. F., Arfer, K. B., Rush, J., DeFelice, N., & Just, A. C. (2020). Assessing capacity to social distance and neighborhood-level health disparities during the Covid-19 pandemic. *MedRxiv.* https://doi.org/10.1101/2020.06.02.20120790

12 NHS Digital (2020). *NHS workforce ethnicity facts and figures.* UK Government. https://www.ethnicity-facts-figures.service.gov.uk/workforce-and-business/workforce-diversity/nhs-workforce/latest#full-page-history

13 Catney, G., & Sabater, A. (2015). *Ethnic minority disadvantage in the labour market: Participation, skills and geographical inequalities.* Joseph Rowntree Foundation, pp. 1-96. https://www.jrf.org.uk/file/46593/download?token=-98tkg_Q&filetype=full-report

14 Van Bavel, J. J., Baicker, K., Boggio, P. S., Capraro, V., Cichocka, A., Cikara, M., Crocket, M.J., Crum A.J., Douglas, K.M., Druckman, J.N., Drury, J., Dube, O., Ellemers, N., Finkel, E.J., Fowler, J.H., Gelfand, M., Han, S., Alexander Haslam, S., Jetten, J., Kitayama, S., Mobbs, D., Napper, L.E., Packer, D.J., Pennycook, G.,

For peer review only

Peters, E., Petty, R.E., Rand, D.G., Reicher, S.D., Schnall, S., Shariff, A., Skitka, L.J., Smith, S.S., Sunstein, C.R., Tabri, N., Tucker, J.A., van der Linden, S., van Lange, P., Weeden, K.A., Wohl, M.J.A., Zaki, J., Zion, S.R., & Willer, R. (2020). Using social and behavioural science to support Covid-19 pandemic response. *Nature Human Behaviour*, *4*(5), 460-471. https://doi.org/10.1038/s41562-020-0884-z

15 Public Health England. (2020) *Beyond the data: Understanding the impact of Covid-19 on BAME communities.* Department of Health and Social Care. https://assets.publishing.service.gov.uk/government/uploads/system/uploads/attachment_data/file/892376/COVID_stakeholder_engagement_synthesis_beyond_the_data.pdf

16 Mind (2020) The mental health emergency: How has the coronavirus pandemic impacted our mental health? Mind. Available at: mind.org.uk

17 Proto, E., & Quintana-Domeque, C. (2021). COVID-19 and mental health deterioration by ethnicity and gender in the UK. *PloS one*, *16*(1), e0244419. https://doi.org/10.1371/journal.pone.0244419

For peer review only - http://bmjopen.bmj.com/site/about/guidelines.xhtml

18 Geyser, L. (2021). The impact of Covid-19 on food security. A qualitative analysis of the rise in London's existing food insecurity due to the financial impact of Covid-19. *World Nutrition*, *12*(1), 40-50. https://doi.org/10.26596/wn.202112140-50

19 Alesina, A., & La Ferrara, E. (2002). Who trusts others?. *Journal of Public Economics*, *85*(2), 207-234. https://doi.org/10.1016/S0047-2727(01)00084-6

20 Demaris, A., & Yang, R. (1994). Race, alienation, and interpersonal mistrust. *Sociological Spectrum*, *14*(4), 327-349. https://doi.org/10.1080/02732173.1994.9982075

21 Farquharson, W. H., & Thornton, C. J. (2020). Debate: Exposing the most serious infirmity–racism's impact on health in the era of COVID-19. *Child and Adolescent Mental Health*, *25*(3), 182-183. https://doi.org/10.1111/camh.12407

22 Egan, S. M., Pope, J., Moloney, M., Hoyne, C., & Beatty, C. (2021). Missing early education and care during the pandemic: The socio-emotional impact of the Covid-19 crisis on young children. *Early Childhood Education Journal*, 1-10. https://doi.org/10.1007/s10643-021-01193-2

23 Dewa, L. H., Crandell, C., Choong, E., Jaques, J., Bottle, A., Kilkenny, C., Lawrence-Jones, A., Di Simplicio, M., Nicholls, D., & Aylin, P. (2021). CCopeY: A mixed-

methods coproduced study on the mental health status and coping strategies of young people during Covid-19 UK lockdown. *Journal of Adolescent Health*, *68*(4), 666-675. https://doi.org/10.1016/j.jadohealth.2021.01.009

24 Stinson, E. A., Sullivan, R. M., Peteet, B. J., Tapert, S. F., Baker, F. C., Breslin, F. J., Dick, A.S., Gonzalez, M.R., Guillaume, M., Marshall, A.T., McCabe, C.J., Pelham III, W.E., Van Rinsveld, A., Sheth, C.S., Sowell, E.R., Wade, N.E., Wallace, A.L. & Lisdahl, K. M. (2021). Longitudinal impact of childhood adversity on early adolescent mental health during the Covid-19 pandemic in the ABCD Study Cohort: Does race or ethnicity moderate findings?. *Biological Psychiatry Global Open Science*, *1*(4), 324-335. https://doi.org/10.1016/j.bpsgos.2021.08.007

25 McKinlay, A. R., May, T., Dawes, J., Fancourt, D., & Burton, A. (2021). You're just there, alone in your room with your thoughts: A qualitative study about the impact of lockdown among young people during the Covid-19 pandemic. *MedRxiv.* https://doi.org/10.1101/2021.04.11.21254776

26 Penner, F., Ortiz, J. H., & Sharp, C. (2021). Change in youth mental health during the Covid-19 pandemic in a majority Hispanic/Latinx US sample. *Journal of the American Academy of Child & Adolescent Psychiatry*, 60(4), 513-523. https://doi.org/10.1016/j.jaac.2020.12.027

For peer review only

27 Knowles, G., Gayer-Anderson, C., Turner, A., Dorn, L., Lam, J., Davis, S., Blakey, R., Lowis, K., Schools Working Group, Young Persons Advisory group, Pinfold, V., Creary, N., Dyer, J., Hatch, S.L., Ploubidis, G., Bhui, K., Harding, S., & Morgan, C. (2022). Covid-19, social restrictions, and mental distress among young people: A UK longitudinal, population-based study. *Journal of Child Psychology and Psychiatry.* https://doi.org/10.1111/jcpp.13586

28 Pascual-Sanchez, A., Nicholls, D., Patalay, P., Crosby, L., McColoud, T., Hudson, L., & Viner, R. (2020). You-COPE: Mental health consequences experienced by young people aged 16-24. *University College London. https://www.ucl.ac.uk/child-health/sites/child_health/files/youcope_briefing_mental_health_impact_final_version.pdf*

29 Mahmood, F., Acharya, D., Kumar, K., & Paudyal, V. (2021). Impact of Covid-19 pandemic on Black, Asian and Minority Ethnic (BAME) communities: A qualitative study on the perspectives of BAME community leaders. *MedRxiv.* https://doi.org/10.1101/2021.03.03.21252286

30 Burgess, R. A., Kanu, N., Matthews, T., Mukotekwa, O., Smith-Gul, A., Yusuf, I., ... & Gul, M. (2022). Exploring experiences and impact of the COVID-19 pandemic on

young racially minoritised people in the United Kingdom: A qualitative study. *Plos one*, *17*(5), e0266504 https://doi.org/10.1371/journal.pone.0266504

31 Gillard S., Dare C., Hardy J., Nyikavaranda P., Rowan Olive R., Shah P., Birken M., Foye U., Ocloo J., Pearce E., Stefanidou T., Pitman A., Simpson A., Johnson, S., Lloyd-Evans, B., & NIHR Mental Health Policy Research Unit Covid coproduction research group. Experiences of living with mental health problems during the COVID-19 pandemic in the UK: A coproduced, participatory qualitative interview study. *Social Psychiatry and Psychiatry Epidemiology 56*, 1447–1457 (2021). https://doi.org/10.1007/s00127-021-02051-7

32 Dewa, L. H., Roberts, L. F., Choong, E., Crandell, C., Demkowicz, O., Ashworth, E., Branquinho, C., & Scott, S. (2022). The impact of COVID-19 on young people's mental health and wellbeing from a European perspective: A co-produced qualitative systematic review. *Social Science Research Network.* 4266185

33 London Assembly Health Committee (2015). *Healthy minds, healthy Londoners improving access to mental health services for London's young and Black, Asian and minority ethnic population.* Available from: https://www.london.gov.uk/sites/default/files/gla_migrate_files_destination/HealthyMindsHealthyLondoners_0.pdf.

For peer review only

34 Sentell, T., Shumway, M., & Snowden, L. (2007). Access to mental health treatment by English language proficiency and race/ethnicity. *Journal of General Internal Medicine*, *22*(2), 289-293. https://doi.org/10.1007/s11606-007-0345-7

35 Khan, S., Asif, A., & Jaffery, A. E. (2021). Language in a time of Covid-19: literacy bias ethnic minorities face during Covid-19 from online information in the UK. *Journal of Racial and Ethnic Health Disparities*, *8*(5), 1242-1248. https://doi.org/10.1007/s40615-020-00883-8

36 Sainsbury Centre for Mental Health (2002). Breaking the circles of fear: a review of the relationship between mental health services and African and Caribbean communities. *Sainsbury Centre for Mental Health.* https://www.centreformentalhealth.org.uk/sites/default/files/breaking_the_circles_of_fear.pdf

37 Pro Bono Economics (2020), November 15-20 Covid Charity Tracker Survey results. *PBE Covid Charity Tracker.* Available at: https://www.probonoeconomics.com/november-15-20-covid-charity-tracker-survey-results

38 Youth Employment UK (2019). Youth Voice Census Report 2019. *Youth Employment UK*. Available at: https://www.youthemployment.org.uk/dev/wp-content/uploads/2019/05/2019-Youth-Employment-UK-Report_FINAL-1.pdf

39 Wong, K. K.-Y. (2021). COVID19: Co-creating psychological wellbeing and life-skill workshops with BAME youths in West London (COPE-WELL). https://doi.org/10.17605/OSF.IO/JCAK7

[dataset] 40 Lenoir, R. & Wong, K.K. (resubmit). The impact of the Covid-19 pandemic on young people from black and mixed-ethnic groups' mental health in West London: A qualitative study. OSF Preprint. April 11, 2023. https://osf.io/jcak7/

41 Smith, J. A. (1996). Beyond the divide between cognition and discourse: Using interpretative phenomenological analysis in health psychology. *Psychology and Health*, *11*(2), 261-271. https://doi-org.libproxy.ucl.ac.uk/10.1080/08870449608400256

42 Smith, J. A., & Osborn, M. (2015). Interpretative phenomenological analysis as a useful methodology for research on the lived experience of pain. *British Journal of Pain*, *9*(1), 41-42. https://dx.doi.org/10.1177%2F2049463714541642

For peer review only

43 Smith, J. A., & Shinebourne, P. (2012). Interpretative phenomenological analysis. In H. Cooper, P. M. Camic, D. L. Long, A. T. Panter, D. Rindskopf, & K. J. Sher (Eds.), *APA handbook of research methods in psychology, Vol. 2. Research designs: Quantitative, qualitative, neuropsychological, and biological* (pp. 73–82). American Psychological Association. https://doi.org/10.1037/13620-005

44 Alase, A. (2017). The interpretative phenomenological analysis (IPA): A guide to a good qualitative research approach. *International Journal of Education and Literacy Studies*, *5*(2), 9-19. http://dx.doi.org/10.7575/aiac.ijels.v.5n.2p.9

45 Goldspink, S., & Engward, H. (2019). Booming clangs and whispering ghosts: Attending to the reflexive echoes in IPA research. *Qualitative Research in Psychology*, *16*(2), 291-304. https://doi.org/10.1080/14780887.2018.1543111

46 Wade, M., Prime, H., & Browne, D. T. (2020). Why we need longitudinal mental health research with children and youth during (and after) the Covid-19 pandemic. *Psychiatry Research*, *290*, 113143. https://dx.doi.org/10.1016%2Fj.psychres.2020.113143

47 Scott, S. R., Rivera, K. M., Rushing, E., Manczak, E. M., Rozek, C. S., & Doom, J. R. (2021). "I hate this": A qualitative analysis of adolescents' self-reported challenges

For peer review only

during the COVID-19 pandemic. *Journal of Adolescent Health*, *68*(2), 262-269.: https://doi.org/10.1016/j.jadohealth.2020.11.010

48 Colombo, D., Suso-Ribera, C., Fernández-Álvarez, J., Cipresso, P., Garcia-Palacios, A., Riva, G., & Botella, C. (2020). Affect recall bias: Being resilient by distorting reality. *Cognitive Therapy and Research*, *44*(5), 906-918. https://doi.org/10.1007/s10608-020-10122-3

49 Raphael, K. (1987). Recall bias: A proposal for assessment and control. *International Journal of Epidemiology*, *16*(2), 167-170. https://doi.org/10.1093/ije/16.2.167

50 Wright, C., Maylor, U., & Becker, S. (2019). Young black males: Resilience and the use of capital to transform school “failure”. In Alternative Educational Programmes, Schools and Social Justice (pp. 21-34). Routledge https://doi.org/10.1080/17508487.2016.1117005

51 Richter, D., Riedel-Heller, S., & Zuercher, S. (2021). Mental health problems in the general population during and after the first lockdown phase due to the SARS-Cov-2 pandemic: Rapid review of multi-wave studies. *Epidemiology and Psychiatric Sciences*, 1-17. https://doi.org/10.1017/S2045796021000160

52 Skripkauskaite, S., Shum, A., Pearcey, S., McCall, A., Waite, P., & Creswell, C. (2021). Changes in children's mental health symptoms from March 2020 to June 2021 (Report 11). *Co-SPACE Study*. http://cospaceoxford.org/findings/changes-in-childrens-mental-health-symptoms-from-march-2020-to-june- 2021/

53 Department for Work and Pensions (2021) *Family resources survey (FRS) for the financial year 2019 to 2020, providing information on income and circumstances of UK households.* UK Government. https://www.ethnicity-facts-figures.service.gov.uk/work-pay-and-benefits/pay-and-income/household-income/latest#download-the-data

54 Memon, A., Taylor, K., Mohebati, L. M., Sundin, J., Cooper, M., Scanlon, T., & de Visser, R. (2016). Perceived barriers to accessing mental health services among black and minority ethnic (BME) communities: A qualitative study in Southeast England. *British Medical Journal Open*, *6*(11), e012337. http://dx.doi.org/10.1136/bmjopen-2016-012337

55 Peters, S. (2010). Qualitative research methods in mental health. *Evidence-based Mental Health*, *13*(2), 35-40. http://dx.doi.org/10.1136/ebmh.13.2.35

56 Dayan, Y., Paine, C. S., & Johnson, A. J. (2007). Responding to sensitive questions in

surveys: A comparison of results from online panels, face to face, and self-completion interviews. *World Association for Public Opinion Research.*

57 Dempsey, L., Dowling, M., Larkin, P., & Murphy, K. (2016). Sensitive interviewing in qualitative research. *Research in Nursing & Health*, *39*(6), 480-490. https://doi.org/10.1002/nur.21743

58 Shapka, J. D., Domene, J. F., Khan, S., & Yang, L. M. (2016). Online versus in-person interviews with adolescents: An exploration of data equivalence. *Computers in Human Behavior*, *58*, 361-367. https://doi.org/10.1016/j.chb.2016.01.016

59 Solmi, M., Estradé, A., Thompson, T., Agorastos, A., Radua, J., Cortese, S., … & Ifteni, P. (2022). Physical and mental health impact of COVID-19 on children, adolescents, and their families: The Collaborative Outcomes study on Health and Functioning during Infection Times-Children and Adolescents (COH-FIT-C&A). *Journal of Affective Disorders,* 299, 367-376. https://doi.org/10.1016/j.jad.2021.09.090

60 Islam, N., Khunti, K., Dambha-Miller, H., Kawachi, I., & Marmot, M. (2020). COVID-19 mortality: a complex interplay of sex, gender and ethnicity*. European Journal of Public Health*, *30*(5), 847-848. https://doi.org/10.1093/eurpub/ckaa150

61 Rajgopal, A., Li, C. R., Shah, S., & Sundar Budhathoki, S. (2021). The use of telehealth to overcome barriers to mental health services faced by young people from Afro-Caribbean backgrounds in England during the Covid-19 pandemic. *Journal of Global Health*, 11, 03040. https://doi.org/10.7189/jogh.11.03040

62 Hammelsbeck, R., Mitchell, M., Thornton, I., Caroline, W., Taylor, B., & Bristow, T. (2021). Evaluation of the Youth Covid-19 Support Fund. *National Centre for Social Research. https://dera.ioe.ac.uk//38969/1/Youth_Covid-19_Support_Fund_-_Evaluation_Report_final__WEB_.pdf*

63 Murray, K. (2020). Impact of Covid-19 on the BAME community and voluntary sector. *The Ubele Initiative.* https://www.ubele.org/assets/documents/REPORT-Impact-of-Covid-19-on-the-BAME-Community-and-voluntary-sector-30-April-2020.pdf

64 UN General Assembly (1989), *Convention on the Rights of the Child*. United Nations, Treaty Series, vol. 1577. https://www.unicef.org/child-rights-convention/convention-text

65 Hu, Y. (2020). Intersecting ethnic and native–migrant inequalities in the economic impact of the Covid-19 pandemic in the UK. *Research in Social Stratification and Mobility*, *68*, 100528. https://doi.org/10.1016/j.rssm.2020.100528

66 Hamilton, S., Pinfold, V., Cotney, J., Couperthwaite, L., Matthews, J., Barret, K., Warren, S., Corker, E., Rose, D., Thornicroft, G., & Henderson, C. (2016). Qualitative analysis of mental health service users' reported experiences of discrimination. *Acta Psychiatrica Scandinavica*, 134, 14-22. https://doi.org/10.1111/acps.12611

67 Proto, E., & Quintana-Domeque, C. (2020). Covid-19 and mental health deterioration among BAME groups in the UK. *Social Science Research Network.* https://dx.doi.org/10.2139/ssrn.3662057

For peer review only

Appendix A

Initial Interview Schedule

Introduce the research team and build rapport. Present the consent form and information sheet, go over ethics and take questions. Finally, explain rationale of the study, why we are here, aim to build trust.

Part 1- *Broad questions about impact of Covid-19*

1. How has your experience of the pandemic been?
   1.1. If mention education, how did it feel not going to school anymore?
   1.2. If mention home environment, how did it feel spending more time at home?
2. How were your relationships with your friends and family?
3. What were some of your favourite or least favourite things about covid?
4. How did covid impact your physical health?
   4.1. If mention close experience with covid, how did that made them feel?
5. How did Covid-19 impact your mental health?
   5.1. If mention of worry or such feelings, how did that feel like? what made it worse or better for you? If you opened up, who did you feel comfortable talking to?
6. Has your perception of the media, governments and institutions during the pandemic affected your trust in them?

Part 2- *Changes throughout the pandemic*

7. What about now, how have things changed since then?
   7.1. If no direction, what are the biggest challenges now? What were the biggest changes in your life?
8. How have your physical and mental health changed since lockdown?
   8.1. If need guidance, are they better or worse now?

For peer review only

9. How is your perception of the media, governments and institutions now, after the peak of the pandemic has passed and how has your trust in them changed?

Part 3- *Support needed to deal with these issues*

10. Did you receive any mental support during the pandemic?

    10.1. If so, how did you access it? What form did the support take and how did it make you feel?

    10.2. If not, did you feel like you would have needed some? what kind?

11. What aspects of your life do you feel you would need more support in?

12. What kind of support would you need now and how would you like to access it?

Debrief, thank you and share next steps of the research project.

For peer review only

Appendix B

Final Interview Schedule

Introductions - Researchers will introduce themselves, along with JED CEO and social worker and any student researchers present. Thank young people for taking part and joining the focus group. Build rapport. Walk through participant information sheet with young people, read verbatim each item on the consent form to ensure participants understand. Take questions.

Lay groundwork for why we're conducting this project, project aims, what we hope to learn and project outputs.

Express ethical considerations (right to withdraw at any time, don't have to share information they don't want to, don't have to answer all questions, confidentiality, trust, safe space to share, no right or wrong answers). Again, emphasize that researchers are here to learn from young people, in order to help other young people in the future as we navigate out of the COVID pandemic. Remind young people the session will be split into 2 parts and last between 30-40 minutes.

QUESTIONS

Part 1 – barriers to health access, impact of covid on health and changes

1. Thinking over this past year, how has it been for you?
    a. Prod: What are some things you are grateful for, or challenges that you faced?
2. Did you learn anything about yourself or others around you? How were you close relationships?
3. Thinking about this past year, has the pandemic affected the way you trust in others? Institutions, governments, community, people?
4. What were some highlights?
5. How has covid impacted your health – mentally and physically?

For peer review only

6. Thinking about pre-pandemic and now, what were some things that has changed for the better? Or for the worse?
7. Thinking about pre-pandemic and now, how has your health changed for the better? Or for the worse?
8. Thinking about some of your challenges, what are some examples of your struggles/challenges?
9. What did you enjoy or least enjoy during the pandemic?

Part 2 – Support that young people would like or need

1. What support did you receive during the pandemic, if any?
2. How was the support that you received, if any?
   a. How did you come across this support?
   b. What were the barriers to the support? If any?
   c. If no barriers, what made it accessible?
3. If you could get more support during the pandemic – and now – what might you want/need?
4. Thinking specifically about your health (physical/mental), what do you wish you had known? What support would you want?
5. What do you think are the key areas that you need most support on? Where might you go to get support?

Debrief and thank young people for their time. Inform next steps in project on co-creating workshops together

For peer review only

Appendix C

Patterns clusters

… : aim 1: mental health

Patterns of negative mental health
ISOLATION FROM OTHERS: the most
Isolation IIIIIIIIIIIII **Loneliness** IIIIIIII **Broken community** II **Lack of social interactions** IIIIIII **Loosing friendships** IIII
ILLNESS-RELATED DISTRESS: the 2nd most
COVID anxiety IIIIII **Death** IIII **Media overload** II **Overwhelming focus on physical** II
FAMILY ISSUES: sometimes
Home environment III
EDUCATION: sometimes
Online learning IIII
BAD MENTAL HEALTH: often
Overall hard MENTAL HEALTH IIIII
TRUST: often
Trust issues (friends/institutions) IIII
SADNESS: frequent
Sadness II **Depression** IIIII
STRESS: often
Stress and anxiety III **Overthinking** I **Fear** I
CLINICAL: frequent
Suicide/suicidal ideations III **Eating disorders/body image** IIIII **ADHD** I
LACK OF CONTROL: sometimes
Lack of usual coping/escape mechanisms II **Helpless** I
SELF-ESTEEM: frequent
Self-esteem IIIIII
APATHY/NUMB: frequent
Laziness III **Loss of structure** I **Lack of motivation** IIIII **Nonchalance** I **Boring** I **Tired** III
ANGER: often
Anger II **Frustration** IIII **Triggering** I

Young people III **Collective denial** I

Patterns of positive mental health
CLOSER INTERACTIONS: sometimes
Closer bonds (family, friends) III **Meeting people online** I
GRATEFULNESS: often
Grateful IIII
NEW HABITS: the most
New mindful practices IIIIIIII
SOLITUTDE: often
Alone time II **Resilience** II **Self-actualisation** IIII
EDUCATION: rarely
Time to study II

For peer review only

…: aim 2: changes with time

NOSTALGIA: rarely
Nostalgia I
SOCIAL INTERACTION HELPS: the most
After lockdown peak with seeing people again IIIIIIIIIII
SELF-RELIANCE SO IMPROVEMENT WITH TIME: the 2nd most
Mental health improving because of self-reliance after bad period/lockdown IIIIII
GRATEFUL: rarely
Grateful for themselves, for being safe I

…: aim 3: support

During COVID
LACK OF SUPPORT: the most
Lack of IIIII
SCHOOL: the 2nd most
School lacks/ don't want IIIII **School as a point of access** II
CLOSE PEOPLE: often
Friends II **Self-help** I **Family** II **Lack of family** I
LACK OF LEGITIMITY: often
illness not seen as legitimate (esp awareness ass BAME YP+gender) III **Reluctant to reach out** II
FINANCIAL/TECHNICAL: sometimes
Financial I **Technical** I

Now Want
ACTIVITIES: frequent
Activities IIIII
MOTIVATION: sometimes
Healthy lifestyle I **Motivational support** I
MENTAL HEALTH: most often
Mental health IIIII **Intense relief when getting support** I
EDUCATION: often
Educational I **School support** II
YOUTH CLUBS: often
Youth club III
ARTS: frequent
Arts IIII
TO BE HEARD: 2nd most
Listened to IIIII
TO LEARN: sometimes
Learning about new issues II
SPORTS: often
Social sports IIII

For peer review only

WORK EXPERIENCE: rarely

Work experience I

Reaching out I

LACK OF: sometimes

Lack of adequate support (safe, accessible) II

SCHOOL AS A BARRIER: sometimes

School as a barrier II

For peer review only

Appendix D

Initial Thematic Tables

Superordinate theme 1: Impact on mental health

A. Loneliness

Lack of social interactions

P103: 'lockdown obviously had a like really big impact on my mental health like it made me feel way more alone that like I actually was cause I couldn't go outside obviously.'

P103: 'I enjoyed the alone time the most but then it's also what like triggered me the most. I just kept I felt so trapped (…) And isolated but I also felt isolated and also felt like alone and unable to be myself in my own space.'

P104: 'But in that moment you're just like…I just want to be alone and it doesn't help it's like you're scurrying into a little hole (…) So I feel like also not having…people to talk to cause you can have like a million people around you and still feel alone. So I felt like in that moment I felt so alone and...tired and mentally exhausted and then school, and then.. not seeing friends and family members and the death it all just- (…) I feel like a lot of people different ages went through that same experience with like it just starts affecting you.'

P106: 'I felt actually quite sad (…) and angry because I'm at home. And like I just feel too much like energy in the house and you're too hot and you can't do this you can't do that and like you can't go out and that's like what frustrates me'

Self-esteem

P103: 'I had my brothers and my mum obviously but like they don't understand obviously, and um like I just really need someone to tell me like you're good enough (…) and like that's all like that's all anyone needs to hear, they just want validation (…) I just want to feel appreciated and like-like I just want to feel stuff like that.'

P103: 'There was a lot of challenges with like my mental health and like my self-esteem and like I- I still don't but like, I never believed in myself like at all, like at all/ (…) before I had no faith in myself like I used to believe that I couldn't do anything, I just like feel like I was never going to be good enough'

B. Mental disorders

Anxiety, Depression and Disordered eating

P104: 'but as well as mentally it really deteriorated like all youth, people my age like I realised that everybody was finding out they had depression, anxiety, it was really like draining on staying put, some people had toxic family members they had to stay home it was really like..it was hard.'

P104: 'I realised a lot of things which kind of drove me into a.. I would say quite a depression in the sense of like I wouldn't.. I would just staying in my room the whole time, I wouldn't leave it, stay on my phone but then log off every social media app possible to kind of just block out everything'

P103: 'I lost a lot of weight after the pandemic not in the best way. I didn't really eat, um but like (…)I never really used to- used to give myself like the attention I deserve, um like um and do you know with COVID it made it very very bad like my mental health was very bad, I was down there it was so bad. (…) and it was so such a heavy weight on my heart, it was like it was really hard for me and COVID made it all worse like trapped in my room seeing the same four walls everyday (…) so I was losing it, I was losing it.'

P100: 'Mentally, it affected me both like oh am I going to get COVID next, like got some sort of anxiety maybe with me.'

P102: 'Umm I've been sad, depressed…It was hard but I had to do it'

Trauma

P014: 'Like it just caused a lot of like thinking and..kind of just a lot of like reevaluating life and everything especially being in lockdown in your house and people passing away drastically like every day the numbers were increasing like I felt (…) there was times you really have to stop and think like this type of like disease this thing is wiping everybody out (…) anybody could have died from it.'

P104: 'This is really happening and nobody was grasping with the fact that people are really dying, (…) like it was sad that so many people

For peer review only

P104: 'I realised that I had a lot of mental health problems within myself, and I wasn't not really happy, um not very confident, quite insecure'<br><br>*Lack of trust in others/institutions*<br>P104: 'But I feel like everybody was more out for themselves and not really trying to really help each other (…) so it may have cause some type of division.. which kind of broke- I feel like the community was kind of broken (…) Like at some point, we weren't together, collective and stuff.'<br><br>*Lack of motivation*<br>P107: 'And also um I feel like because I haven't been doing a lot I've lost the motivation to actually do things.'<br>P107: 'Um mentally, I think yeah as I said before my motivation has taken like a big decline and also obviously I feel like bad habits during COVID there's not really much that you can do.'<br>P110: 'A lot, uh yeah like laziness, um can't get out of bed as much, um yeah just a lot of that.'	couldn't handle COVID they took their lives like it was..it was absolutely devastating really, so yeah it was just so sad.'<br><br>**C. Positive impact on mental health**<br><br>*New connections*<br>P101: 'I met lots of new people and got to interact with some people during lockdown so that was good,'<br>P104: '(People could) talk to each other, sit down and really think like what is going on in the world.'<br>P105: 'but like we have gotten closer and we like text my friends during in the middle of class when I was in lockdown and we'd sometimes have video calls and stuff.'<br>P107: 'Yeah um I've got a lot closer with a lot of my friends'.<br><br>*New mindful activities*<br>P100: 'Um, I was able to try things that I wouldn't try on a day to day basis (…) like going for walks and stuff and like doing drawings.'<br>P101:'I started to find new hobbies like poetry, um and we started going to this poetry night which was like good'<br>P104: 'during summer like obviously summer it gets dark really late um I used to go for like walks around my neighbourhood and obviously I had like no phone nothing so like I would just like I just used to reflect and it was such a like a peaceful time like after I came back home I thought like..I just felt like free. I felt like there was no **** nothing.'<br><br>*Solitude*<br>P100: '(I enjoyed) having me time. Like being able to do like stuff on my own.'<br>P107: 'Um, things I enjoyed the most I'd probably say I got a lot of time to myself.'

Superordinate theme 2: Changes in mental health since the start of lockdown	
**A. Post-lockdown high**<br><br>*Freedom*<br>P103: 'But like at the end of lockdown when everything like um the restrictions started lifting I felt like I was finally getting life back.'	**B. Gradual mental health coping**<br><br>*Isolation and overthinking*<br>P102: 'I had to do it because if I didn't then how am I supposed to go in life so I literally just looked at the present and just focusing on what I need to do to get through my life.'

For peer review only

P106: 'going out in groups without having to get COVID tested themselves before they left the house.'
P107: 'Cause now that I've finally got the freedom to be out of my house (…) I was out of my house all the time.'

Social closeness
P104: 'Seeing my friends for the first time. Like I can't fake that that was like the best feeling cause it's like all these months I haven't seen you and it's like oh my god.'
P104: 'And I would see no one, it was so.. everyone was scared for their lives, like nobody was outside..it was like (sigh) so I guess when I started seeing the community, everybody kind of coming, trying to make this new normal, I guess that was really nice'
P107: 'when the lockdown when the restrictions first eased like in the summer of 2020. (…) Yeah I feel like then I got a lot closer with a lot of people.'
P109:'Back then I wasn't as social (…) Cause I found a really good series of books and I just kept reading it.(…) It's not that I couldn't be social it's just that I wasn't bothered to, but now I'm like talking a bit more, still reading though.'
P110: 'Good, (friends and family relationships) got even stronger since we could interact again.'

P103: 'Cause it was a very long time just self-reflecting and like, I liked it- it was very therapeutic but at the same time I hated it I felt like I was being cooped up and like trapped/'
P103: 'started overthinking like I started to like really, evaluate my personality and like who I am as a person (…) and like my mental health as well cause I've never really thought about mental health at all, ok I'm not ok but there's nothing I can do'
P103: 'before the pandemic I never really like cared about myself (…)I think that was mainly because there was all this stuff like all this stuff that was just built up and I never had a chance to release it cause I was always (…) so I never had to think about it.'

Better coping
P102: 'I have ADHD (…) So I know what time I'll be coming home and (my mum) will have checked on me cause I'll be doing silly stuff so, like stuff like that has changed me and normally it would ne- I would never do that so I'm happy that I've done that. (…)I'm organised better. (…) So that's like COVID, I think COVID actually helped me do all of this stuff, so yeah'
P103: 'those little things that I did like I used to go on walks or I just used to like I used to write down my thoughts a lot like it was just like random bits of what I'm feeling throughout the day and like those kind of help you feel better.'
P103: 'when I realised it's like there so many different things that I can do to help myself and I just want to find all the right um outlets to project any um anger or aggression any uh sad feelings that I had without causing violence.'

Better mental health
P102: 'Uh, in my opinion I've known I've been depressed I know I've been all down and whatever, but I think if I didn't have that stage in my life I would not be what who I am right now'
P102: 'I think I've changed for the better cause I used to be like a naughty kid. (…) So now I've like realised like I need to learn, revise,.. no friends like everything changed and I kind of like it because I've never been like this before so'
P102: 'So like, a few years ago I was crazy, I was hanging around with the wrong crowd, and now I've realised like, I've realised I've grown and I have to show my little sister that I can't be

For peer review only

	like who I am right now, so I had to change my personality just for her (…) cause if it wasn't for her then I would still be behaving bad and yeah'<br>P103: 'And um like I think it strengthened my relationships but it also strengthened the relationship that I had with myself which is like one of the most important ones, so yeah'<br>P103: 'I think I'm good now, I think if you'd asked- I think if you'd done this interview like a couple months ago (…) I was so stuck, I would have been like I need someone to just talk to.'

Superordinate theme 3: Support obtained during pandemic and needs now	
**A. Support**	**B. Needs**
Lack of support<br>P100: 'Honestly little to none, support yeah'<br>P103:'I wish I would've know that there's people that I can talk to, or like there's things that I can do to like lose weight healthier rather than just starving myself or I wish there was someone to tell you that like you're enough'<br>P104: 'people I know have developed eating disorders, people that maybe had trauma or shock from a lost one like .. it all stems mentally and I feel like if they would've had mental help, when you neglect your mental health you neglect your physical health, it all connects.'<br>P110: 'I wouldn't say there was support, it was just really independent.'<br><br>*Neglect*<br>P102: 'I would actually listen to people because clearly the government doesn't listen to no one he just he does everything what he thinks and I don't I don't trust that, and I don't believe in that so I will, if I was the government, of course I would change everything, I would listen to everybody, even the poor, change a lot of things. (…) I didn't care about that (before COVID) but now I do.'<br>P102: 'I know I have issues (…) It's just like, I need more support with that um. (…) No one is listening. So I have to shout it out loud so everyone can listen, so yeah.'<br>P104: 'like in this generation and age there are so many misconceptions about teenagers, especially me as a black female teenager, there's many misconceptions about…being…you know who you are or what you look like'<br><br>*Structural*	*Psychotherapy*<br>P103: 'if that person is trained to deal with like mental health issues it's way easier to like open up to them and like when I got a counsellor and a mentor like I finally felt like I could talk to someone else about it.'<br>P104: 'I feel like sometimes psychologically, just like someone to understand and hear me (…) it's hard to kind of like, to speak to somebody without your mind being like oh maybe this person thinks I'm crazy, it would be nice to be finally heard by someone and to talk about your feelings and interests without someone, or your feelings that you felt before, without someone judging you. Because sometimes you just, you just need an ear. You just need someone to listen. I feel like that would really help actually. And it would've helped I think back then if I'd had that I think it would have been way easier to talk about.'<br>P104: 'and (the therapist) just said what you described does sound like it, and I was like, in that moment like a tear fell on my face because I was like really like it's been confirmed, like maybe he didn't say yeah you have depression but he basically said you're on the way to it,'<br>P106: ' (I would need) Like people speaking to me.'<br><br>*Relational*<br>P103: 'I still have support but it's like from my friends.'<br>P105: 'I would have like hugs from my friends. (…)I felt like it's a way of comforting myself and comforting other people when it's like hardships and stuff. (…) It just like helps a lot. Because even the simplest thing, like love can do a lot.'

For peer review only

P103: ‘it’s like sometimes we don’t have the best relationship with teachers and like because of the past I’ve been at that school for what like 4 years. (…) there’s like background there’s a past with some of the teachers and it’s not really the best to confide in them’.<br>P100: ‘just like in school, it’s very much like,…once in a blue moon ask us if we’re alright and that’s it.<br>P106: ‘they don't help they only sit in my class and do nothing when I ask for help then sometimes they listen.’	P104: ‘Um to be honest I actually didn't (reach out) (…) I feel like I made that little depressive state my normal. (…) So I thought- I also didn't reach out I didn't think there was anything wrong with it (…) and it’s kind of like, asking for help I’m not really a person that does that. (…)I’m not really a person that goes out for support so that denial was kind of like a comfort place like no you don't need anybody you’re fine like you don't need to talk to anybody you're ok, like people who talk to people you don't you don't need that you're not on that level you're ok so I kind of talked myself into not getting help, not speaking to anybody, which caused me to feel really alone.’<br><br>*Need for daily life*<br>P107: ‘I would say maybe more opportunities available. So stuff like sports. Even something like work experience. Sort of that yeah I feel like that would help a lot cause it would help like ease back into things.’<br>P107: ‘I've got very used to the connections I have now cause that's who I rely on for a while, so making new friendships, relationships, has become a lot harder.’<br>P100: ‘About how to maintain a healthy lifestyle even though…circumstances can change like this (fingers snapping).’<br>P105: ‘But I like to talking about things like that and painting and drawing..it's really nice.It makes me happy. (…) I like doing photography without being restricted.’<br>P106: ‘I go to (a youthclub) and do some art and yeah.

For peer review only

Appendix E
Final Thematic Tables

Superordinate theme 1: Impact of the Covid-19 pandemic on mental health

A. Individual

Self-esteem

P103: 'I had my brothers and my mum obviously but like they don't understand obviously, and um like I just really need someone to tell me like you're good enough (…) and like that's all like that's all anyone needs to hear, they just want validation (…) I just want to feel appreciated and like-like I just want to feel stuff like that.'

P103: 'There was a lot of challenges with like my mental health and like my self-esteem and like I- I still don't but like, I never believed in myself like at all, like at all/ (…) I just like feel like I was never going to be good enough'

P104: 'I realised that I had a lot of mental health problems within myself, and I wasn't not really happy, um not very confident, quite insecure'

Lack of motivation

P107: 'I feel like because I haven't been doing a lot I've lost the motivation to actually do things.'

P107: 'Um mentally, I think yeah as I said before my motivation has taken like a big decline and also obviously I feel like bad habits during COVID there's not really much that you can do.'

P110: 'A lot, uh yeah like laziness, um can't get out of bed as much.'

B. Symptoms of mental illness

Anxiety, Depression and Disordered Eating

P100: 'Mentally, it affected me both like oh am I going to get COVID next, like got some sort of anxiety maybe with me.'

P102: 'Umm I've been sad, depressed…It was hard but I had to do it'

P103: 'I lost a lot of weight after the pandemic not in the best way. I didn't really eat (…)I never really used to give myself like the attention I deserve and you know with COVID it made it very very bad like my mental health was very bad, I was down there it was so bad. (…) **and it was so such a heavy weight on my heart, it was like it was really hard for me and**

C. Relational

Isolation and individualisation

P103: '(lockdown had a) big impact on my mental health like it made me feel way more alone that like I actually was cause I couldn't go outside obviously. (…) I also felt isolated and also felt like alone and unable to be myself in my own space.'

P104: 'cause you can have like a million people around you and still feel alone. So I felt like in that moment I felt so alone and...tired and mentally exhausted (…) But I feel like everybody was more out for themselves and not really trying to really help each other (…) so it may have cause some type of division.. **I feel like the community was kind of broken'**

P106: 'I felt actually quite sad (…) and angry because I'm at home. And like I just feel too much like energy in the house and you're too hot and you can't do this you can't do that and like you can't go out and that's like what frustrates me'

Positive solitude

P100: '(I enjoyed) having me time. Like being able to do like stuff on my own.'

P107: 'Um, things I enjoyed the most I'd probably say I got a lot of time to myself.'

P104: 'during summer like obviously summer it gets dark really late um **I used to go for like walks around my neighbourhood** and obviously I had like no phone nothing so like I would just **like I just used to reflect and it was such a like a peaceful time like after I came back home I thought like..I just felt like free. I felt like there was no **** nothing.'**

New connections

P101: 'I met lots of new people and got to interact with some people during lockdown'

P104: '(People could) talk to each other, sit down and really think like what is going on in the world.'

P104: 'I definitely lost friendships and gained friendships.Like I found true friends and I also lost people that I thought were good friends to me, but it just showed that they weren't good friends at all.'

For peer review only

**COVID made it all worse (…) so I was losing it, I was losing it.'**<br>**P104**: **'I realised a lot of things which kind of drove me into a.. I would say quite a depression in the sense of like I would just staying in my room the whole time, I wouldn't leave it,** stay on my phone but then log off every social media app possible to kind of just block out everything'<br><br>*Trauma*<br>P014: 'people passing away drastically like every day the numbers were increasing like I felt (…) there was times you really have to stop and think like this type of like disease this thing is wiping everybody out (…) anybody could have died from it.'<br>**P104: '**This is really happening and **nobody was grasping with the fact that people are really dying, (…) like it was sad that so many people couldn't handle COVID they took their lives like it was..it was absolutely devastating.'**	P105: 'we have gotten closer and we like text my friends during in the middle of class when I was in lockdown and we'd sometimes have video calls.'

Superordinate theme 2: Changes in mental health since the end of lockdown	
A. **Autonomous adaptive coping skills**	**B. Lifted restrictions**
Overthinking<br>**P102: 'I had to do it because if I didn't then how am I supposed to go in life so I literally just looked at the present and just focusing on what I need to do to get through my life.'**<br>**P103: '**started **overthinking like I started to like really, evaluate my personality and like who I am as a person** (…) and like my mental health as well cause I've never really thought about mental health at all, ok I'm not ok but there's nothing I can do'<br>P103: 'before the pandemic I never really like cared about myself (…)I think that was mainly because there was all this stuff like that was just built up and I never had a chance to release it cause I was always out so I never had to think about it, and COVID made it all worse'<br><br>*Better coping*<br>P102: 'I have ADHD (…) so I've set timers to go out for 5 minutes, take my walks. I tell my mum, she has a timer on her phone and I know what time I'll be coming home and (my mum)	*Freedom*<br>**P103: 'At the end of lockdown when everything like um the restrictions started lifting I felt like I was finally getting life back.'**<br>**P106: 'going out in groups without having to get COVID tested themselves before they left the house'**[1]**.**<br>P107: 'Cause now that I've finally got the freedom to be out of my house (…) I was out of my house all the time.'<br>**P107: 'I took things for granted because obviously something as simple as being outside and going to the shops it's all changed.'**<br><br>*Social bonds*<br>**P104: 'Seeing my friends for the first time. Like I can't fake that that was like the best feeling cause it's like all these months I haven't seen you and it's like oh my god.'**<br>**P107: 'when the restrictions first eased like in the summer of 2020. (…) Yeah I feel like then I got a lot closer with a lot of people.'**

1

For peer review only - http://bmjopen.bmj.com/site/about/guidelines.xhtml

will have checked on me cause I'll be doing silly stuff so, like stuff like that has changed me and normally it would ne- I would never do that so I'm happy that I've done that. (…)I'm organised better. (…) So that's like COVID, I think COVID actually helped me do all of this stuff, so yeah'<br>**P103: 'those little things that I did like I used to go on walks or I just used to like I used to write down my thoughts a lot like it was just like random bits of what I'm feeling throughout the day and like those kind of help you feel better.'**<br>**P103: 'when I realised it's like there so many different things that I can do to help myself and I just want to find all the right um outlets to project any um anger or aggression any uh sad feelings that I had without causing violence.'**<br><br>*Personal growth*<br>**P102**: 'I've been depressed I know I've been all down and whatever, **but I think if I didn't have that stage in my life I would not be what who I am right now** (…) I think I've changed for the better cause I used to be like a naughty kid. (…) So now I've like realised like I need to learn, revise,.. like everything changed and I kind of like it because I've never been like this before.'<br>P102: 'So like, a few years ago I was crazy, I was hanging around with the wrong crowd, and now I've realised like, I've realised I've grown and I have to show my little sister that I can't be like who I am right now, so I had to change my personality just for her (…) cause if it wasn't for her then I would still be behaving bad'<br>**P103**: 'And um like I think it strengthened my relationships but **it also strengthened the relationship that I had with myself which is like one of the most important one'**<br>P103: 'I think I'm good now, I think if you'd asked- I think if you'd done this interview like a couple months ago (…) I was so stuck, I would have been like I need someone to just talk to.'<br>**P109:'Back then I wasn't as social (…) Cause I found a really good series of books and I just kept reading it.(…) It's not that I couldn't be social it's just that I wasn't bothered to, but now I'm like talking a bit more, still reading though.'**	P110: 'Good, (friends and family relationships) got even stronger since we could interact again.'

For peer review only

Superordinate theme 3: Limited support obtained during the Covid-19 pandemic and current needs

A. Support

Lack of support
P100: 'Honestly little to none, support yeah'
P103:'I wish I would've know that there's people that I can talk to, or like there's things that I can do to like lose weight healthier rather than just starving myself or I wish there was someone to tell you that like you're enough'
P104: 'people I know have developed eating disorders, people that maybe had trauma or shock from a lost one like .. it all stems mentally and I feel like if they would've had mental help, when you neglect your mental health you neglect your physical health, it all connects.'
P110: 'I wouldn't say there was support, it was just really independent.'

Neglect
P102: 'I would actually listen to people because **clearly the government doesn't listen to no one he just he does everything what he thinks and I don't I don't trust that**, and I don't believe in that so I will, if I was the government, of course I would change everything, I would listen to everybody, even the poor, change a lot of things. (…) I didn't care about that (before COVID) but now I do.'
P102: 'I know I have issues (…) It's just like**, I need more support with that. (…) No one is listening. So I have to shout it out loud so everyone can listen,** so yeah.'
P104: 'like **in this generation and age there are so many misconceptions about teenagers, especially me as a black female teenager, there's many misconceptions about…being…you know who you are or what you look like'**

Structural
P103: 'it's like sometimes we don't have the best relationship with teachers and like because of the past I've been at that school for what like 4 years. (…) there's like background there's a past with some of the teachers and it's not really the best to confide in them'.
P100: 'in school, it's very much like,…once in a blue moon ask us if we're alright and that's it.'

B. Needs

Psychological
P103: 'if that person is trained to deal with like mental health issues it's way easier to like open up to them and like when I got a counsellor and a mentor like I finally felt like I could talk to someone else about it.'
P104: 'I feel like sometimes psychologically, just like someone to understand and hear me (…) it's hard to kind of like, to speak to somebody without your mind being like oh maybe this person thinks I'm crazy, it would be nice to be finally heard by someone and to talk about your feelings and interests without someone, or your feelings that you felt before, without someone judging you. Because **sometimes you just, you just need an ear. You just need someone to listen. I feel like that would really help actually. And it would've helped I think back then if I'd had that I think it would have been way easier to talk about.'**
P104: 'and (the therapist) just said what you described does sound like (depression), and I was like, in that moment like a tear fell on my face because I was like really like it's been confirmed, like maybe he didn't say yeah you have depression but he basically said you're on the way to it,'
P106: ' (I would need) Like people speaking to me.'

Relational
P103: 'I still have support but it's like from my friends.'
P105: 'I would have like **hugs from my friends. I feel like it's a way of comforting myself and comforting other people when it's like hardships and stuff, it just like helps a lot because even the simplest thing, like love can do a lot.'**
P104: 'Um to be honest I actually didn't (reach out) (…) I feel like I made that little depressive state my normal. (…) So I thought- I also didn't reach out I didn't think there was anything wrong with it (…) I'm not really a person that goes out for support so that denial was kind of like a comfort place like no you don't need anybody you're fine like you don't need to talk to anybody you're ok, like people who talk to people you don't you don't need that you're not on that level

For peer review only

**P106: 'they don't help they only sit in my class and do nothing when I ask for help then sometimes they listen.'**	you're ok so I kind of talked myself into not getting help, not speaking to anybody, which caused me to feel really alone.'<br><br>*Practical*<br>**P107: '**I would say maybe **more opportunities available. So stuff like sports. Even something like work experience. Sort of that yeah I feel like that would help a lot cause it would help like ease back into things (…) making new friendships, relationships, has become a lot harder.'**<br>**P100**: 'About **how to maintain a healthy lifestyle even though…circumstances can change like this (snaps fingers).'**<br>P105: 'But I like to talking about things like that and painting and drawing..it's really nice.It makes me happy. (…) I like doing photography without being restricted.'<br>P106: 'I go to (a youthclub) and do some art and yeah.'

For peer review only

COREQ (COnsolidated criteria for REporting Qualitative research) Checklist

Topic	Item No.	Guide questions/Description	Reported on page No
**Domain 1: Research team and reflexivity**			
Personal characteristics			
Interviewer/facilitator	1	Which author/s conducted the interview or focus group?	Page 8 - Methods
Credentials	2	What were the researcher's credentials? E.g. PhD, MD	Submission system
Occupation	3	What was their occupation at the time of the study?	Submission system
Gender	4	Was the researcher male or female?	N/A
Experience and training	5	What experience or training did the researcher have?	Submission system
Relationship with participants			
Relationship established	6	Was a relationship established prior to study commencement?	Page 8 - Methods
Participant knowledge of the interviewer	7	What did the participants know about the researcher? e.g. personal goals, reasons for doing the research	Page 8 - Methods
Interviewer characteristics	8	What characteristics were reported about the interviewer/facilitator? e.g. Bias, assumptions, reasons and interests in the research topic	N/A
**Domain 2: study design**			
Theoretical framework			
Methodological orientation and theory	9	What methodological orientation was stated to underpin the study? e.g. grounded theory, discourse analysis, ethnography, phenomenology, content analysis	Page 7- Methods
Participant selection			
Sampling	10	How were participants selected? e.g. purposive, convenience, consecutive, snowball	Page 8- Methods
Method of approach	11	How were participants approached? e.g. face-to-face, telephone, mail, email	Page 8- Methods
Sample size	12	How many participants were in the study?	Page 10- Results
Non-participation	13	How many people refused to participate or dropped out? Reasons?	N/A
Setting			
Setting of data collection	14	Where was the data collected? e.g. home, clinic, workplace	Page 8- Methods
Presence of non-participants	15	Was anyone else present besides the participants and researchers?	Page 8- Methods
Description of sample	16	What are the important characteristics of the sample? e.g. demographic data, date	Page 10- Results
Data collection			

For peer review only

Interview guide	17	Were questions, prompts, guides provided by the authors? Was it pilot tested?	Page 9- Methods
Repeat interviews	18	Were repeat interviews carried out? If yes, how many?	N/A
Audio/visual recording	19	Did the research use audio or visual recording to collect the data?	Page 9- Methods
Field notes	20	Were field notes made during and/or after the interview or focus group?	N/A
Duration	21	What was the duration of the interviews or focus group?	Page 8- Methods
Data saturation	22	Was data saturation discussed?	N/A
Transcripts returned	23	Were transcripts returned to participants for comment and/or correction?	N/A
**Domain 3: analysis and findings**			
Data analysis			
Number of data coders	24	How many data coders coded the data?	Page 9- Methods
Description of the coding tree	25	Did authors provide a description of the coding tree?	Appendix C
Derivation of themes	26	Were themes identified in advance or derived from the data?	Page 9- Methods
Software	27	What software, if applicable, was used to manage the data?	N/A
Participant checking	28	Did participants provide feedback on the findings?	N/A
Reporting			
Quotations presented	29	Were participant quotations presented to illustrate the themes / findings? Was each quotation identified? e.g. participant number	Pages 11-15- Results
Data and findings consistent	30	Was there consistency between the data presented and the findings?	Pages 11-15- Results
Clarity of major themes	31	Were major themes clearly presented in the findings?	Pages 11-15- Results
Clarity of minor themes	32	Is there a description of diverse cases or discussion of minor themes?	Pages 11-15- Results

Developed from: Tong A, Sainsbury P, Craig J. Consolidated criteria for reporting qualitative research (COREQ): a 32-item checklist for interviews and focus groups. International Journal for Quality in Health Care. 2007. Volume 19, Number 6: pp. 349 – 357

For peer review only
